# Supplementary material for: SDMtoolbox 2.0: the next generation Python-based GIS toolkit for landscape genetic, biogeographic and species distribution model analyses
Source: PeerJ. 2017 Dec 5;5:e4095. doi: 10.7717/peerj.4095 (PMC5721907; doi:10.7717/peerj.4095)
Supplement: Supplemental Information 2 [file peerj-05-4095-s002.pdf]

SDMtoolbox 2.0 User Guide

By Jason L. Brown, Southern Illinois University

Last updated: 9/04/2017

The SDMtoolbox 2.0 consists of a series of python scripts designed to automate complicated ArcGIS analyses that are often too difficult/tedious for the average GIS user to execute efficiently.

This tutorial gives a basic introduction to use SDMtoolbox 2.0. It is a software package for spatial studies of ecology, evolution, and genetics. The release of SDMtoolbox 2.0 allows researchers to use the most current ArcGIS software and Maxent software, and reduces the amount of time that would be spent developing common solutions. The central aim of this software is to automate complicated and repetitive spatial analyses in an intuitive graphical user interface. One core tenant facilitates careful parameterization of species distribution models (SDMs) to maximize each model's discriminatory ability and minimize overfitting. This includes carefully processing of occurrence data, environmental data and model parameterization. This program directly interfaces with Maxent, one of the most powerful and widely used species distribution modeling software programs, although SDMtoolbox 2.0 is not limited to species distribution modeling or restricted to modeling in Maxent. Many of the analyses are also available for use on other SDM methods (see: SDM Tools →1. Universal Tools). The current version contains a total 78 scripts that harness the power of ArcGIS for macroecology, landscape genetics, and evolutionary studies. For example, these tools allow for biodiversity quantification (such as species richness or corrected weighted endemism), generation of least-cost paths and corridors among shared haplotypes, assessment of the significance of spatial randomizations, and enforcement of dispersal limitations of SDMs projected into future climates--- to only name a few functions contained in SDMtoolbox 2.0. Lastly, there exists dozens of generalized tools for batch processing and conversion of GIS data types or formats, which are broadly useful to any ArcMap user.

If you use the SDMtoolbox please remember to cite it. By citing this you encourage the development of more tools.

Please use the following reference:

Brown, J.L., Bennett J., French C.M. SDMtoolbox: the next generation python-based GIS toolkit for landscape genetic, biogeographic and species distribution model analyses. Target Journal: PeerJ

Brown, J.L. (2014) SDMtoolbox: a python-based GIS toolkit for landscape genetic, biogeographic and species distribution model analyses. *Methods in Ecology and Evolution*

Table of Contents

Getting Started 6

Downloading 6

Installation 6

Uninstalling or updating 7

A First Run 8

Analyses Guide 11

Chapter 1. Biodiversity Measurements 15

Estimate Richness and Endemicity (WE and CWE) 15

ARCGIS STEP-BY-STEP GUIDE: 15

SDMTOOLBOX STEP-BY-STEP GUIDE: 17

Results 18

CANAPE categorization (using outputs from Biodiverse) 215

ARCGIS STEP-BY-STEP GUIDE: 15

SDMTOOLBOX STEP-BY-STEP GUIDE: 17

Results 18

Quickly Reclassify Significance from Randomization (Biodiverse) – one tailed 15

ARCGIS STEP-BY-STEP GUIDE: 15

SDMTOOLBOX STEP-BY-STEP GUIDE: 17

Results 18

Chapter 2. Landscape Connectivity 21

Landscape Connectivity 29

ARCGIS STEP-BY-STEP GUIDE: 29

SDMTOOLBOX STEP-BY-STEP GUIDE: 31

Results 33

Create Pairwise Distance Matrix 29

ARCGIS STEP-BY-STEP GUIDE: 29

SDMTOOLBOX STEP-BY-STEP GUIDE: 31

Results 33

Chapter 3. Species Distribution Modeling Tools 38

Correcting Latitudinal Background Selection Bias 38

Solution 1 39

Tool: 1. Bias File for Coordinate Data (BFCD) in MaxEnt 39
ArcGIS Step-by-Step Guide: 39
SDMtoolbox Step-by-Step Guide: 40
Tool: 2a. Background Selection: Sample by Buffered MCP 41
ArcGIS Step-by-Step Guide: 41
SDMtoolbox Step-by-Step Guide: 41
Tool: 3. Clip BFCD by BS Bias File 42
ArcGIS Step-by-Step Guide: 42
SDMtoolbox Step-by-Step Guide: 42
Solution 2 43
Tool: 1. CSV to EAP. MaxEnt format output (runs both 1a and 1b) 43
ArcGIS Step-by-Step Guide: 43
SDMtoolbox Step-by-Step Guide: 43
Tool: 2. Project Climate Data (Raster) to Equal-Area Projection (Folder) 45
ArcGIS Step-by-Step Guide: 45
SDMtoolbox Step-by-Step Guide: 45
Distribution Changes Between Binary SDMs 46
Tool: Centroid Changes (Lines) 46
ArcGIS Step-by-Step Guide: 46
SDMtoolbox Step-by-Step Guide: 47
Tool: Distribution Changes Between Binary SDMs 48
ArcGIS Step-by-Step Guide: 48
SDMtoolbox Step-by-Step Guide: 48
Results 49
Overprediction Correction: Clip Models by Buffered Minimum Convex Polygons 50
Tool: Binary Models (folder) 50
ArcGIS Step-by-Step Guide: 50
SDMtoolbox Step-by-Step Guide: 51
Results 52
Tool: Continuous Models (folder) 53
ArcGIS Step-by-Step Guide: 53
SDMtoolbox Step-by-Step Guide: 53

Results ... 54

Background Selection via Bias Files ... 55

Tool: Background Selection: Sample by Buffered MCP ... 56

ARCGIS STEP-BY-STEP GUIDE: ... 56

SDMTOOLBOX STEP-BY-STEP GUIDE: ... 57

Tool: Background Selection: Sample by Distance from Obs. Pts. ... 57

ARCGIS STEP-BY-STEP GUIDE: ... 57

SDMTOOLBOX STEP-BY-STEP GUIDE: ... 58

Tool: Background Selection: Sample by Buffered Local Adaptive Convext-Hull ... 57

ARCGIS STEP-BY-STEP GUIDE: ... 57

SDMTOOLBOX STEP-BY-STEP GUIDE: ... 58

Create Friction Layer: Invert SDM ... 61

ARCGIS STEP-BY-STEP GUIDE: ... 61

SDMTOOLBOX STEP-BY-STEP GUIDE: ... 62

Results ... 63

Spatially Rarefy Occurrence Data ... 64

ARCGIS STEP-BY-STEP GUIDE: ... 64

SDMTOOLBOX STEP-BY-STEP GUIDE: ... 65

Split Binary SDM by Input Clade Realtionship ... 67

ARCGIS STEP-BY-STEP GUIDE: ... 67

SDMTOOLBOX STEP-BY-STEP GUIDE: ... 68

Results ... 63

Chapter 4. Basic Tools: Raster, CSV & Shapefile tools ... 70

Tool: *CSV to shapefile* ... 70

ARCGIS STEP-BY-STEP GUIDE: ... 70

SDMTOOLBOX STEP-BY-STEP GUIDE: ... 70

Chapter 5. Running a SDM in MaxEnt: from start to finish ... 71

Data Preparation ... 71

1. Preparing Worldclim Data ... 71

2. Testing Autocorrelations of Environmental Data ... 74

3. Preparing Occurrence Data ... 81

4. Creation of Bias Files…… 83

Model Creation, Calibration and Evaluation……..85

5. Spatial Jackknifing ……..86

Chapter 6. Frequently asked questions and misconceptions regarding SDMtoolbox & Maxent……….90

Getting Started

Downloading

The latest version of the toolbox is available for download at: www.sdmtoolbox.org. This software requires ArcMap 10.1-10.5 with an active Spatial Analyst license (www.ESRI.com). This toolbox is programed specifically for ArcMap 10.1 (and above) and due to a series of improvements in this version, it is not backwards compatible with older releases of ArcMap (*i.e.* ArcMap 9.2). This software consists of an ArcGIS toolbox and associated python scripts.

Installation

1. Download, unzip 'SDMtoolbox.zip' and place all files in a folder on your harddrive where you want the toolbox to be stored (e.g ...\Documents\ArcGIStoolboxes)

2. Close all ArcGIS programs

3. Open **ArcCatalog 10.X** (a program in the ESRI ArcGIS programs, see icon below)

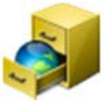

Above: ArcCatalog Icon

4. Once open, activate the ArcToolbox window (if not already visible) by clicking the red box below

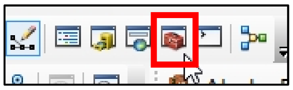

5. Then right-click ArcToolbox Window (see image below)→ select 'Add Toolbox.' Then go to location of downloaded toolbox and select 'SDMToolbox v2.0.tbx'

6. The toolbox should appear inside the ArcToolbox (see highlighted toolbox to right). If there, now the toolbox is almost installed.

7. To finish installation simply close the ArcCatalog program.

8. Now start ArcMap and begin using the SDMtoobox!

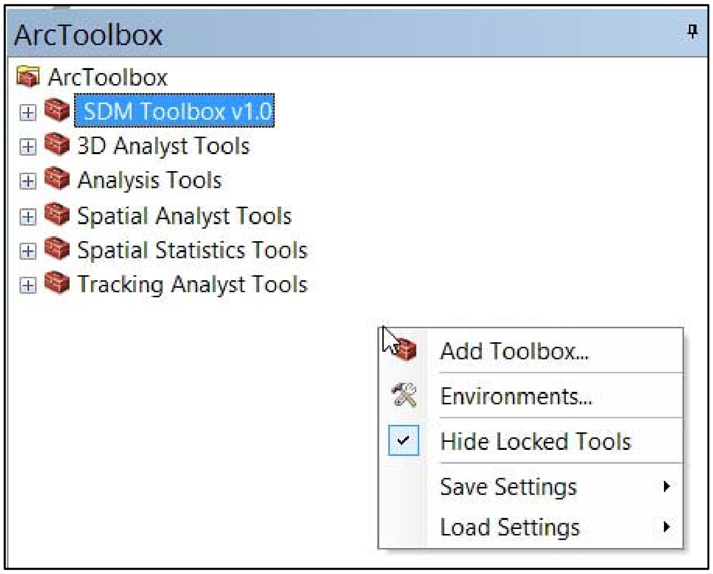

How to update or uninstall SDMtoolbox

1. Close all ArcGIS programs

2. Open **ArcCatalog 10** (a program in the ESRI ArcGIS programs, see icon below)

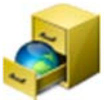

Above: ArcCatalog Icon

3. Once open, activate the ArcToolbox window (if not already visible) by clicking the red box below

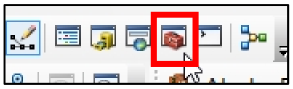

4. Then right-click SDMtoollbox in ArcToolbox Window (see Image to right) and select 'Remove'.

5. If only uninstalling, then you are done. If installing a new version of SDMtoolbox then right-click ArcToolbox Window (see image below)→ select 'Add Toolbox.' Then go to location of downloaded toolbox and select 'SDMToolbox v1.X.tbx'

6. The toolbox should appear inside the ArcToolbox (see highlighted toolbox to right). If there, now the toolbox is almost installed.

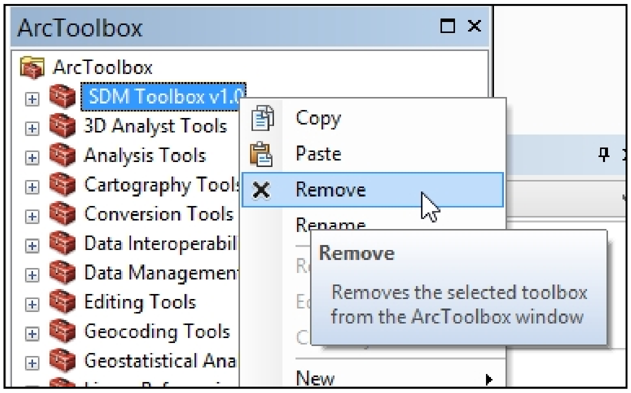

7. To finish installation simply close the ArcCatalog program.

8. Now start ArcMap and begin using the SDMtoobox!

A First Run

Many things will cause the SDMtoolbox to *not* run that have *nothing* to do with the toolbox itself. Upon first use, the following steps should be performed:

1. **Open ArcMap10.X and activate the ArcToolbox window (if not already visible). Bottom image on this page is the Arctoolbox window. If not visible, click toolbox icon (see image below).**

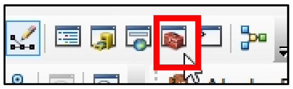

2. **Ensure Spatial Analyst is Enabled in ArcMap**

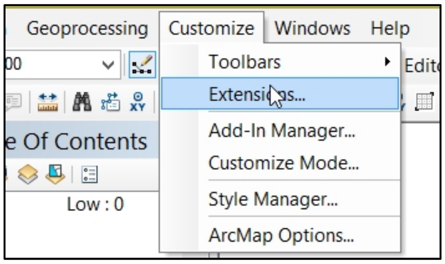

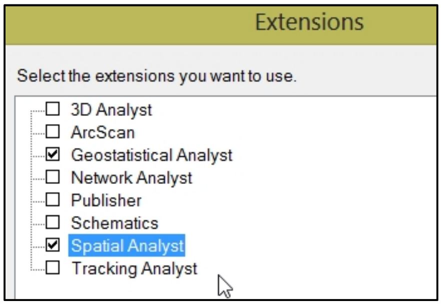

A. Go to: Customize→ Extensions

B. Check the box next to: *Spatial Analyst* (and *Geostatistical Analyst* if available)

3. **Install all relevant ArcMap 10.X Patches and Service Packs.**

   The base release of ArcMap 10.1/2 has some serious bugs that cause many analyses to fail. The only way to fix these bugs is the install all relevant patches and service packs.

   A. To search for these go to:

   http://support.esri.com/en/downloads/patches-servicepacks/list?productid=160&productVersions=10.1&categoryTypes=5&categoryTypes=8

   Or: http://support.esri.com/en/downloads/patches-servicepacks

   B. Or use the ESRI's 'Patch Finder for Windows' to identify patches, then search for suggested patches/service packs (http://downloads2.esri.com/Support/downloads/other_/PatchFinder.exe)

4. **Enable 'Tool Help' and resize windows.**

   Open the 'Basic Tools →Raster Tools → 2a. Raster to ASCII (Folder)' tool. Double-Click the tool (see image to right)

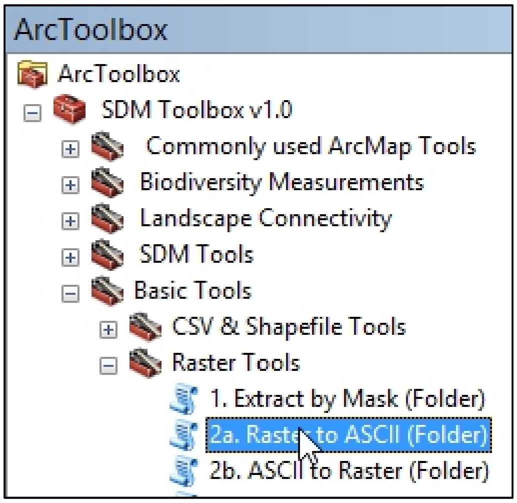

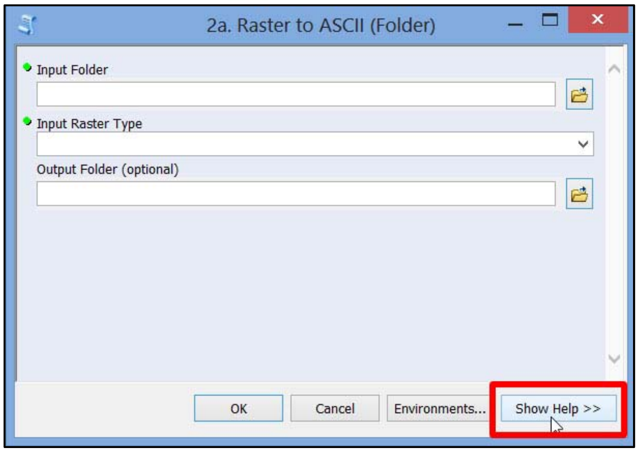

A. In the newly opened tool click 'Show Help' (red box above)

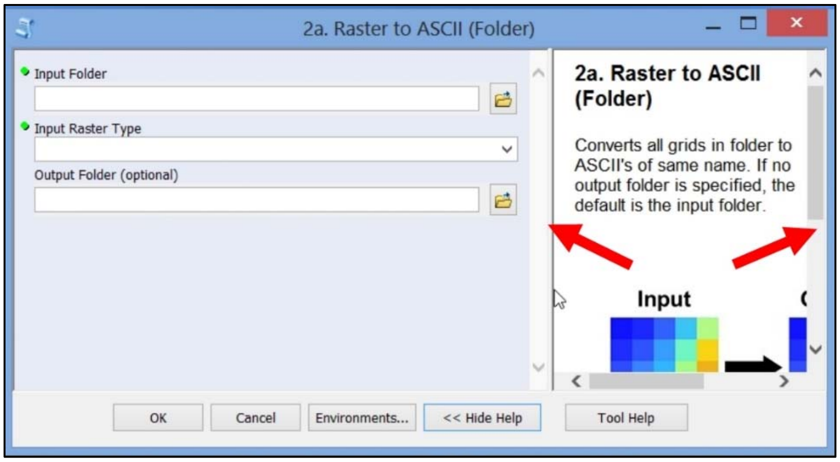

B. Resize the window by dragging the two windows (depicted by red arrows) so that the final tool window is as below. Note the SDMtoolbox Icon should be fully visible. Resizing the help window to this size will ensure that all diagrams are completely visible.

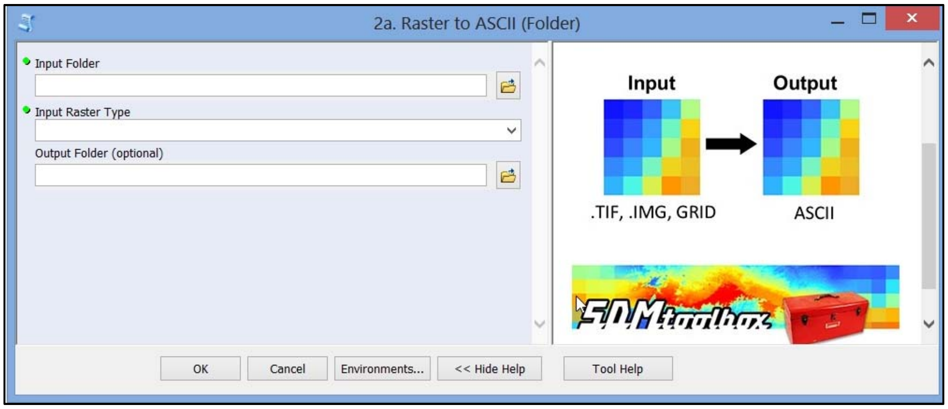

A note on map projections

Most user reported errors have to do with map projections. As follows is a checklist of things you need to check if you have a script fail. In most cases your data should be projected, with the projection specified in the GIS file.

1. **If you change or define the map projection** after opening the map document. You NEED to save all projected files, then close the map document and **open a new map session**. The first file open in your new session needs to be one of the newly projected files—this defines the map datum. This is particularly important if you change the spatial units as the result of projection, e.g. decimal degrees to meters. If you do not do this, often SDMtoolbox scripts will fail or result in inaccurate results.

2. **If the tool requires a CSV of coordinates** AND your analyses are going to be **in another projection**, remember to convert input coordinates (or XYs) to the new, and same, datum prior to running tools or for use in species distribution modeling.

   For most, the quickest way to do this for CSV files (and other tables) is to:

   1) Import the CSV to a shape file (*Table & Shapefile Tools* → *CSV to shapefile* tool)
   2) Define input projection of shapefile and then project to the new projection (a. ArcGIS *define projection* and b. *project* tools)
   3) Add the new coordinates/XYs to Shapefile using the ArcGIS *Add XYs* tool
   4) Export shapefile table as CSV file (SDMtoolbox the *Table & Shapefile Tools* →*Shapefile to CSV* tool)

   If using an equal-areas projection and input data are WGS-1984 coordinates, try the SDMtoolbox tool that will do all the above steps:

   SDM tools → 2. MaxEnt Tools→Correcting Latitudinal Background Selection Biases→ Solution 2: Project Input Data to Equal-Area Projection (EAP)→ 1. CSV to EAP. MaxEnt format output

3. Check that ***all* your input data are projected and in the *same* projection**. This is the most common error. Often reference ASCII files are not projected at all.
4. Last, **visualize all GIS files** to make sure they are in fact properly projected (all maps should overlap etc.). Sometimes files say they are projected to the same projection, however in reality something was incorrect.

To check the projection of each GIS file

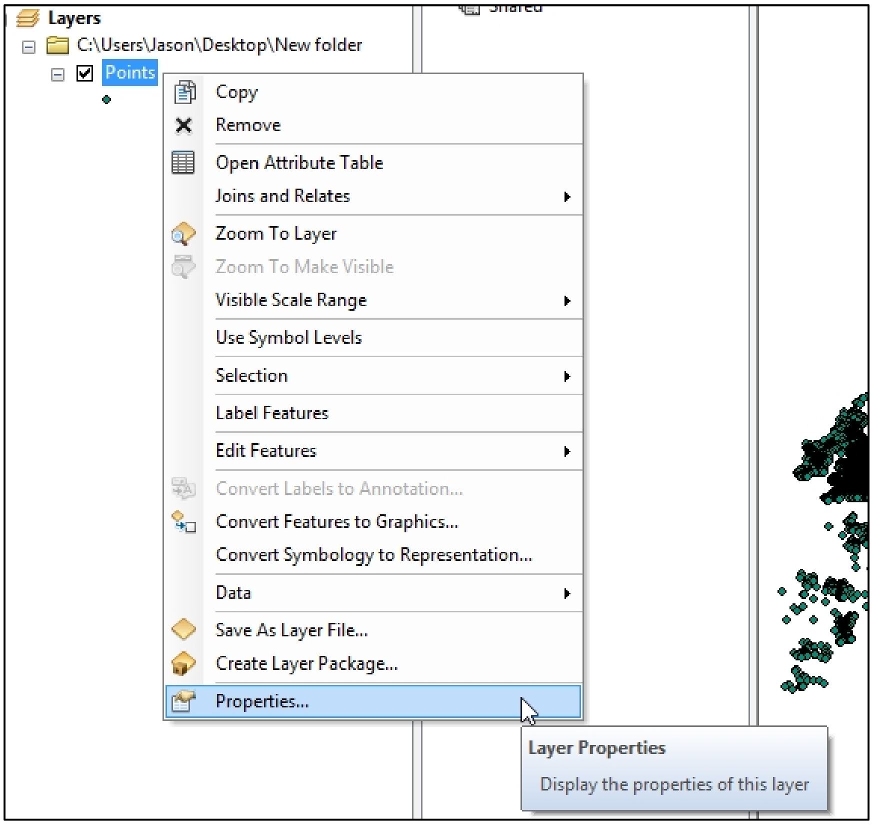

Import GIS files, then right click each file and select 'Properties'

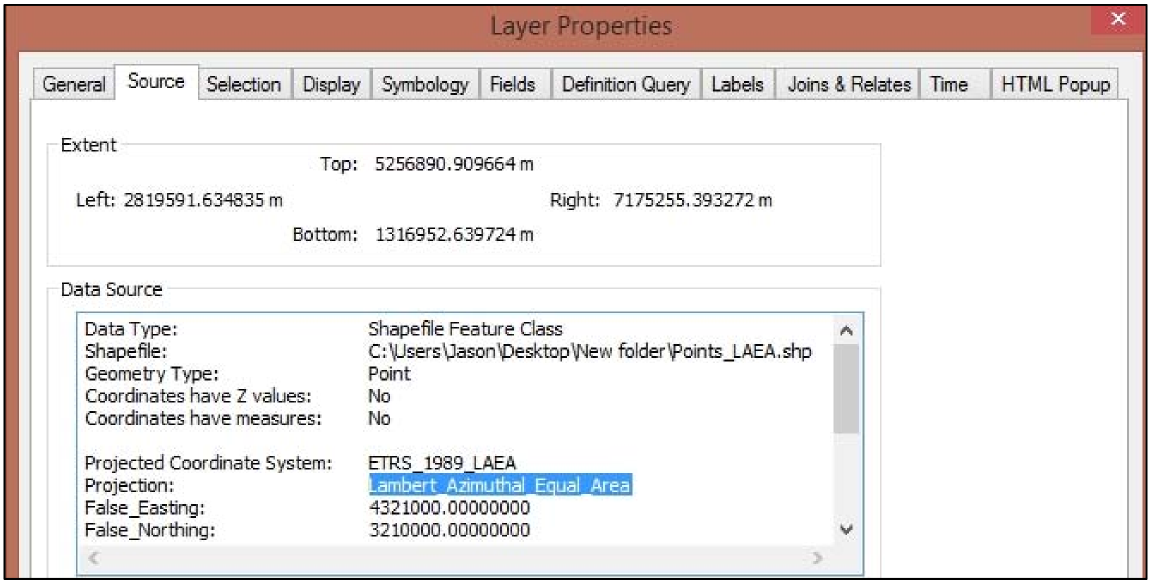

Select the 'Source' tab and page down until you see the 'Projection'. Here the projection is Lamberts Azimuthal Equal Area

To accompany this guide I have provided example data available at www.sdmtoolbox.org. Download this, the latest version of the SDMtoolbox, and this guide before beginning.

This guide does not cover all the tools in the SDMtoolbox. However, as an overview, the guide covers tools from all the major groups. For many tools lacking a guide I have included example data to execute each analysis. The example data should be contained in a folder respective to the hierarchy of the toolbox. For example, for the tool 'SDM toolbox: Biodiversity Measurements → Input: Point Data → Calculate Richness and Endemicity (WE and CWE)', the example data are in the folder '…example_data\biodiversity_measurements\biodiversity_points'

Lastly, each tool is annotated and instructions should be contained within each tool's help file from within ArcGIS. Follows are the major groups of the toolbox. The guide treats each one as a separate chapter.

The 10 commandments of SDMtoolbox 2.0

1. Thou shall not include spaces in path or file names
2. Thou shall avoid non-alphanumeric characters in file names, headings and table values
    - avoid: * : \ / < > | " ? [ ] ; = + & £ $, etc
3. Thou shall have map projection defined
    - make sure all input data are properly projected and in the *same* projection (also see 'A First Run' for details on this)
4. Thou shall limit table headings to 12 or less characters
    - Sometimes scripts use table headings, often ArcGIS reduces these name to 12 (or less) characters in analyses and confuses its self or input code that calls the full name
5. Thou shall not install an alternative version of Python
6. Thou shall have an appropriate ArcGIS license
    - For full functionality, ArcMap version **MUST** be Standard or Advance license level with Spatial Analyst Extension
7. Thou shall try provided example data corresponding to tool
    - If the example data work, but yours do not, there is a problem with your input parameters or files- compare your inputs to the provided inputs
8. Thou shall read SDMtoolbox error messages
    - The red text is ArcMap's error text, SDMtoolbox often has its own more, specific error codes preceding (highlighted below)

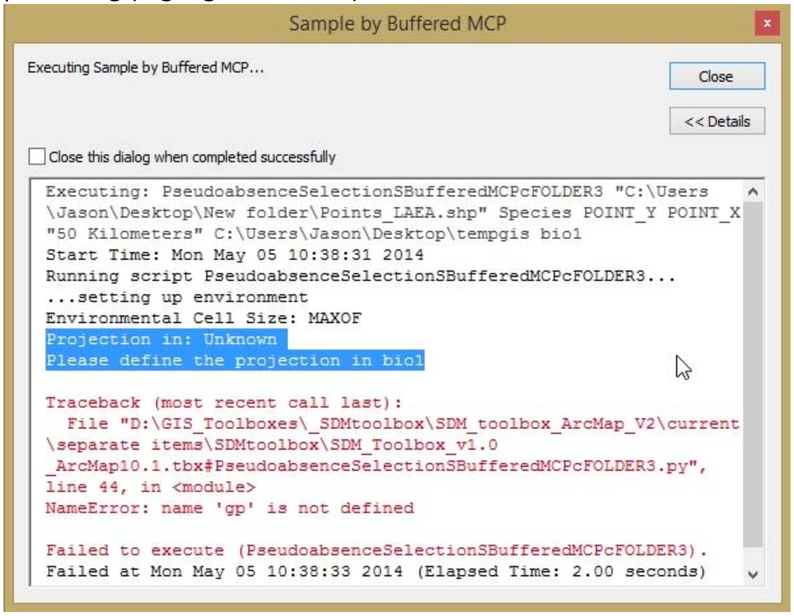

9. When a tool fails, thou shall not rerun analyses without deleting all files in output folder or changing to a new output folder.
10. Thou shall remedy all these items before emailing SDMtoobox. When emailing SDMtoolbox, thou shall include detailed information regarding the problem(s), include screenshots of errors and information regarding input data
    - I usually cannot help you with out this information

Toolbox Guide

Chapter Structure

1. **Biodiversity Measurements**
2. **Landscape Connectivity**
3. **SDM Tools**
4. **Basic Tools (Both CSV & Shapefile tools and Raster tools)**
5. **Running a SDM in MaxEnt: from start to finish**
6. **Frequently asked questions and misconceptions regarding SDMtoolbox & Maxent**

Chapter 1. Biodiversity Measurements

Tool Overview

These tools estimate three common biodiversity metrics: species richness, weighted endemism and corrected weighted endemism. There are two sets of analyses here: analyses that utilize point occurrence data and analyses that use binary SDMs.

As follows are the three diversity metrics:

1. **Species Richness** (SR) is sum of unique species per cell.

   SR = K (the total number of species in a grid cell)

2. **Weighted Endemism** (WE), which is the sum of the reciprocal of the total number of cells each species in a grid cell is found in. A WE emphasizes areas that have a high proportion of animals with restricted ranges.

   $WE = \sum 1/C$ (C is the number of grid cells each endemic occurs in)

3. **Corrected Weighted Endemism** (CWE). The corrected weighted endemism is simply the weighted endemism divided by the total number of species in a cell (Crisp 2001). A CWE emphasizes areas that have a high proportion of animals with restricted ranges, but are not necessarily areas that are species rich.

   $CWE = WE/K$ (K is the total number of species in a grid cell)

Crisp, M. D., Laffan, S., Linder, H. P., and Monro, A. 2001. Endemism in the Australian flora. *Journal of Biogeography* 28:183-198.

Estimate Richness and Endemicity (WE and CWE)

ArcGIS Step-by-Step Guide:

1. Open a fresh ArcMap document
2. Import ‘species_not_modeled.shp’ and ‘mask.shp’ (Location: …\example_data\biodiversity_measurements\biodiversity_binary_SDMs)

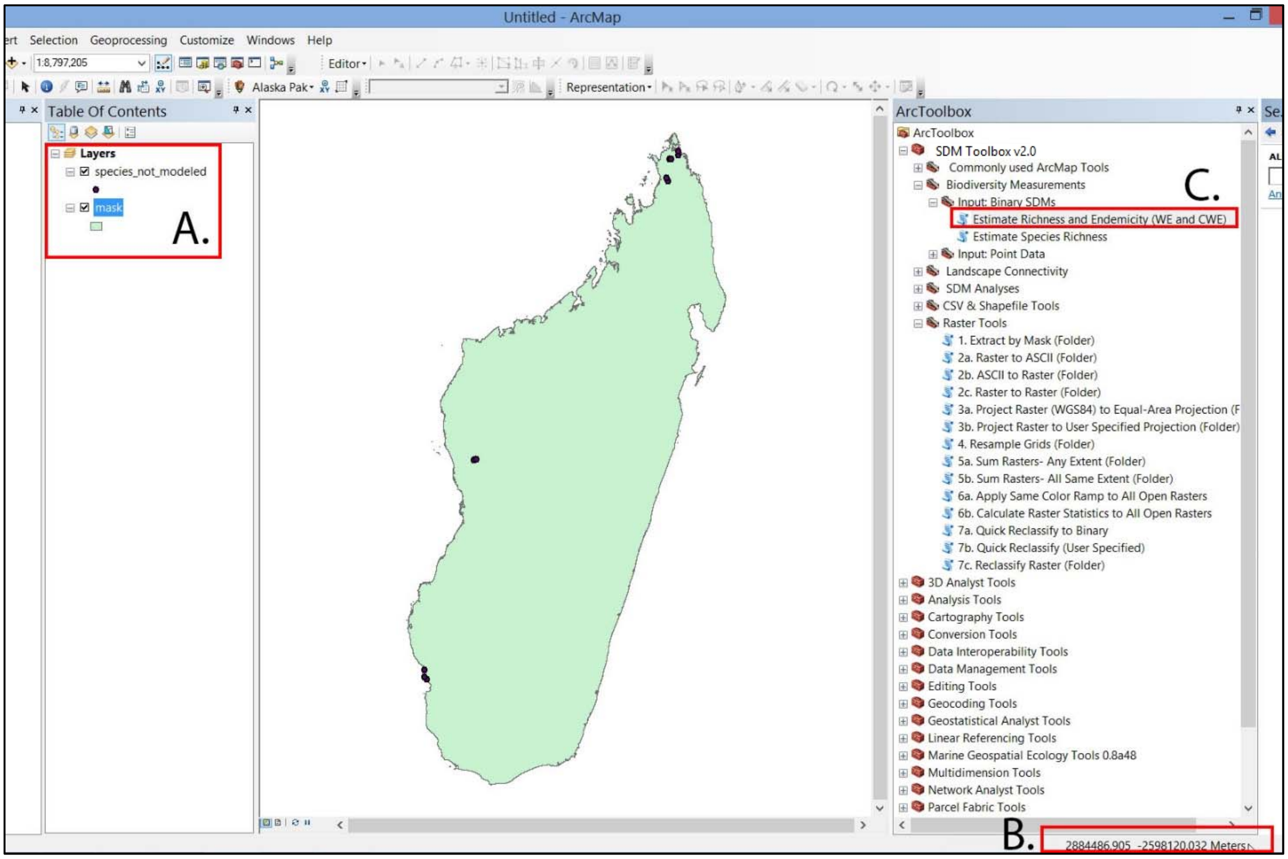

A. Imported data. **B.** Note that these data units are meters **C.** Tool to be used in this guide

3. Next double-click the 'Biodiversity Measurements → Input Binary SDMs → Output: Grid → Estimate Richness and Edemicity (WE and CWE)' tool
4. Continue to tool interface instructions (following page)

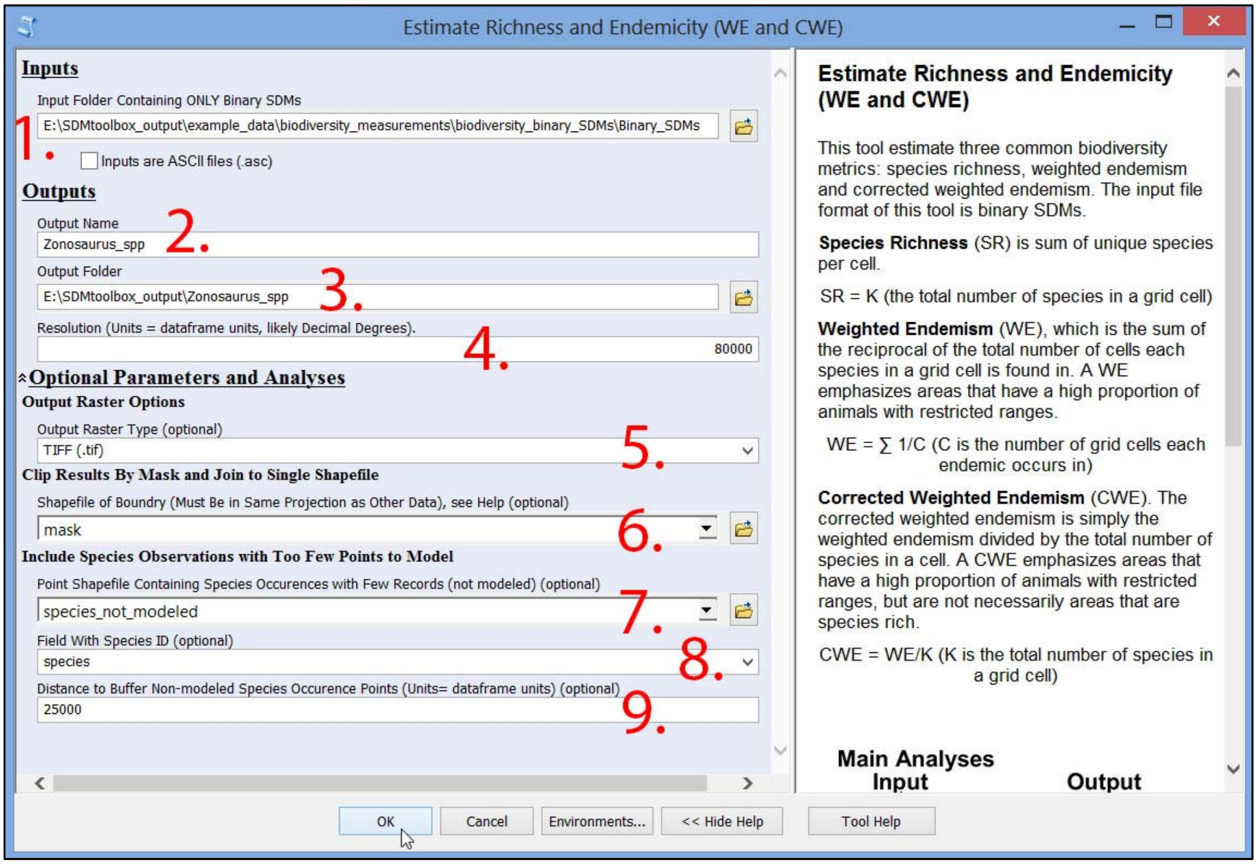

***Estimate Richness and Endemicty (WE and CWE)* tool interface**

SDMTOOLBOX STEP-BY-STEP GUIDE:

1. Select example data folder:
   …\example_data\biodiversity_measurements\biodiversity_binary_SDMs\Binary_SDMs

   Leave the 'Inputs are ASCII files (.asc)' unchecked. Here the inputs are TIFF raster files.

2. Input desired output name. Note when this category is highlighted the Help box displays the text in the box to the right. I input 'Uruloke_spp', this means that the output files will be named: 'Uruloke_spp_Estimated_Spp_Rich.tif', 'Uruloke _spp_Est_Spp_Rich_Low_Res.tif', 'Uruloke _spp_WE.tif' and 'Uruloke_spp_CWE.tif'.

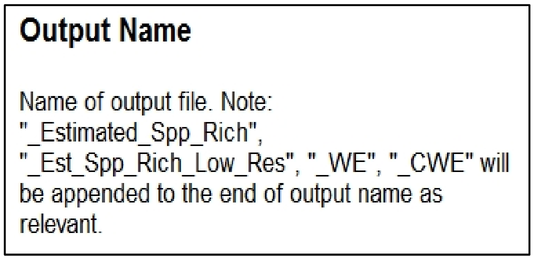

3. Select output folder location. This should be a new empty folder. If not empty, this can cause the analysis to fail, particularly if temporary files from a previous analysis were not properly removed (e.g., this can happen if another SDMtoolbox analysis is terminated early).

4. Here I used a resolution of 80,000m (or 80km). The data used here are in meters. In the future, however, your data might being in feet or degrees. The distance value input should be large enough to capture landscape processes, but not too large where regional differences are lost. I recommend starting with a value equivalent to 50 or 100km. Note 1: your actual values will be in the maps units (likely meters, feet or decimal degrees). Note 2: 100km = ~0.8983 DD at Equator.
5. I prefer TIFF files as a raster output format because they allow for longer file names (vs. ESRI grid files that are limited to 13 characters) and don't have too many raw parts to each file. There is, however, a slight reduction in performance (vs. ESRI grid files), thus, if processing thousands of rasters this should be taken into consideration.
6. A polygon mask of a country outline. This will clip the edges of pixels by the boundary of this mask. This produces a much more visually pleasing output. All biodiversity metrics will be appended to the shapefile table and can be visualized in the file symbology.

> NOTES:
> -This MUST be in the same projection as other input data (e.g. all projections must be WGS 1984).
> -A good source for clipping mask (country boundaries etc.) is: http://www.diva-gis.org/Data
> -Again, be sure to project the file to match your input rasters

7. Often due to lacking occurrence data, not all species can be modeled. This feature will include species occurrences that were not modeled to be included in the biodiversity estimates.
8. Select table field corresponding to species ID
9. Buffer distance. The input points will be buffered to this distance. Here I chose 25,000m (25km)--- this means a circle with a 25km radius will be created around each point.

Results

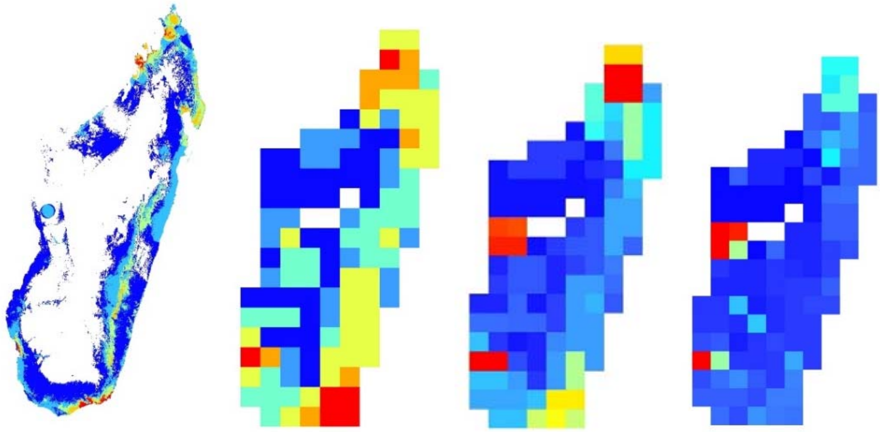

Outputs from analyses. Left to Right: Estimated Spp. Richness, Estimated Spp. Richness (low resolution), WE and CWE

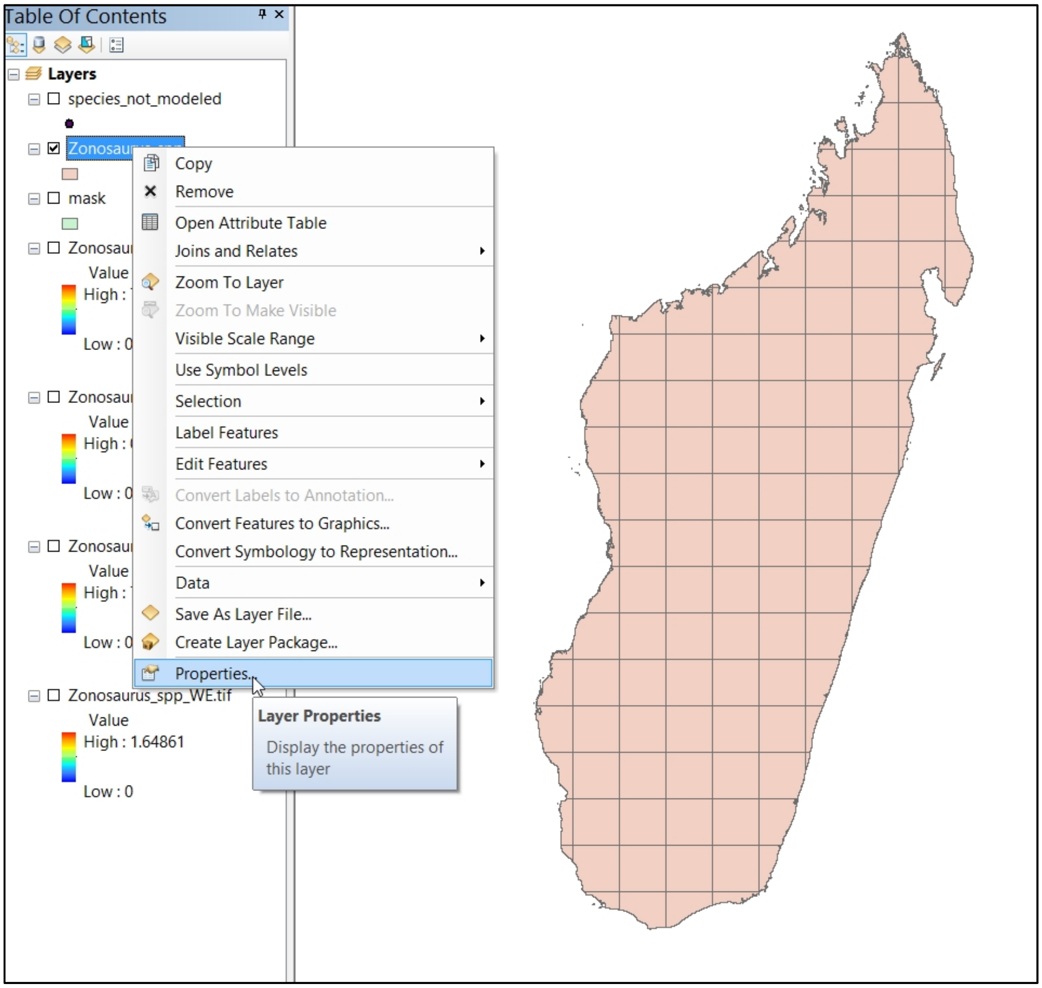

To change the appearance of the output shapefile, right click the file and select 'Properties'

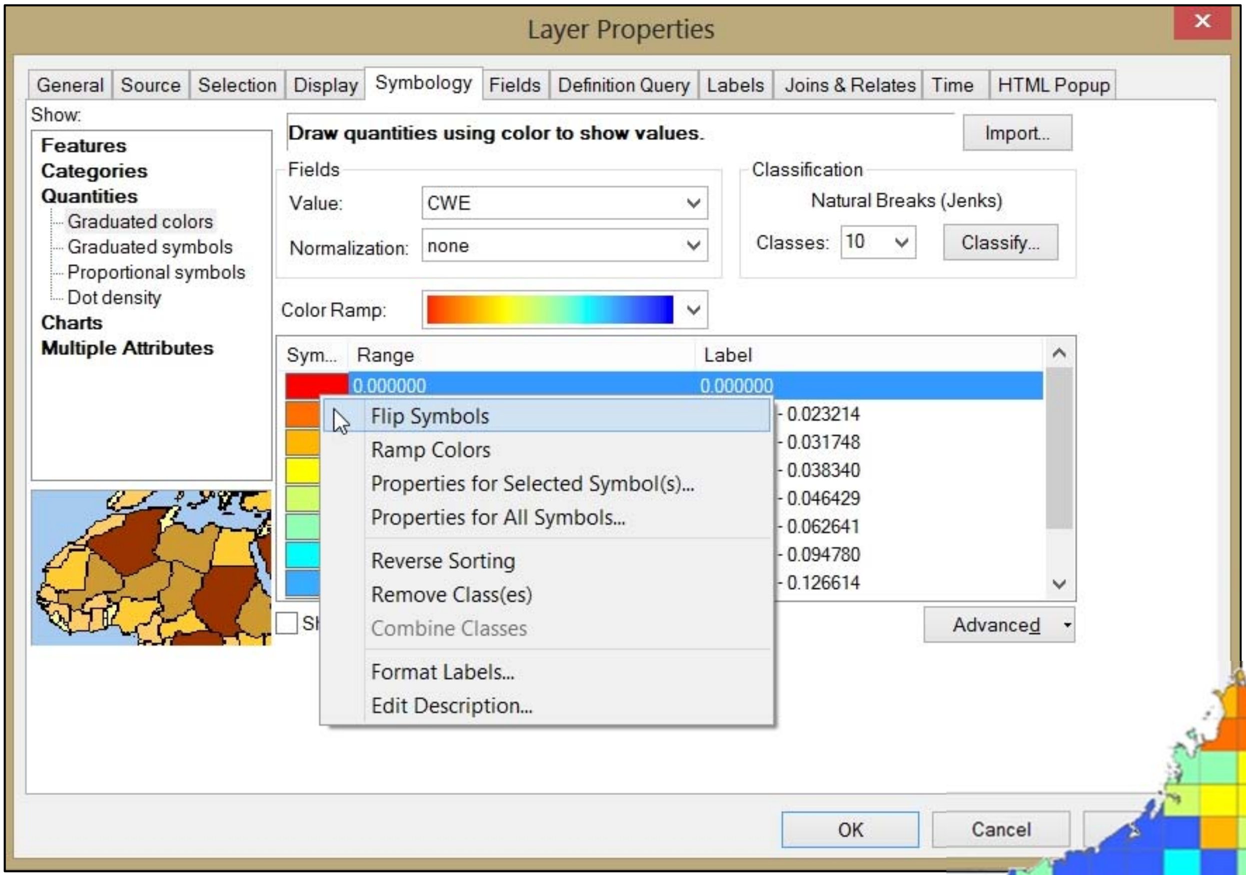

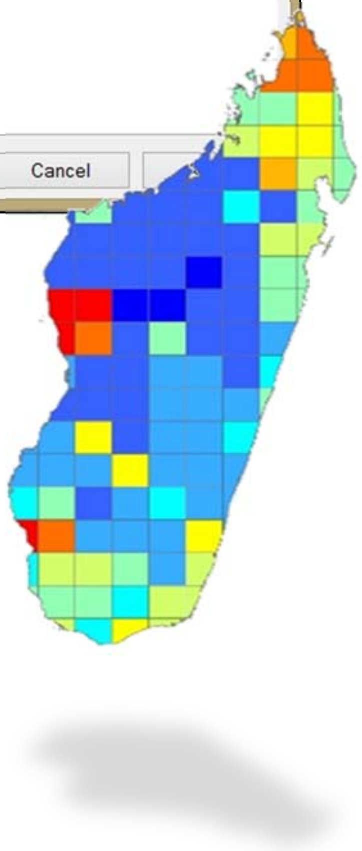

Then select the 'Symbology' tab. In the left column select 'Quantities → Graduated colors', for 'Fields Value' select any of the three biodiversity fields. Here I chose to use 10 classes defined by 'Natural Breaks (Jenks).' To keep consistent with the other color schemes, I used the RGB color ramp and inverted it so that the highest values are red. To do this, right click one of the colors (see above) and select 'Flip Symbols.' Small image to right is the output from these settings.

Extra bit: For a little extra pizazz, place a digital elevation model below the biodiversity layer (use black to white colorramp) and make the biodiversity layer 10-20% transparent. This gives the map a little texture corresponding to topography.

Chapter 1. CANAPE Categorization (using outputs from Biodiverse)

Tool Overview

This tool facilitates a quantitative method for locating hotspots of endemism called Categorical Analysis of Neo- and Paleo-Endemism (CANAPE; Mishler et al. 2014) on grids output from Biodiverse (http://shawnlaffan.github.io/biodiverse/). These analyses are able to classify neo-endemic and paleo-endemic species, young taxon and an old taxon with a restricted distribution, respectively. This method assess the significance of branch lengths among taxa that are either significantly shorter (neo) or significantly longer (paleo) than other areas in the landscape.

For more information regarding this analysis and running it: http://biodiverse-analysis-software.blogspot.com.au/

CANAPE Categorization (using outputs from Biodiverse)

ARCGIS STEP-BY-STEP GUIDE:

1. Open a fresh ArcMap document
2. Import 'C_PE_WE_P.tif', 'C_PHYLO_RPE2.tif' and 'C_PHYLO_RPE_NULL2.tif' (Location: …\example_data\biodiversity_measurements\Biodiverse_1000_rand\CANAPE\)
3. Next double-click the 'Biodiversity Measurements →Biodiverse Randomization →CANAPE categorization (using outputs from Biodiverse)' tool
4. Continue to the tool interface instructions (following page)

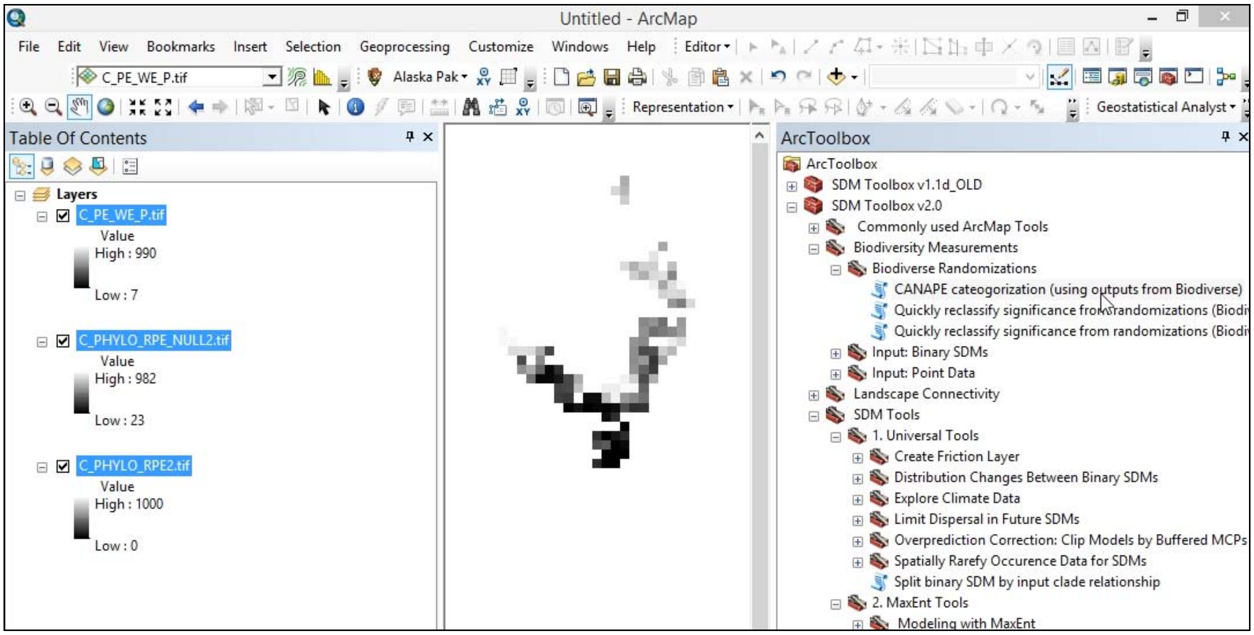

Imported data

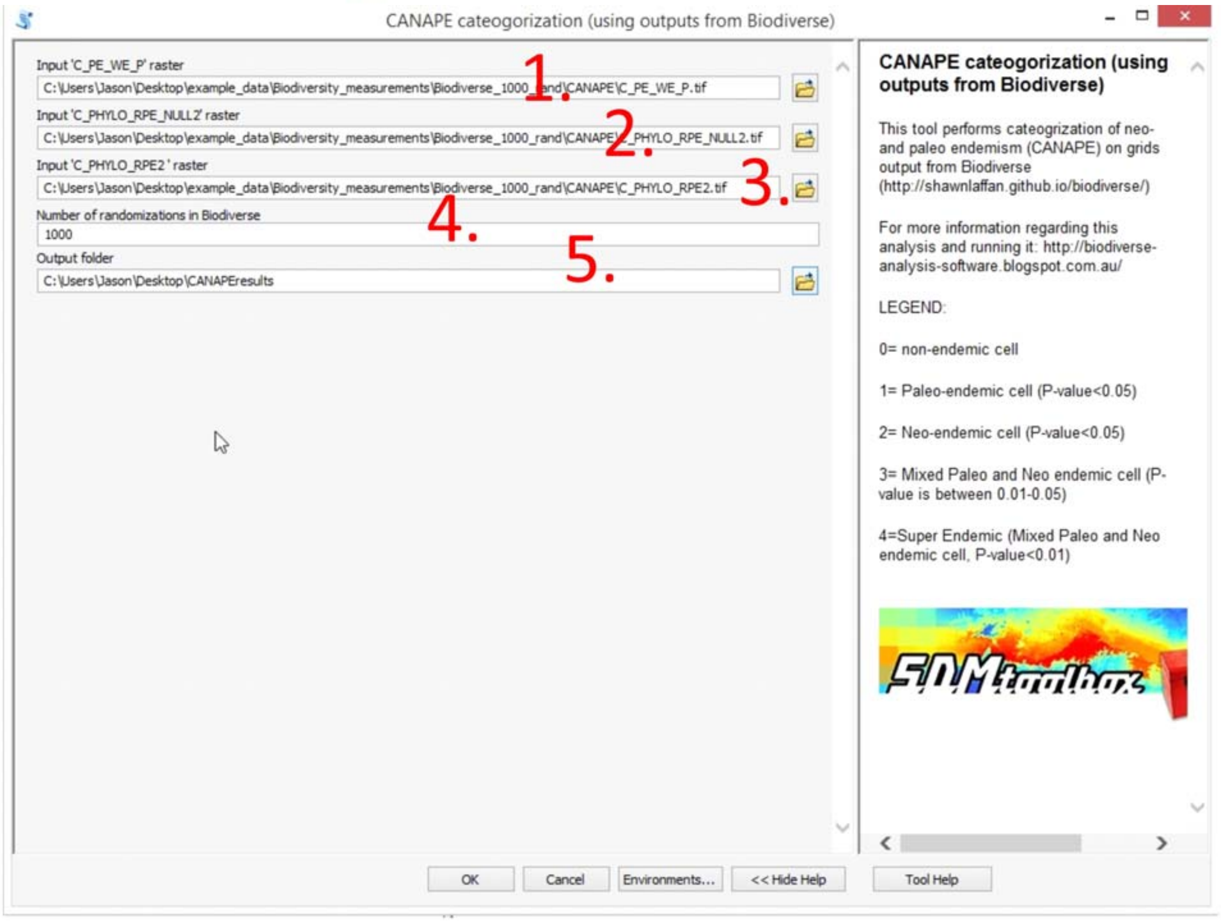

SDMTOOLBOX STEP-BY-STEP GUIDE:

1. Input raster file output from Biodiverse ending in "…_PE_WE_P.tif"
2. Input raster file output from Biodiverse ending in "…_PHYLO_RPE_NULL2.tif"
3. Input raster file output from Biodiverse ending in "…_PHYLO_RPE2.tif"
4. Input the number of randomizations performed in Biodiverse
5. This should be a new empty folder. If not empty this can cause the analysis to fail, particularly if temporary files from a previous analysis were not properly removed (e.g. this can happen if another SDMtoolbox analysis is terminated early).

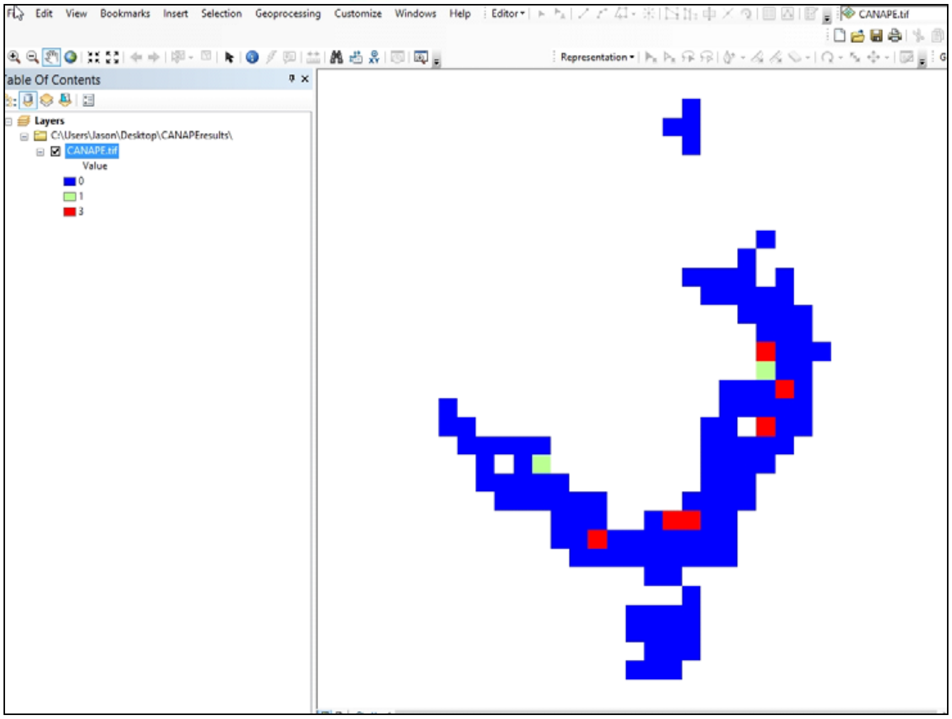

Results

CANAPE Results

LEGEND:
0= non-endemic cell
1= Paleo-endemic cell (P-value<0.05)
2= Neo-endemic cell (P-value<0.05)
3= Mixed Paleo and Neo endemic cell (P-value is between 0.01-0.05)
4=Super Endemic (Mixed Paleo and Neo endemic cell, P-value<0.01)

Chapter 1. Quickly Reclassify Significance from Randomization (Biodiverse) – one tailed

Tool Overview

This tool reclassifies significance from randomizations of all types out from Biodiverse (http://shawnlaffan.github.io/biodiverse/)

For more information regarding this analysis and running it: http://biodiverse-analysis-software.blogspot.com.au/

Quickly Reclassify Significance from Randomization (Biodiverse) – one tailed

ArcGIS Step-by-Step Guide:

1. Open a fresh ArcMap document
2. Import 'Randomization.tif' (Location: …\example_data\biodiversity_measurements\Biodiverse_1000_rand\Typical_Randomization\)
3. Next double-click the 'Biodiversity Measurements →Biodiverse Randomization →Quickly Reclassify Significance from Randomization (Biodiverse) --- one tailed' tool
4. Continue to the tool interface instructions (below)

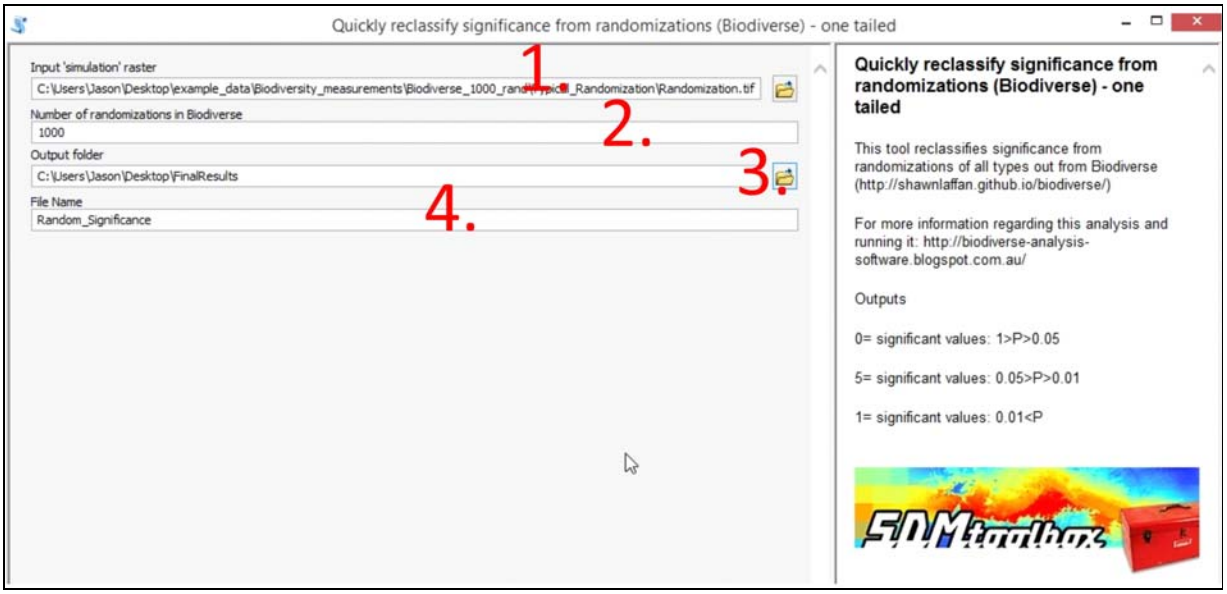

SDMtoolbox Step-by-Step Guide:

1. Input raster file output from Biodiverse ending in "…_Randomization.tif"
2. Input the number of randomizations performed in Biodiverse
3. This should be a new empty folder. If not empty this can cause the analysis to fail, particularly if temporary files from a previous analysis were not properly removed (e.g. this can happen if another SDMtoolbox analysis is terminated early).
4. Output file name. **Tip.** Avoid non-alphanumeric characters in both names (e.g., avoid: * : \ / < > | " ? [ ] ; = + & £ $, etc.).

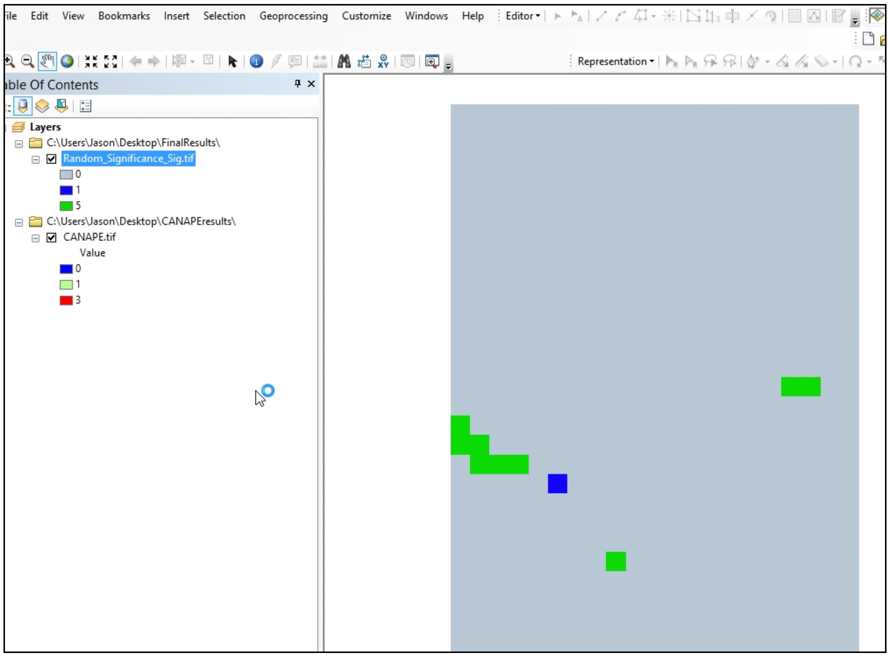

Results

Quickly Reclassify Significance from Randomization (Biodiverse) – one tailed outputs

LEGEND:
0= significant values: 1>P>0.05
5= significant values: 0.05>P>0.01
1= significant values: 0.01<P

Chapter 2. Landscape Connectivity

Tool Overview

This tool creates a raster of the sum of least-cost corridors and a polyline shapefile of least-cost paths between populations that share haplotypes. Often a single LCP between sites oversimplifies landscape processes. By using categories of cost paths that include paths with slightly more costly path lengths (relative to the LCP), you can better depict habitat heterogeneity and its varying role in dispersal. For each comparison you can classify the lowest cost paths into three categories. Lastly, a density analysis will produce a raster depicting the frequency that LCPs traverse the same path.

For more information, see: Chan LM, Brown JL, Yoder AD (2011). Integrating statistical genetic and geospatial methods brings new power to phylogeography. Mol Phylogenet Evol 59(2):523-37. doi: 10.1016/j.ympev.2011.01.020.

Landscape Connectivity

ArcGIS Step-by-Step Guide:

1. Open a fresh ArcMap document
2. Import 'species_not_modeled.shp' and 'mask.shp' (Location: …\example_data\landscape_connectivity\Oplurus_Haplotypes.shp)
3. Next double-click the 'Landscape Connectivity →Genetic → Shared Haplotypes→ Calculate Least-Cost Corridors' tool
4. Continue to the tool interface instructions (following page)

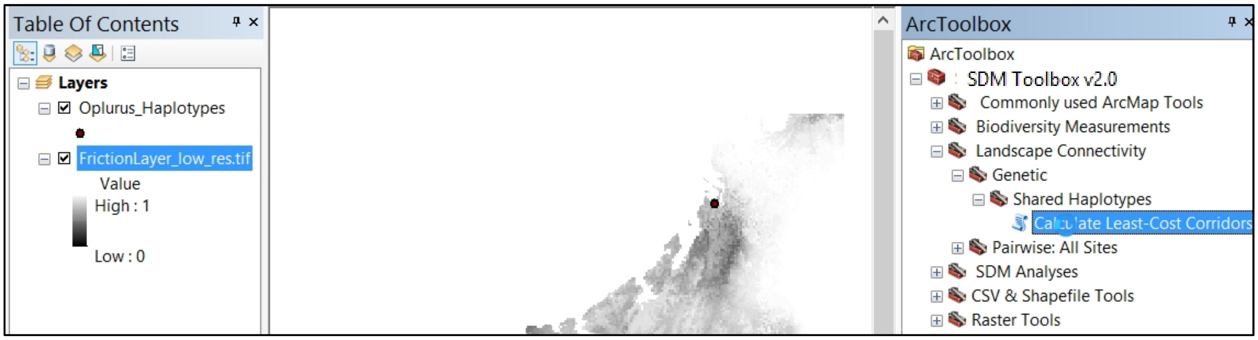

Imported data

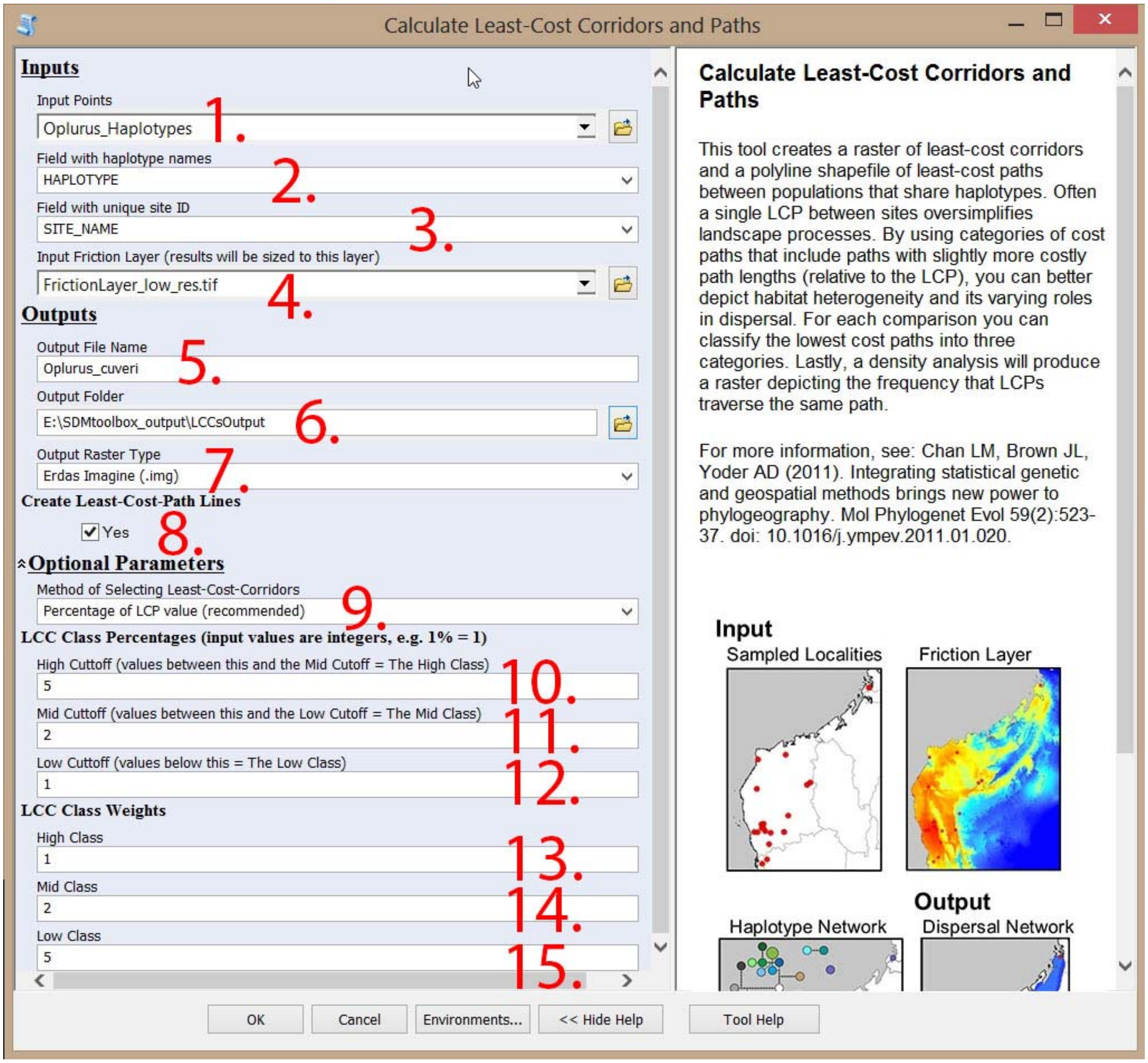

Calculate Least-Cost Corridors and Paths **tool interface**

SDMTOOLBOX STEP-BY-STEP GUIDE:

1. Input point shapefile containing sites and haplotypes to be included in least-cost paths and corridors. This shapefile must have two columns: one depicting site names and another depicting haplotype ID.

LATITUDE	LONGITUDE	SITE_NAME	HAPLOTYPE
-16.38	45.345	1432680	C
-14.31	47.915	1569175	A
-16.525	44.49	1660120	A
-16.525	44.49	1660120	C
-16.525	44.49	1660120	F
-19.75	44.6167	APR1031	B
-18.7967	44.8817	HER2558	B
-18.6533	44.6583	HER2725	D
-18.6533	44.6583	HER2725	E

Required geospatial and haplotype data for analysis

> **Tip.** If you need to convert a table to a shapefile for use here. You need two extra columns (latitude and longitude, see table above). Then use the tool 'CSV & Shapefile Tools → 1. CSV to Shapefile'
> -Avoid non-alphanumeric characters in both names (e.g., avoid: * : \ / < > | " ? [ ] ; = + & £ $, etc.).

2. Table field (part of shapefile) depicting haplotype ID
3. Table field (part of shapefile) depicting site ID. Note this cannot be only a number. If a number recode as alphanumeric ID (e.g. change '9' to '9a')
4. A friction Layer is a raster that depicts the ease of dispersal from each locality through the landscape. In this analysis, the friction layer depicts the output extent and spatial resolution of analysis. If you want a larger extent or a lower spatial resolution, increase both in your friction layer. However, the larger both are—the longer the analysis will take.

> **Output File Name**
> Name of output files. Note: "_Dispersal_Network", "_LCPs.shp", "_LCPs_Line_Density" will be appended to the end of in output name as relevant.

> **Creating a friction layer.** One prevalent way to create a friction layer that doesn't suffer from applying weights to habitat types (often associated with contributing to biases in results) is the use of SDMs. An SDMtoolbox tool (location below) will invert a SDM for use as a friction surface. Using this method, areas of high suitability will be converted to areas of low dispersal cost.
>
> *Tool Path: SDM Tools →1. Universal Tools → Create Friction Layer → Invert SDM*

5. Input desired output name. Note when this category is highlighted the Help Box displays the text in the box to the right. I input 'Oplurus_cuvieri', this means that the output files will be named: 'Oplurus_cuvieri_Dispersal_Network', 'Oplurus_cuvieri_LCPs.shp', 'Oplurus_cuvieri_LCPs_Line_Density'
6. Select output folder location. This should be a new empty folder. If not empty this can cause the analysis to fail, particularly if temporary files from a previous analysis were not properly removed (e.g. this can happen if another SDMtoolbox analysis is terminated early).
7. Output file type. Here I selected the 'Erdas Imagine (.img)' raster format.

> **Tip.** I prefer TIFF files as output format because they allow for longer file names (vs. ESRI grid files that are limited to 13 characters) and don't have too many raw parts to each file. There is, however, a slight reduction in performance (vs. ESRI grid files), thus, if processing thousands of rasters this should be taken into consideration.

8. Create least-cost path lines. Least-cost path analysis (LCP) allows researchers to find the 'cheapest' way to connect two locations within a cost surface (*i.e.* a friction layer).
9. There are two methods for calculating the least-cost corridors (LCCs):

   A**. Percentage of LCP value** is based on the LCP between each site. For example, if the LCP was 5.0 and a 1% LCC class cutoff was selected, LCPs with values between 5.0-5.05 would be included in that class. This formula focuses only on the LCP value and is not affected by larger cost-path values.

   B. **Percentage of Cost Path Values** is based on the range of cost path values across the entire landscape for each comparison. For example, if the least and highest cost paths were 5.0 and 505, respectively, and a 1% LCC class cutoff was selected, LCPs with values between 5.0-10.0 would be included in that class. This formula focuses on the total range of cost path values and is greatly affected by the cost-path values across the landscape.

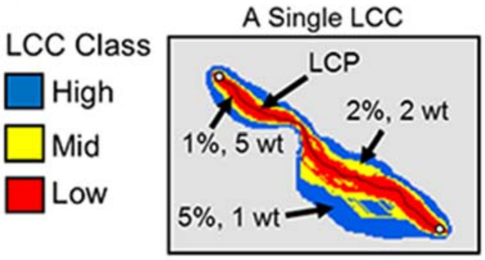

10. The *Calculate Least-Cost Corridor* tool calculates LCCs in three classes: High, Mid and Low. **High Cutoff.** Cost paths with values between this and the Mid Cutoff will be included in the High Class. The input values are integers of the percentage (e.g. 1% = 1). For overview see figure below.
11. **Mid Cutoff.** Cost paths with values between this and the Low Cutoff will be included in the Mid Class.
12. **Low Cutoff.** Cost paths with values between this and the LCP will be included in the Low Class.
13. LCC weight values. Because all LCCs are summed to create a dispersal network, weighted values are placed on each category of LCCs. The input in this box is the weight applied to the **High LCC class**.
14. Weight applied to the **Mid LCC class**.
15. Weight applied to the **Low LCC class**.

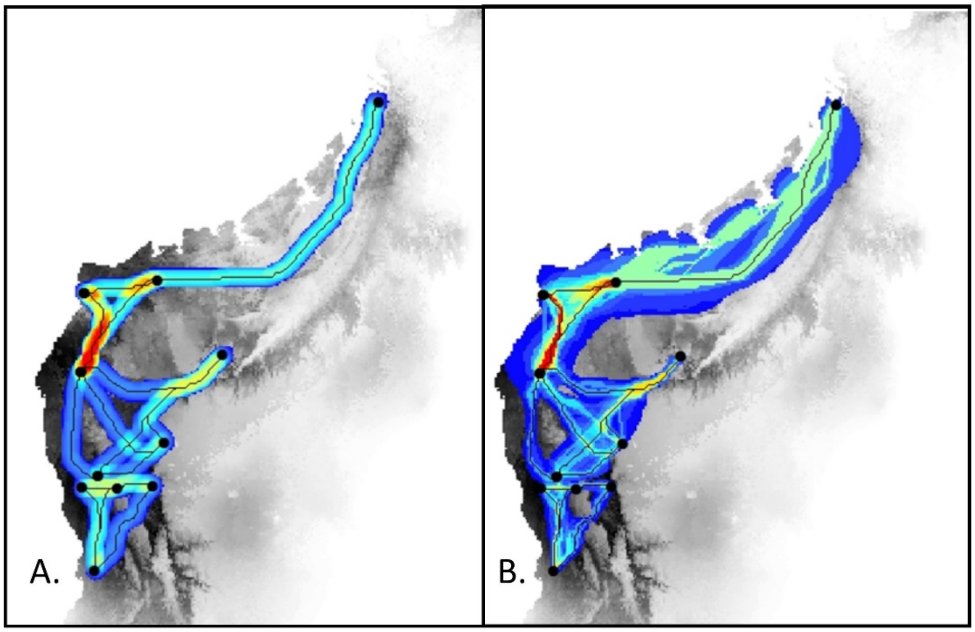

Results

Dispersal Networks. A. Least-cost paths and LCP line densities. Warmer colors depict LCP lines traversed more frequently. B. Haplotype Dispersal Networks. Warmer colors depict cost-paths traversed more frequently and represent likely connections in habitat (due to common ancestry among shared haplotypes).

Chapter 2. Create Pairwise Distance Matrix

Tool Overview

This tool will create two pair-wise distance matrices reflecting: least cost path distance and the along path cost of the least-cost path. The least-cost path distance is simply the distance of the LCP. The along path cost of the least-cost path is the total sum of the friction values that characterize the least-cost path. Each output is a n-dimensional symetrical matrix output as a CSV table. Note that some of the code used here are adapted and updated from T.R. Etherington's: Landscape Genetics toolbox. If you use this tool, cite both SDMtoolbox and the following citation:

Etherington, T.R. (2011) Python based GIS tools for landscape genetics: visualising genetic relatedness and measuring landscape connectivity, Methods in Ecology and Evolution, 2(1): 52-55.

Create Pairwise Distance Matrix

ARCGIS STEP-BY-STEP GUIDE:

1. Open a fresh ArcMap document
2. Import 'Oplurus_Haplotypes.shp' and "FrictionLayer_low_res.tif' (Location: …\example_data\Landscape_connectivity\)
3. Next double-click the 'Landscape Connectivity→Pairwise: All Sites→Create Pairwise Distance Matrix' tool
4. Continue to the tool interface instructions (following page)

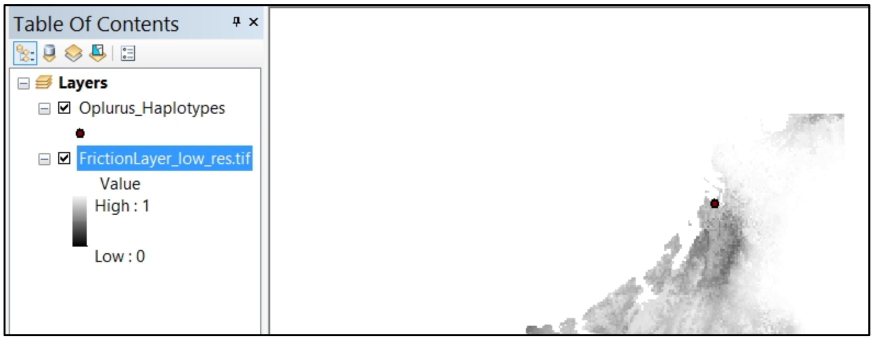

Imported data

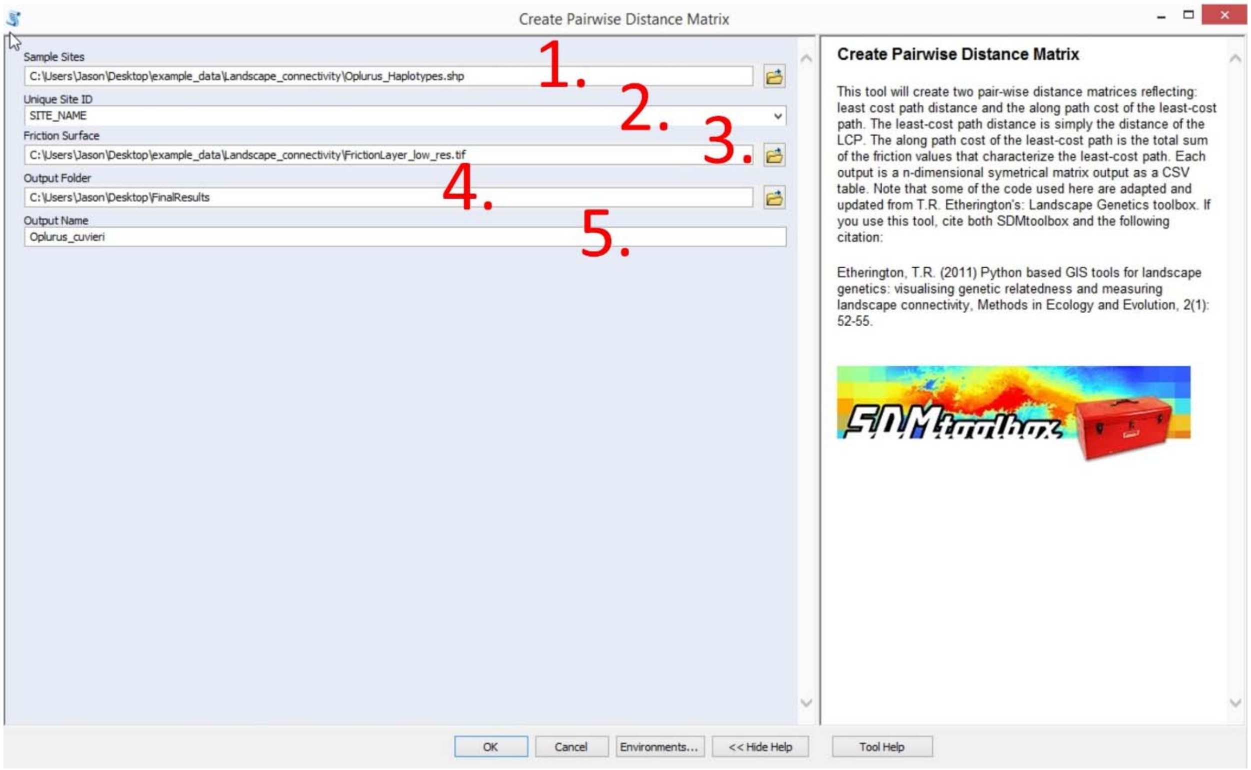

***Create Pairwise Distance Matrix* tool interface**

SDMTOOLBOX STEP-BY-STEP GUIDE:

1. Input point shapefile containing sites and haplotypes to be included in least-cost paths and corridors. This shapefile must have two columns: one depicting site names and another depicting haplotype ID.

LATITUDE	LONGITUDE	SITE_NAME	HAPLOTYPE
-16.38	45.345	1432680	C
-14.31	47.915	1569175	A
-16.525	44.49	1660120	A
-16.525	44.49	1660120	C
-16.525	44.49	1660120	F
-19.75	44.6167	APR1031	B
-18.7967	44.8817	HER2558	B
-18.6533	44.6583	HER2725	D
-18.6533	44.6583	HER2725	E

Required geospatial and haplotype data for analysis

Tip. If you need to convert a table to a shapefile for use here. You need two extra columns (latitude and longitude, see table above). Then use the tool 'CSV & Shapefile Tools → 1. CSV to Shapefile'
-Avoid non-alphanumeric characters in both names (e.g., avoid: * : \ / < > | " ? [ ] ; = + & £ $, etc.).

2. Table field (part of shapefile) depicting site ID. Note this cannot be only a number. If a number recode as alphanumeric ID (e.g. change '9' to '9a')
3. A friction Layer is a raster that depicts the ease of dispersal from each locality through the landscape. In this analysis, the friction layer depicts the output extent and spatial resolution of analysis. If you want a larger extent or a lower spatial resolution, increase both in your friction layer. However, the larger both are—the longer the analysis will take.

Creating a friction layer. One prevalent way to create a friction layer that doesn't suffer from applying weights to habitat types (often associated with contributing to biases in results) is the use of SDMs. An SDMtoolbox tool (location below) will invert a SDM for use as a friction surface. Using this method, areas of high suitability will be converted to areas of low dispersal cost.

Tool Path: SDM Tools →1. Universal Tools →Create Friction Layer →Invert SDM

4. This should be a new empty folder. If not empty this can cause the analysis to fail, particularly if temporary files from a previous analysis were not properly removed (e.g. this can happen if another SDMtoolbox analysis is terminated early).
5. Input desired output name. Note when this category is highlighted the Help Box displays the text in the box to the right. I input 'Oplurus_cuvieri', this means that the output files will be named: 'Oplurus_cuvieri_LCP_cost.csv', 'Oplurus_cuvieri_ LCP_distance.csv', 'Oplurus_cuvieri.shp' Select output folder location.

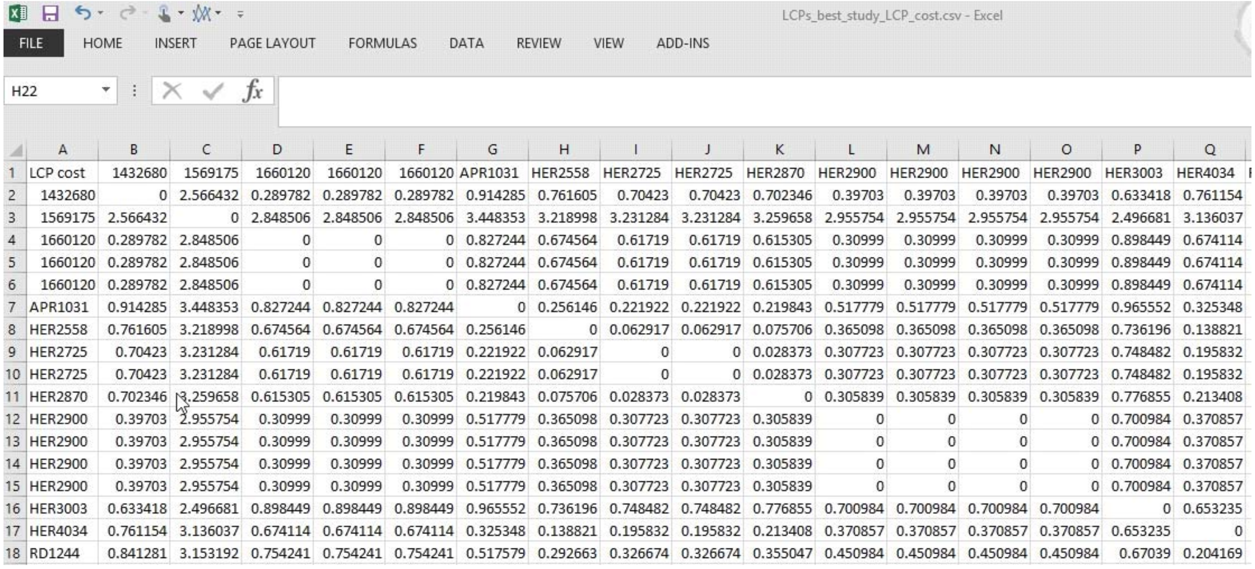

LCPs_best_study_LCP_cost.csv - Excel

FILE HOME INSERT PAGE LAYOUT FORMULAS DATA REVIEW VIEW ADD-INS

H22

	A	B	C	D	E	F	G	H	I	J	K	L	M	N	O	P	Q
1	LCP cost	1432680	1569175	1660120	1660120	1660120	APR1031	HER2558	HER2725	HER2725	HER2870	HER2900	HER2900	HER2900	HER2900	HER3003	HER4034
2	1432680	0	2.566432	0.289782	0.289782	0.289782	0.914285	0.761605	0.70423	0.70423	0.702346	0.39703	0.39703	0.39703	0.39703	0.633418	0.761154
3	1569175	2.566432	0	2.848506	2.848506	2.848506	3.448353	3.218998	3.231284	3.231284	3.259658	2.955754	2.955754	2.955754	2.955754	2.496681	3.136037
4	1660120	0.289782	2.848506	0	0	0	0.827244	0.674564	0.61719	0.61719	0.615305	0.30999	0.30999	0.30999	0.30999	0.898449	0.674114
5	1660120	0.289782	2.848506	0	0	0	0.827244	0.674564	0.61719	0.61719	0.615305	0.30999	0.30999	0.30999	0.30999	0.898449	0.674114
6	1660120	0.289782	2.848506	0	0	0	0.827244	0.674564	0.61719	0.61719	0.615305	0.30999	0.30999	0.30999	0.30999	0.898449	0.674114
7	APR1031	0.914285	3.448353	0.827244	0.827244	0.827244	0	0.256146	0.221922	0.221922	0.219843	0.517779	0.517779	0.517779	0.517779	0.965552	0.325348
8	HER2558	0.761605	3.218998	0.674564	0.674564	0.674564	0.256146	0	0.062917	0.062917	0.075706	0.365098	0.365098	0.365098	0.365098	0.736196	0.138821
9	HER2725	0.70423	3.231284	0.61719	0.61719	0.61719	0.221922	0.062917	0	0	0.028373	0.307723	0.307723	0.307723	0.307723	0.748482	0.195832
10	HER2725	0.70423	3.231284	0.61719	0.61719	0.61719	0.221922	0.062917	0	0	0.028373	0.307723	0.307723	0.307723	0.307723	0.748482	0.195832
11	HER2870	0.702346	[illegible].259658	0.615305	0.615305	0.615305	0.219843	0.075706	0.028373	0.028373	0	0.305839	0.305839	0.305839	0.305839	0.776855	0.213408
12	HER2900	0.39703	2.955754	0.30999	0.30999	0.30999	0.517779	0.365098	0.307723	0.307723	0.305839	0	0	0	0	0.700984	0.370857
13	HER2900	0.39703	2.955754	0.30999	0.30999	0.30999	0.517779	0.365098	0.307723	0.307723	0.305839	0	0	0	0	0.700984	0.370857
14	HER2900	0.39703	2.955754	0.30999	0.30999	0.30999	0.517779	0.365098	0.307723	0.307723	0.305839	0	0	0	0	0.700984	0.370857
15	HER2900	0.39703	2.955754	0.30999	0.30999	0.30999	0.517779	0.365098	0.307723	0.307723	0.305839	0	0	0	0	0.700984	0.370857
16	HER3003	0.633418	2.496681	0.898449	0.898449	0.898449	0.965552	0.736196	0.748482	0.748482	0.776855	0.700984	0.700984	0.700984	0.700984	0	0.653235
17	HER4034	0.761154	3.136037	0.674114	0.674114	0.674114	0.325348	0.138821	0.195832	0.195832	0.213408	0.370857	0.370857	0.370857	0.370857	0.653235	0
18	RD1244	0.841281	3.153192	0.754241	0.754241	0.754241	0.517579	0.292663	0.326674	0.326674	0.355047	0.450984	0.450984	0.450984	0.450984	0.67039	0.204169

Results

Pairwise distance matrix: A. path cost and B. path distance (not picture)

Chapter 3. Species Distribution Modeling Analyses

- *Correcting Latitudinal Background Selection Bias*
  - Solution 1: Bias Files
  - Solution 2: Project Input Data to Equal Areas Projection
- *Distribution Changes Between Binary SDMs*
  - Centroid Changes (Lines)
  - Distribution Changes Between Binary SDMs
- *Overprediction Correction: Clip Models by Buffered Minimum Convex Polygons*
  - Binary Models (folder)
  - Continuous Models (folder)
- *Background Selection via Bias Files*
  - Sample by Buffered MCPs
  - Sample by Distance from Observed Localities
- *Create Friction Layer: Invert SDM*

Correcting Latitudinal Background Selection Bias

TOOL OVERVIEW

If you are using data that are in a geographic coordinate system (such as, degrees minutes seconds or decimal degrees) for MaxEnt analyses (and most other background and pseudoabsence based SDM methods)--- then ***you*** are biasing your selection of background points (or pseudoabsence points) and unique observed localities toward the poles. The level of bias depends on the breadth of latitudes your analyses cover. The reason for this is due to the area occupied by these units decreases latitudinally (as values increase, see Table 1), with areas largest at equator and smallest at poles. This inequality results from convergence of the meridians (lines of longitude) towards the poles.

Table 1. Area Covered By a Square Decimal Degree at Different Latitudes

$Degrees^2$	N/S or E/W at equator	E/W at 23N/S	E/W at 45N/S	E/W at 67N/S
1.0	12392 km^2	11407 km^2	8762 km^2	4842 km^2
% Area of Equator	100%	92.0%	70.7%	39.0%
Relative Sampling Bias (1= none, 2= 2x sampling bias)	1	1.09	1.41	2.85

There are two solutions to this issue. The first solution corrects the bias sampling problem by correcting how background values and unique occurrence localities are selected. The second solution fixes the problem by projecting all the data into an equal-areas projection (EAP). The latter is the preferred method, however for many modelers, this requires

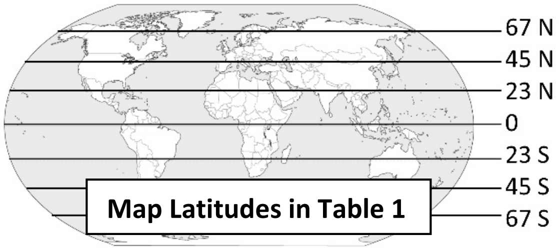

Map Latitudes in Table 1

considerable effort and can be confusing due to issues associated with selecting the best EAP. The SDM toolbox facilitates both solutions.

The first set of tools clips a Coordinate Bias File (downloadable from www.sdmtoobox.org) to the size of your MaxEnt analysis and then calculates a Bias File for Coordinate Data (BFCD) for that area. The Coordinate Bias File accounts for background sampling biases associated with latitudinal changes in the area encompassed by decimal degree units. This tool converts the Coordinate Bias File to the proportion of area occupied (relative to the largest value present in your analyses) to be used as a bias file in MaxEnt. A value of 2 in the output file depicts a 50% reduction in cell area (vs. cell values of 1). Thus, in absence of a BFCD, the probability of creating a background in the cell with a value of 2 is twice as high as a cell with a value of 1. The BFCD allows equal sampling of background points throughout the landscape in geographic projections.

Info on the downloaded Coordinate Bias File: Units equal percent of area relative to size at equator (where square decimal degrees constitute the largest area). Raw percentage in downloaded file was multiplied by 10 (reducing file size by saving it as an integer file). Thus values 0 –1000 reflect 0.0 to 100.0 % of area occupied by a decimal degree (or any subunit of them) at the equator.

The second set of tools facilitate transforming climate and species occurrence data to EAP for use in MaxEnt (formatting output for direct use).

Solution 1

Quick and Easy Solution. Creating a Bias file in MaxEnt to account for biases

Tool: 1. Bias File for Coordinate Data (BFCD) in MaxEnt

ARCGIS STEP-BY-STEP GUIDE:

1. Open a fresh ArcMap document
2. Import ‘Uruloke_eowynae.shp’ and ‘bio_1.asc’
   (Location: …\example_data\sdm_analyses\correcting_lat_BPs\soultion1\)
3. Download and unzip a the 30-arc second Coordinate Bias File from www.sdmtoolbox.org
4. Next double-click the ‘SDM Tools →2. MaxEnt Tools → Correcting Latitudinal Background Selection→Solution 1: Bias Files→ 1. Bias File for Coordinate Data (BFCD)’ tool
5. Continue to tool interface instructions (following page)

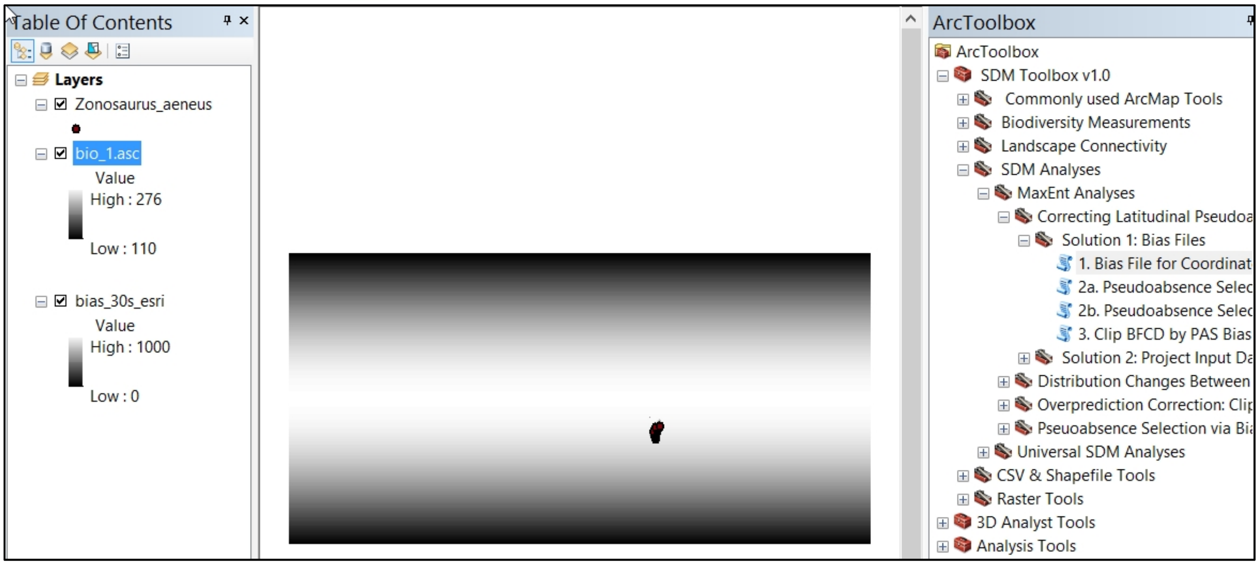

Imported Data

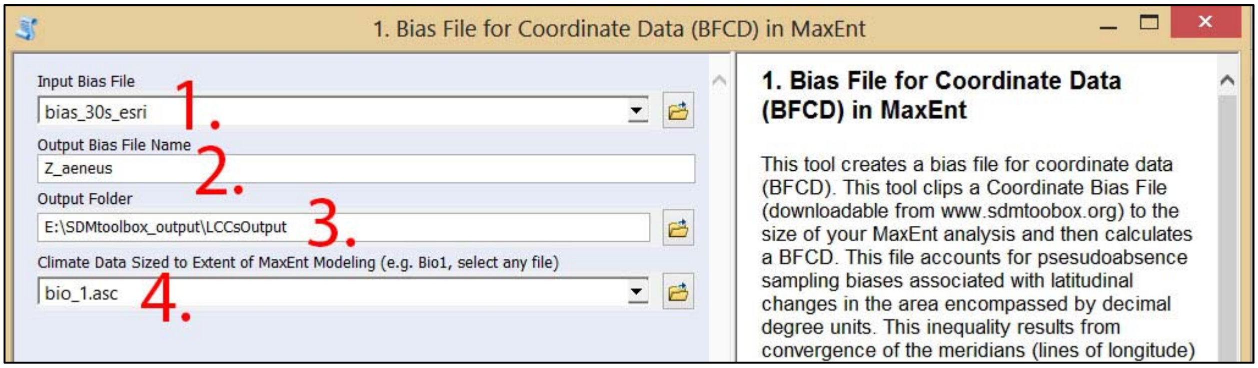

***1. Bias File for Coordinate Data (BFCD) in MaxEnt* tool interface**

SDMtoolbox Step-by-Step Guide:

1. Download and unzip Coordinate Bias File from www.sdmtoolbox.org (this must match the resolution of your climate data)
2. Output name. Here I used 'U_eowynae'. Note: '_Coord_Bias_file_for_MaxEnt' will be appended to the end of the output name.
3. Select output folder location. This should be a new empty folder. If not empty this can cause the analysis to fail, particularly if temporary files from a previous analysis were not properly removed (e.g. this can happen if another SDMtoolbox analysis is terminated early).
4. Climate data sized to extent of MaxEnt Modeling. Here use the imported 'Bio_1.asc' layer. Select one of your climate files sized to your modeling extent (e.g. Bio1.asc). This file will be used to match the bias file to proper extent and resolution (no change will be made to this file).

Correcting Latitudinal Background Selection Bias: Solution 1

Tool: 2a. Background Selection: Sample by Buffered MCP

ARCGIS STEP-BY-STEP GUIDE:

1. This analysis is continued from previous (the same input data are required)
2. Double-click the 'SDM Tools →2. MaxEnt Tools → Correcting Latitudinal Background Selection→Solution 1: Bias Files→ 2a. Background Points: Sample by Buffered MCP'
3. Continue to tool interface instructions (below)

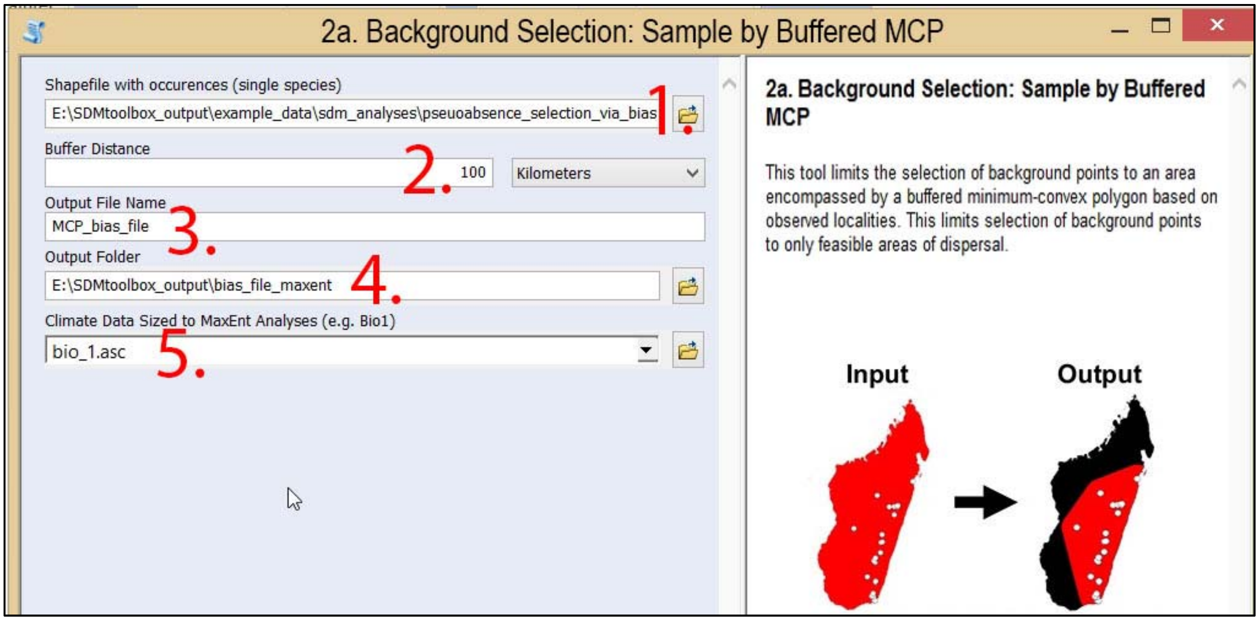

***2a. Background Selection: Sample by Buffered MCP* tool interface**

SDMTOOLBOX STEP-BY-STEP GUIDE:

1. Shapefile of species occurrence.

> **Tip.** If you need to convert a table to a shapefile for use here. You need two extra columns with latitude and longitude. Then use the tool 'CSV & Shapefile Tools → 1. CSV to Shapefile' -Avoid non-alphanumeric characters in both names (e.g., avoid: * : \ / < > | " ? [ ] ; = + & £ $, etc.).

2. The distance outside of minimum-convex-polygon included in background point selection
3. Output name. Note: '_BP_Bias_file_for_MaxEnt' will be appended to the end of the output name.
4. Select output folder location. This should be a new empty folder. If not empty this can cause the analysis to fail, particularly if temporary files from a previous analysis were not properly removed (e.g. this can happen if another SDMtoolbox analysis is terminated early).
5. Climate data sized to extent of MaxEnt Modeling. Here use the imported 'Bio_1.asc' layer. Select one of your climate files sized to your modeling extent (e.g. Bio1.asc). This file will be used to match the bias file to proper extent and resolution (no change will be made to this file).

Correcting Latitudinal Background Selection Bias: Solution 1

Tool: 3. Clip BFCD by Background Selection Bias File

ArcGIS Step-by-Step Guide:

1. This analysis is continued from previous (outputs from both steps are required)
2. Next double-click the ‘SDM Tools →2. MaxEnt Tools → Correcting Latitudinal Background Selection→Solution 1: Bias Files→ 3. Clip BFCD by BS Bias File’
3. Continue to tool interface instructions (below)

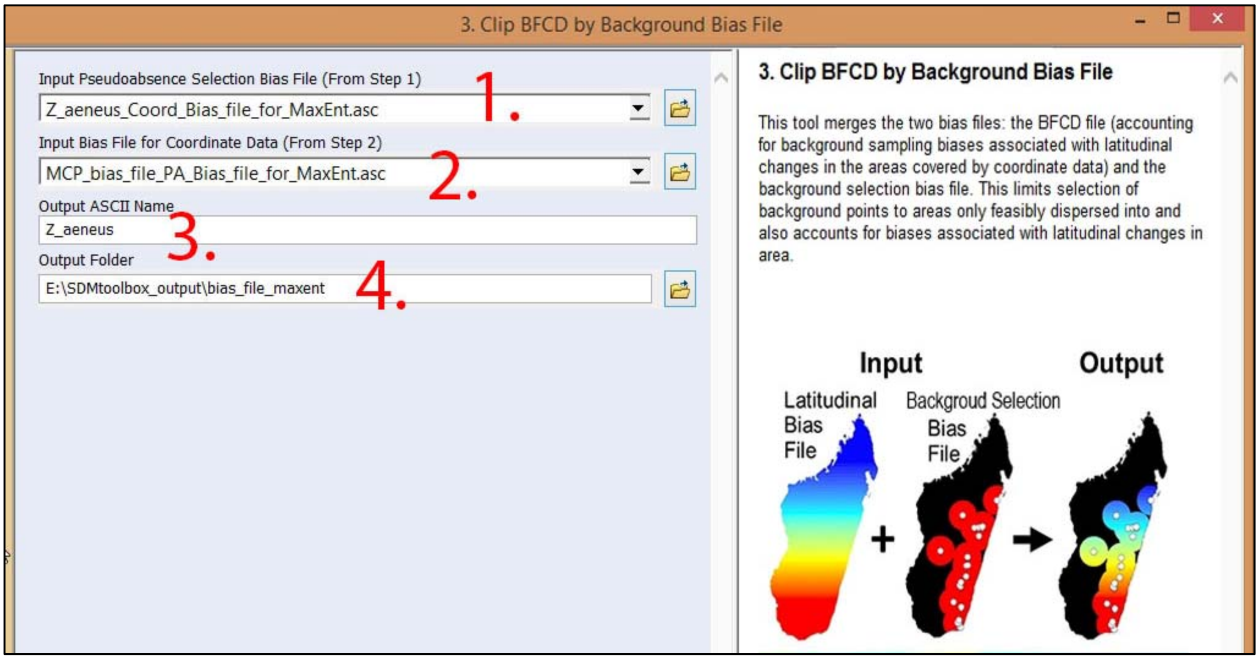

3. Clip BFCD by Background Selection Bias File **tool interface**

SDMtoolbox Step-by-Step Guide:

1. Output from tool: 1. Bias File for Coordinate Data (BFCD) in MaxEnt
2. Output from tool:
   2a. Background Selection: Sample by Buffered MCP Tool Interface
   2b. Background Selection: Sample by Distance from Pts.
3. Output name. Note: ‘_Both_Bias_file_for_MaxEnt’ will be appended to the end of the output name.
4. Select output folder location. This should be a new empty folder. If not empty this can cause the analysis to fail, particularly if temporary files from a previous analysis were not properly removed (e.g. this can happen if another SDMtoolbox analysis is terminated early).

Solution 2

Best practice: Projecting all data into an equal-areas projection

Tool: 1. CSV to EAP. MaxEnt format output (runs both 1a and 1b)

ArcGIS Step-by-Step Guide:

1. Open a fresh ArcMap document.
2. Next double-click the 'SDM Tools →2. MaxEnt Tools → Correcting Latitudinal Background Selection→ Solution 2: Project Input Data to Equal-Area Projection (EAP)→ 1. CSV to EAP.' MaxEnt format output (runs both 1a and 1b)' tool
3. Continue to tool interface instructions (below)

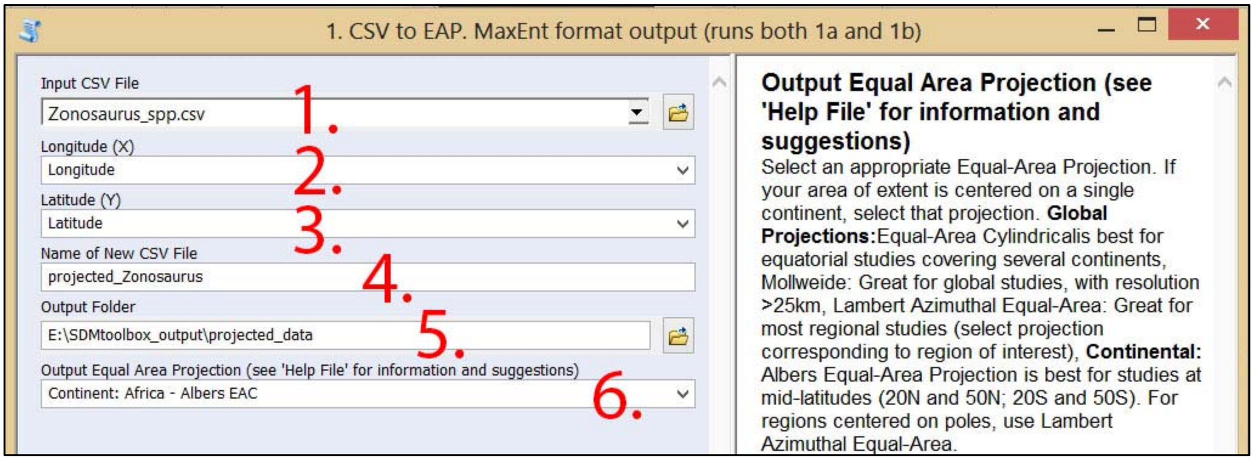

1. ***CSV to EAP. MaxEnt format output (runs both 1a and 1b)* tool interface**

SDMtoolbox Step-by-Step Guide:

1. Import 'Uruloke_spp.csv'
   (Location: …\example_data\sdm_analyses\correcting_lat_BPs\soultion2\)
   This file should match the format required for MaxEnt (see table layout below, saved as '.csv' file). Note this tool will only accept latitude written as: 'LATITUDE', 'latitude', 'Latitude' and longitude written as: 'LONGITUDE', 'latitude', 'Latitude'.
2. Select field corresponding to latitude.
3. Select field corresponding to longitude.
4. Output name for projected CSV file.
5. Select output folder location. This should be a new empty folder. If not empty, this can cause the analysis to fail, particularly if temporary files from a previous analysis were not properly removed (e.g. this can happen if another SDMtoolbox analysis is terminated early).
6. Select an appropriate Equal-Area Projection. If your area of extent is centered on a single continent, select that projection.

species	Longitude	Latitude
Zonosaurus_aeneus	46.917	-24.983
Zonosaurus_aeneus	46.917	-24.983
Zonosaurus_aeneus	46.98389	-24.97944
Zonosaurus_aeneus	46.98389	-24.97944
Zonosaurus_aeneus	46.86667	-24.75

Table 3. MaxEnt Input Species Data Format: three columns with species, longitude, latitude.

Help with Equal Area Projections:

Global Projections: Equal-Area Cylindrical is best for equatorial studies covering several continents; Mollweide is great for global studies with resolution >25km; Lambert Azimuthal Equal-Area is great for most regional studies.

Continental: Albers Equal-Area Projection is best for studies at mid-latitudes (20N and 50N; 20S and 50S). For regions centered on poles, use Lambert Azimuthal Equal-Area.

Reminder. Use the same projection for all rasters and CSVs in the study

Correcting Latitudinal Background Selection Bias: Solution 2

Tool: 2. Project Climate Data (Raster) to Equal-Area Projection (Folder)

ArcGIS Step-by-Step Guide:

1. Double-click the 'SDM Tools →2. MaxEnt Tools→ Correcting Latitudinal Background Selection→ Solution 2: Project Input Data to Equal-Area Projection (EAP)→ 2. Project Climate Data (Raster) to Equal-Area Projection (Folder)' tool
2. Continue to tool interface instructions (below)

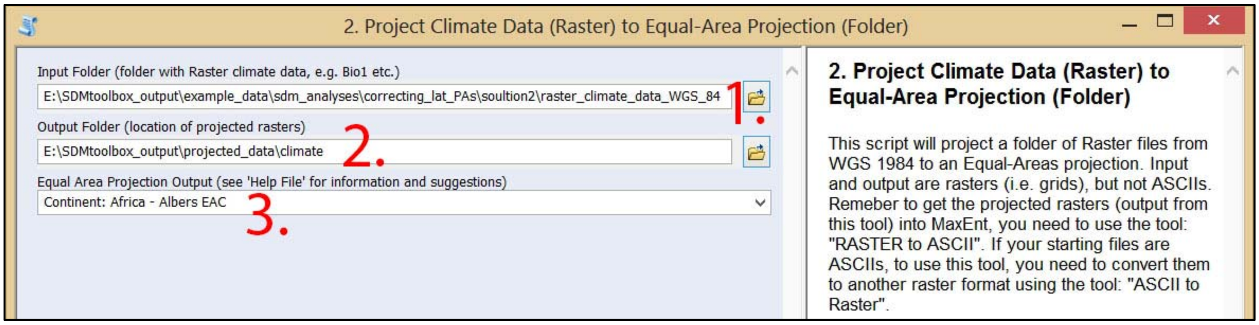

***2. Project Climate Data (Raster) to Equal-Area Projection (Folder)* tool interface**

SDMtoolbox Step-by-Step Guide:

1. Input data need to be a non-ASCII raster files and all files should be in a single folder. If ASCII files, first use the '2b. ASCII to Raster (folder)' tool (part of the 'Basic Tools → Raster Tools' group).
2. Select output folder location. This should be a new empty folder. If not empty this can cause the analysis to fail, particularly if temporary files from a previous analysis were not properly removed (e.g. this can happen if another SDMtoolbox analysis is terminated early).
3. Select an appropriate Equal-Area Projection. Use the same projection selected for the CSV species file (pg. 26 step 6).
4. Run tool. Then use the '2a. Raster to ASCII (folder)' tool, part of the 'Raster Tools' group, to convert newly projected rasters to ASCII files (input format for use in MaxEnt).

TOOL OVERVIEW

Distribution Changes Between Binary SDMs

A common use of species distribution models is to predict distributional changes due to climate change. Here I created two tools that help summarize distributional changes. The first tool calculates the distributional changes between two binary SDMs (e.g. current and future SDMs). Output is a table depicting predicted contraction, expansion, and areas of no change in the species' distribution. A second tool also calculates the distributional changes between two binary SDMs (e.g. current and future SDMs), however this analysis is focused on summarizing the core distributional shifts of the ranges of many species. This analysis reduces each species' distribution to a single central point (known as a centroid) and creates a vector file depicting magnitude and direction of predicted change through time.

Distribution Changes Between Binary SDMs

Tool: Centroid Changes (Lines)

ARCGIS STEP-BY-STEP GUIDE:

1. Open a fresh ArcMap document.
2. Double-click the 'SDM Tools →2. MaxEnt Tools → Distribution Changes Between Binary SDMs →Centroid Changes' tool
3. Continue to tool interface instructions (following page)

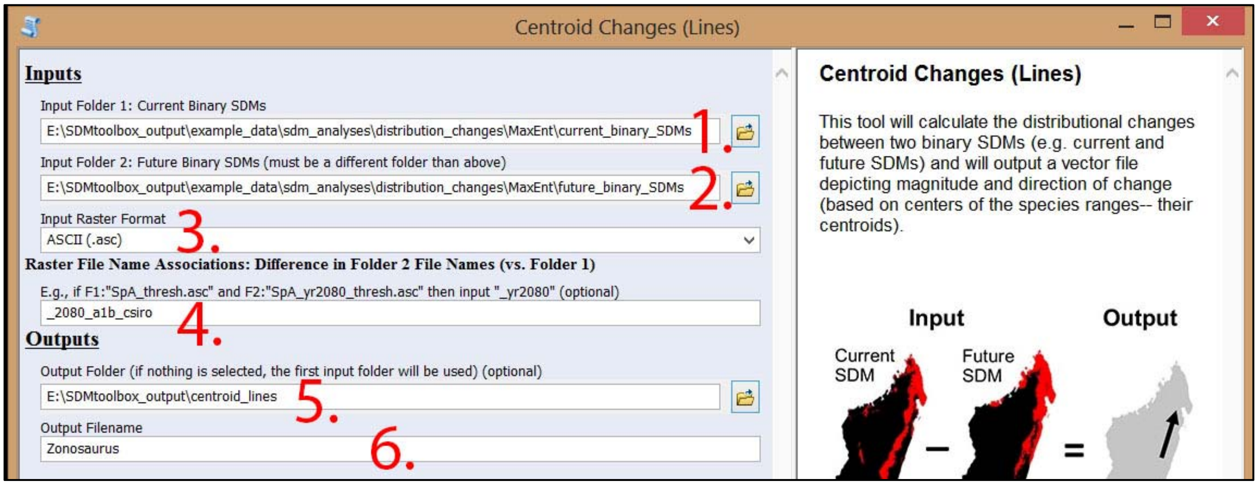

Centroid Changes (Lines) **tool interface**

SDMtoolbox Step-by-Step Guide:

1. Input Folder 1: The folder containing current binary SDMs.

   (Location:…/example_data/sdm_analyses/distribtutionchanges/MaxEnt/current_binary_SDMs)

2. Input Folder 2: The folder containing future binary SDMs.

   (Location: …/example_data/sdm_analyses/distribtutionchanges/MaxEnt/future_binary_SDMs)

3. Input raster type. Here the example data are 'ASCII (.asc)' raster files.
4. Raster file name associations. Here input '_2080_a1b_csiro'. Because MaxEnt names the future and current SDMs differently, you need to input the difference in file names. For example, if the current SDM for a species A is named 'SpA_threshold.asc' and the future is named 'SpA_yr2080_threshold.asc' then input '_yr2080'. Note: The suffix '_threshold' will automatically be accounted for by the tool. If your SDMs do not have this, then you must use the generic 'Centroid Changes (Lines)' tool [located at: SDM Tools→ 1. Universal Tools→ Centroid Changes (Lines)], which has more flexibility in input file names.
5. Select output folder location. This should be a new empty folder. If not empty this can cause the analysis to fail, particularly if temporary files from a previous analysis were not properly removed (e.g. this can happen if another SDMtoolbox analysis is terminated early).
6. Output name. Note: '_centroid_change' and '_cen_chg_density' will be appended to the end of the output name.

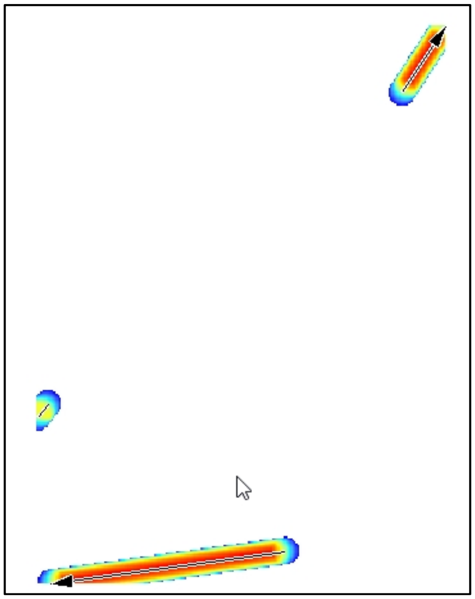

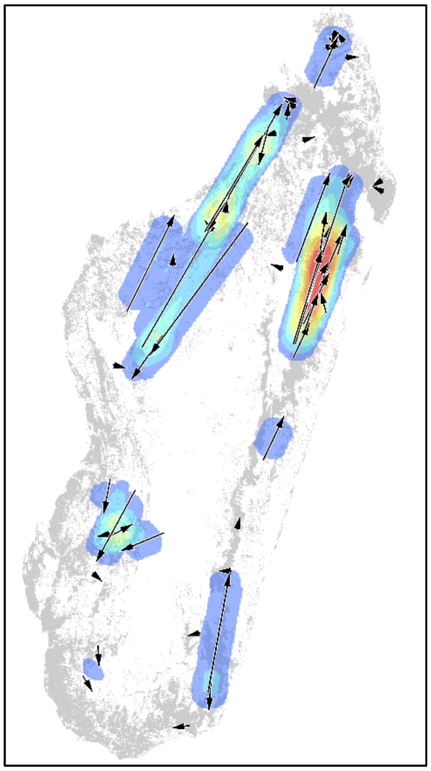

Results: Above. Output from example data. Right. Example of a multispecies comparison to demonstrate power of such analysis. In the northeast of Madagascar many species will respond similarly to climate change

Distribution Changes Between Binary SDMs

Tool: Distribution Changes Between Binary SDMs

ARCGIS STEP-BY-STEP GUIDE:

1. Open a fresh ArcMap document.
2. Double-click the 'SDM Tools →2. MaxEnt Tools → Distribution Changes Between Binary SDMs → Distribution Changes Between Binary SDMs' tool
3. Continue to tool interface instructions (following page)

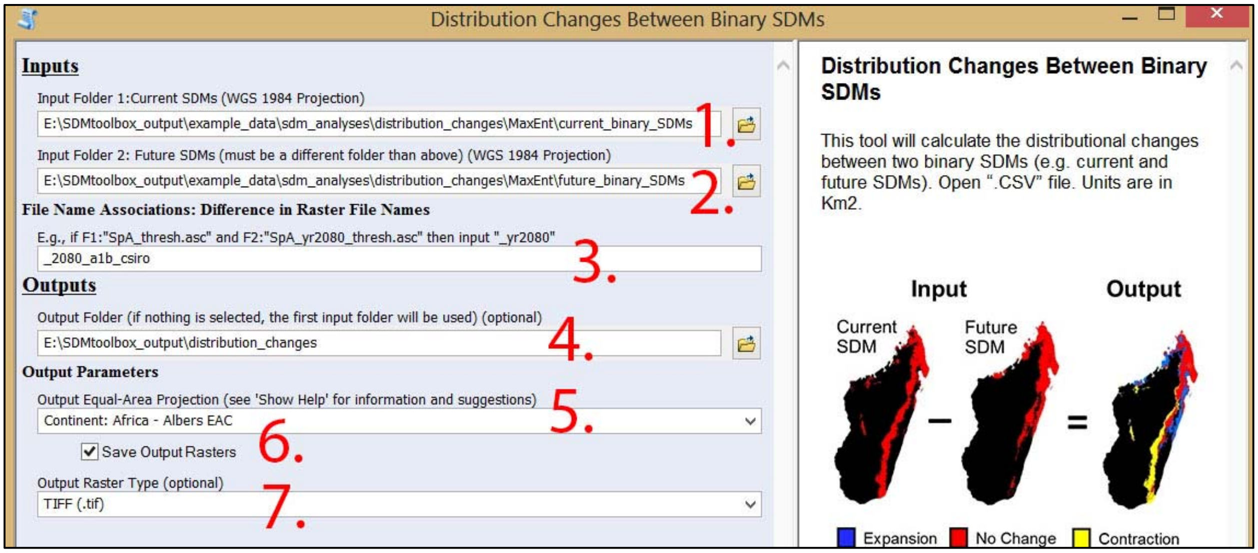

Distribution Changes Between Binary SDMs **tool interface**

SDMTOOLBOX STEP-BY-STEP GUIDE:

1. Input Folder 1: The folder containing current binary SDMs.

   (Location: ...\example_data\sdm_analyses\distribtutionchanges\MaxEnt\current_binary_SDMs)

2. Input Folder 2: The folder containing future binary SDMs.

   (Location: ...\example_data\sdm_analyses\distribtutionchanges\MaxEnt\future_binary_SDMs)

3. Raster file name associations. Here input '_2080_a1b_csiro'. Because MaxEnt names the future and current SDMs differently, you need to input the difference in file names. For example, if the current SDM for species A is named 'SpA_threshold.asc' and the future is named 'SpA_yr2080_threshold.asc' then input '_yr2080'. Note: The suffix '_thresholded' will automatically be accounted for. If your SDMs do not have this then you must use the universal 'Distribution Changes Between Binary SDMs' tool. [located at: SDM Tools→ 1. Universal Tools→ Distribution Changes Between Binary SDMs], which has more flexibility in input file names.
4. Select output folder location. This should be a new empty folder. If not empty this can cause the analysis to fail, particularly if temporary files from a previous analysis were not properly removed (e.g. this can happen if another SDMtoolbox analysis is terminated early).

5. Select an appropriate Equal-Area Projection. If your area of extent is centered on a single continent, select that projection.

> **Help with Equal Area Projections:**
>
> **Global Projections:** Equal-Area Cylindrical is best for equatorial studies covering several continents; Mollweide is great for global studies with resolution >25km; Lambert Azimuthal Equal-Area is great for most regional studies.
>
> **Continental:** Albers Equal-Area Projection is best for studies at mid-latitudes (20N and 50N; 20S and 50S). For regions centered on poles, use Lambert Azimuthal Equal-Area.
>
> Reminder. Use the same projection for all rasters and CSVs in the study

6. Check to save the raster files of results (see below)
7. Output file type. Here I selected 'Tiff (.tif)' format.

> **Tip.** I prefer TIFF files as output format because they allow for longer file names (vs. ESRI grid files that are limited to 13 characters) and don't have too many raw parts to each file. There is, however, a slight reduction in performance (vs. ESRI grid files), thus, if processing thousands of rasters this should be taken into consideration.

Results

SDM Tools: Distribution Changes of zonosaurus_madagascariensis_changes

Output Key: -1 = range expansion; 0= no occupancy (absence in both); 1 = no change (presence in both); 2 = range contraction

Value	Area (Km2)
-1	525.2446
0	458408.8
1	82113.24
2	47615.69

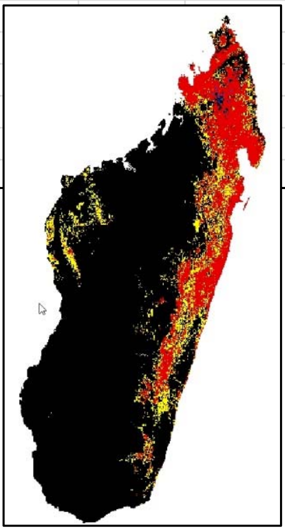

Results: CSV file of area changes and to right, a raster of results (optional).

TOOL OVERVIEW

Overprediction Correction: Clip Models by Buffered Minimum Convex Polygons

To limit over-prediction of SDMs, a problem common with modeling species distributions, two tools were created that clip SDMs by a buffered minimum convex polygon (MCP) generated from the input point data of each species following the approach of Kremen *et al.* (2008). This method produces models that represent suitable habitat within an area of known occurrence (based on a buffered MCP), excluding suitable habitat greatly outside of observed range and unsuitable habitat through the landscape.

Kremen, C., A. Cameron, A. Moilanen, S. J. Phillips, C. D. Thomas, H. Beentje, J. Dransfield, B. L. Fisher, F. Glaw, T. C. Good, G. J. Harper, R. J. Hijmans, D. C. Lees, E. Louis Jr., R. A. Nussbaum, C. J. Raxworthy, A. Razafimpahanana, G. E. Schatz, M. Vences, D. R. Vieites & M. L. Zjhra (2008): Aligning conservation priorities across taxa in Madagascar with high-resolution planning tools. – *Science* **320**: 222-226.

Overprediction Correction: Clip Models by Buffered Minimum Convex Polygons

Tool: Binary Models (folder)

ARCGIS STEP-BY-STEP GUIDE:

1. Open a fresh ArcMap document.
2. Import 'points.shp'
   (Location: …\example_data\sdm_analyses\overprediction_correction\)
3. Import 'bio_1.asc'
   (Location: …\example_data\sdm_analyses\overprediction_correction\climate_data)
4. Double-click the 'SDM Tools →2. MaxEnt Tools→ Overprediction Correction: Clip Models by Buffered MCPs →Binary Models (Folder)' tool
5. Continue to tool interface instructions (following page)

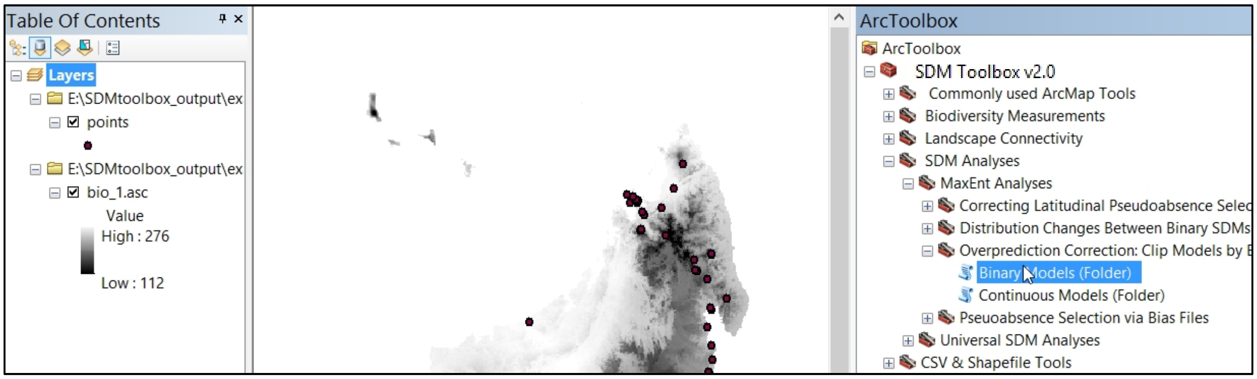

Imported Data

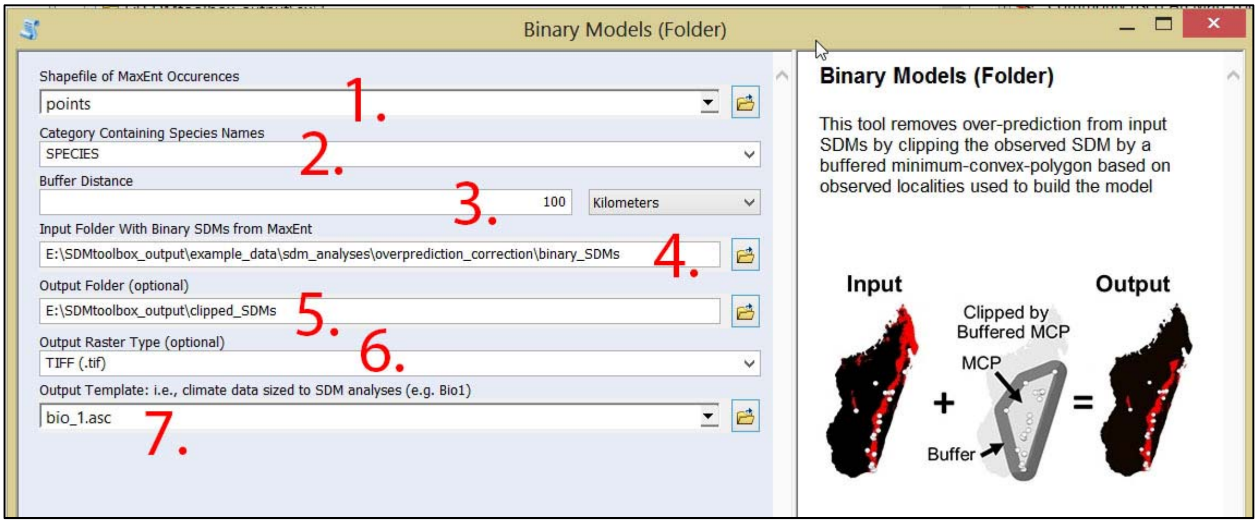

Binary Models (folder) **tool interface**

SDMtoolbox Step-by-Step Guide:

1. A point shapefile of species occurrences corresponding to input SDMs. Note: species name in shapefile must perfectly match input occurrence data species names.
2. Field indicating species names, here: 'SPECIES'
3. Distance to buffer minimum-convex polygons. Here try: 100km.
4. The folder containing binary SDMs to be clipped.

   (Location: …\example_data\sdm_analyses\overprediction_correction\binary_SDMs)

5. Select output folder location. This should be a new empty folder. If not empty this can cause the analysis to fail, particularly if temporary files from a previous analysis were not properly removed (e.g. this can happen if another SDMtoolbox analysis is terminated early).
6. Output file type. Here I selected 'Tiff (.tif)' format.
7. Climate data sized to extent of MaxEnt Modeling. Here use the imported 'Bio_1.asc' layer. Select one of your climate files sized to your modeling extent (e.g. Bio1.asc). This file will be used to match the bias file to proper extent and resolution (no change will be made to this file).

Results

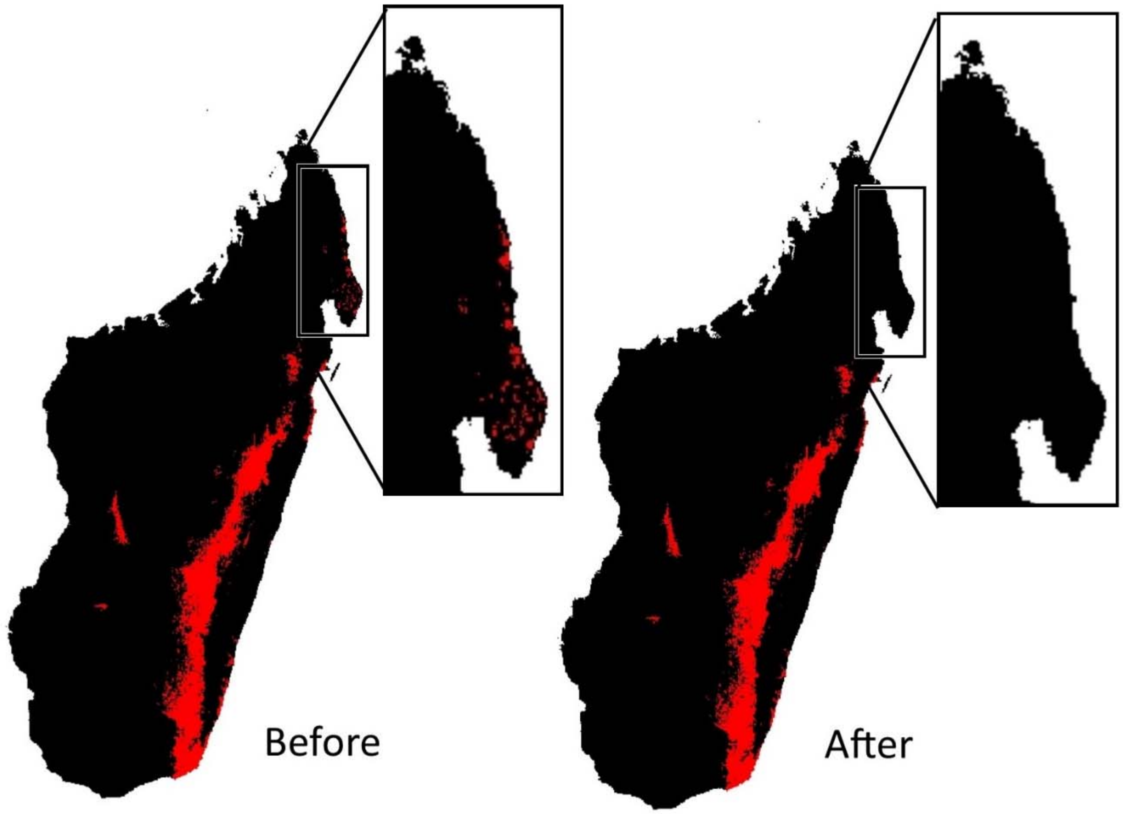

Overprediction Correction: Clip Models by Buffered Minimum Convex Polygons

Tool: Continuous Models (folder)

ArcGIS Step-by-Step Guide:

1. Open a fresh ArcMap document
2. Import 'points.shp'
   (Location: …\example_data\sdm_analyses\overprediction_correction\)
3. Import 'bio_1.asc'
   (Location: …\example_data\sdm_analyses\overprediction_correction\climate_data)
4. Double-click the 'SDM Tools →2. MaxEnt Tools → Overprediction Correction: Clip Models by Buffered MCPs →Continuous Models (Folder)' tool
5. Continue to tool interface instructions

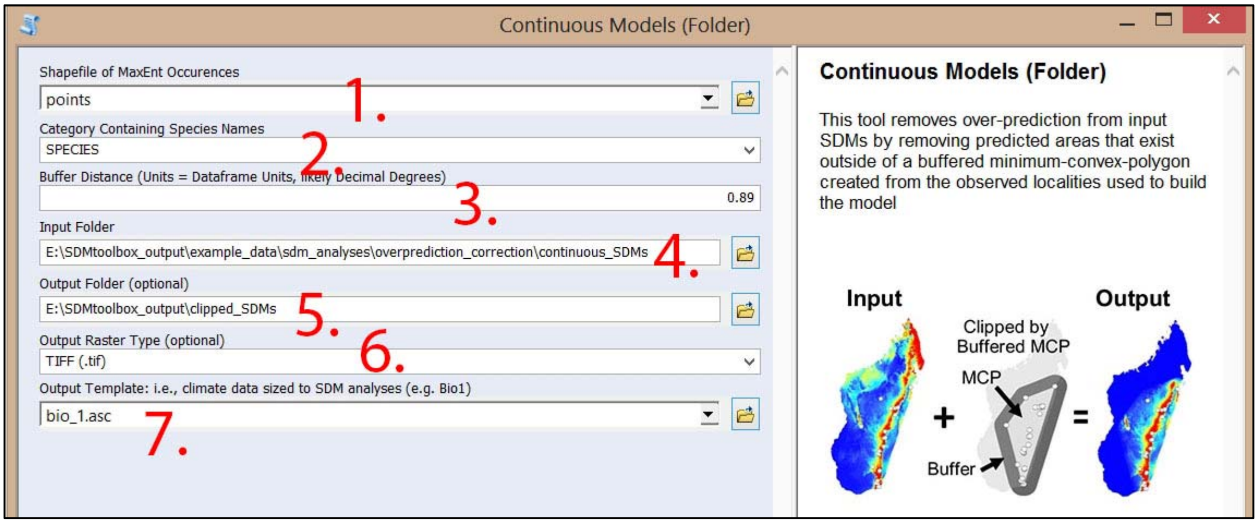

Continuous Models (folder) **tool interface**

SDMtoolbox Step-by-Step Guide:

1. A point shapefile of species occurrences corresponding to input SDMs. Note. Species name in shapefile must perfectly match input occurrence data species names.
2. Field indicating species names. Here 'SPECIES'
3. Distance to buffer minimum-convex polygons. Here try 0.89 (which is ca. 100km at equator)
4. The folder containing binary SDMs to be clipped.

   (Location: …\example_data\sdm_analyses\overprediction_correction\binary_SDMs)

5. Select output folder location. This should be a new empty folder. If not empty this can cause the analysis to fail, particularly if temporary files from a previous analysis were not properly removed (e.g. this can happen if another SDMtoolbox analysis is terminated early).
6. Output file type. Here I selected 'Tiff (.tif)' format.

7. Climate data sized to extent of MaxEnt Modeling. Here use the imported 'Bio_1.asc' layer. Select one of your climate files sized to your modeling extent (e.g. Bio1.asc). This file will be used to match the bias file to proper extent and resolution (no change will be made to this file).

Results

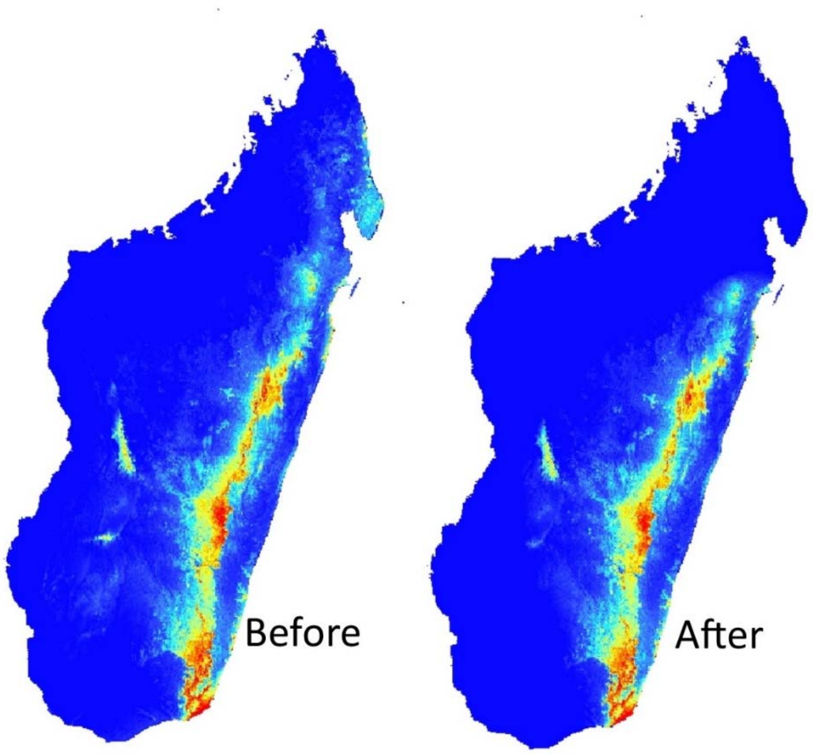

TOOL OVERVIEW

Background Selection via Bias Files

A subset of python scripts create bias files used to fine-tune background and occurrence point selection in Maxent. Bias files control where background points are selected and the density of background sampling. Proper use of bias files can avoid sampling habitat greatly outside of a species' known occurrence or can account for both collection sampling biases and latitudinal biases associated with coordinate data.

Background points (and similar pseudo-absence points) are meant to be compared with the presence data and help differentiate the environmental conditions under which a species can potentially occur. Typically background points are selected within a large rectilinear area, within this area there often exist habitat that is environmentally suitable, but was never colonized. When background points are selected within these habitats, this increases commission errors (false-positives). As a result, the 'best' performing model tends to be over-fit, because selection criterion favor a model that fail to predict the species in the un-colonized climatically suitable habitat (Anderson & Raza 2010, Barbet-Massin et al. 2012). The likelihood that suitable unoccupied habitats are included in background sampling increases with Euclidian distance from the species' realized range. Thus, a larger study spatial extent can lead to the selection of a higher proportion of less informative background points (Barbet-Massin et al. 2012). Researchers should not avoid studying species with broad distributions or those existing in regions that do not conform well to rectilinear map layouts, rather they simply need to be more selective in the choice of background points in Maxent (and pseudo-absences in other SDM methods)(Barve et al. 2011; Merow et al. 2013).

To circumvent this problem, many researchers have begun using background point and pseudo-absence selection methods that are more regional. SDMtoolbox contains two tools to facilitate more sophisticated background selection for use in Maxent. The *Sample by Distance from Obs. Pts.* tool (see: SDM Tools →2. MaxEnt Tools → Background Selection via Bias Files) uses a common method that samples backgrounds within a maximum radial distance of known occurrences (see Thuiller et al. 2009). The Sample by buffered MCP tool restricts background selection with a buffered minimum-convex polygons based on known occurrences (see following guide).

One limitation of presence-only data SDM methods is the effect of sample selection bias from sampling some areas of the landscape more intensively than others (Phillips et al. 2009). Maxent requires an unbiased sampling of occurrence data and spatial sampling biases can be reduced by using the Gaussian kernel density of sampling localities tool. This method produces a bias grid that up-weights presence-only data points with fewer neighbors in the geographic landscape. To do this the tool creates a Gaussian kernel density of sampling localities (Fig 1n). Output bias values of 1 reflect no sampling bias, whereas higher values represent increased sampling bias. Depending on the study, the input points could be all sampling localities for a larger taxonomic group or simply the input sampling localities of a focal species. For example, if I were studying a single species of frog from Madagascar, I could use either: i)1. only the occurrence points from that species, or ii)2. all sampling points from all amphibians in Madagascar. The former focuses on sampling biases in the focal species, where the latter focuses on

widespread spatial sampling biases and likelihood of detection of your species in all surveys (e.g. sampling only near roads).

Anderson, R. P. & Raza, A. (2010) The effect of the extent of the study region on GIS models of species geographic distributions and estimates of niche evolution: preliminary tests with montane rodents (genus Nephelomys) in Venezuela. *Journal of Biogeography*, 37, 1378-1393.

Barbet-Massin, M., Jiguet, F., Albert, C. H. & Thuiller, W. (2012) Selecting pseudoabsences for species distribution models: how, where and how many? *Methods in Ecology and Evolution,* 3, 327–338.

Phillips SJ, Dudík M, Elith J, Graham CH, Lehmann A, Leathwick J, Ferrier S (2009) Sample selection bias and presence-only distribution models: implications for background and pseudoabsence data. *Ecological Applications*, 19, 181-197.

Thuiller, W., Lafourcade, B., Engler, R. & Araujo, M. B. (2009) BIOMOD – a platform for ensemble forecasting of species distributions. *Ecography*, 32, 369–373.

Background Selection via Bias Files

Tool: Background Selection: Sample by Buffered MCP

ARCGIS STEP-BY-STEP GUIDE:

1. Open a fresh ArcMap document
2. Import 'Uruloke_eowynae.shp' and 'bio_1.asc' (Location: …\example_data\sdm_analyses\ background_selection_via_bias_files)
3. Double-click the 'SDM Tools →2. MaxEnt Tools → Background Selection via Bias Files→ Background Selection: Sample by Buffered MCP' tool
4. Continue to tool interface instructions (below)

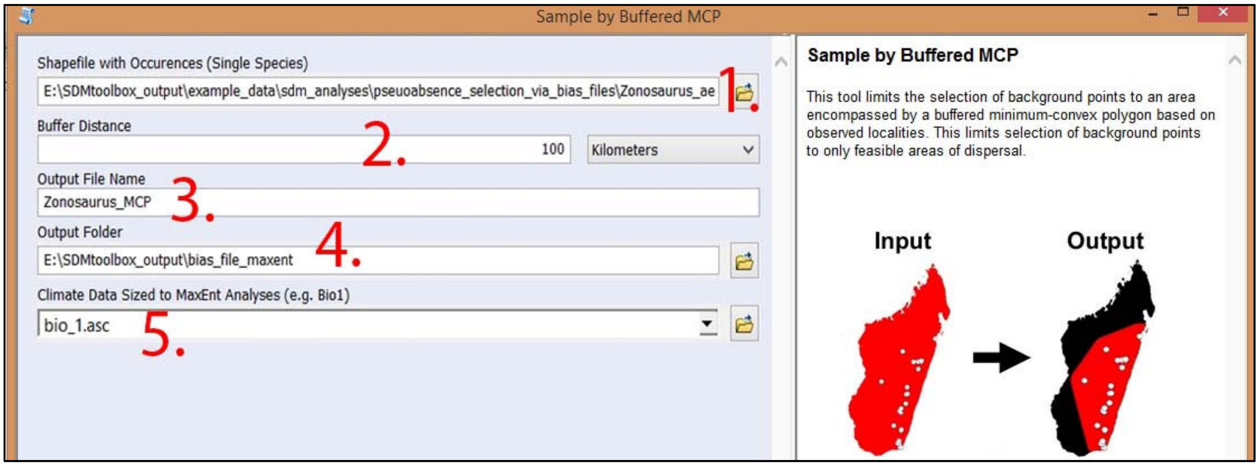

Background Selection: Sample by Buffered MCP **tool interface**

SDMtoolbox Step-by-Step Guide:

1. Shapefile of species occurrence.

> **Tip.** If you need to convert a table to a shapefile for use here. You need two extra columns with latitude and longitude. Then use the tool 'Basic Tools →CSV & Shapefile Tools → 1. CSV to Shapefile'
> -Avoid non-alphanumeric characters in both names (e.g., avoid: * : \ / < > | " ? [ ] ; = + & £ $, etc.).

2. The distance outside of minimum-convex-polygon included in background selection.
3. Output name.
4. Select output folder location. This should be a new empty folder. If not empty this can cause the analysis to fail, particularly if temporary files from a previous analysis were not properly removed (e.g. this can happen if another SDMtoolbox analysis is terminated early).
5. Climate data sized to extent of MaxEnt Modeling. Here use the imported 'Bio_1.asc' layer. Select one of your climate files sized to your modeling extent (e.g. Bio1.asc). This file will be used to match the bias file to proper extent and resolution (no change will be made to this file).

Background Selection via Bias Files

Tool: Background Selection: Sample by Distance from Obs. Pts.

ArcGIS Step-by-Step Guide:

1. Open a fresh ArcMap document
2. Import 'Uruloke_eowynae.shp' and 'bio_1.asc' (Location: …\example_data\sdm_analyses\background_selection_via_bias_files)
3. Double-click the 'SDM Tools →2. MaxEnt Tools→ Background Selection via Bias Files→Sample by Distance from Obs. Pts. '
4. Continue to tool interface instructions (below)

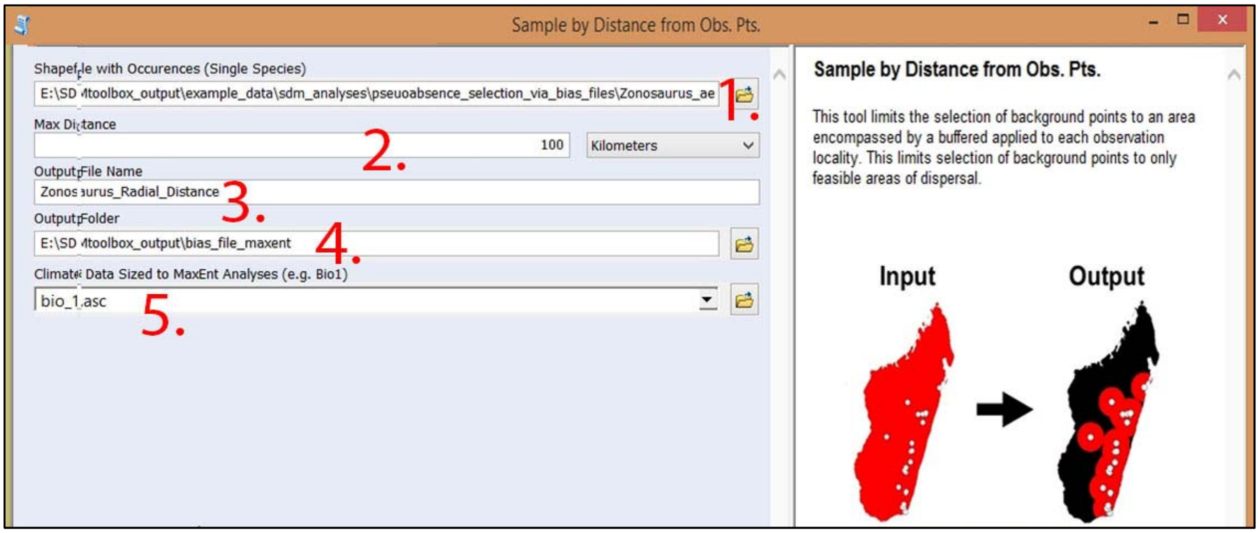

Background Selection: Sample by Distance from Obs. Pts. **tool interface**

SDMtoolbox Step-by-Step Guide:

1. Shapefile of species occurrence.

> **Tip.** If you need to convert a table to a shapefile for use here. You need two extra columns with latitude and longitude. Then use the tool 'Basic Tools →CSV & Shapefile Tools → 1. CSV to Shapefile'
> -Avoid non-alphanumeric characters in both names (e.g., avoid: * : \ / < > | " ? [ ] ; = + & £ $, etc.).

2. The maximum distance away from the observed localities to include in background selection.
3. Output name.
4. Select output folder location. This should be a new empty folder. If not empty this can cause the analysis to fail, particularly if temporary files from a previous analysis were not properly removed (e.g. this can happen if another SDMtoolbox analysis is terminated early).
5. Climate data sized to extent of MaxEnt Modeling. Here use the imported 'Bio_1.asc' layer. Select one of your climate files sized to your modeling extent (e.g. Bio1.asc). This file will be used to match the bias file to proper extent and resolution (no change will be made to this file).

Tool: Background Selection: Sample by Buffered Local Adaptive Convex-Hull

ArcGIS Step-by-Step Guide:

1. Open a fresh ArcMap document
2. Import 'Uruloke_eowynae.shp' and 'bio_1.asc' (Location: …\example_data\sdm_analyses\background_selection_via_bias_files)
3. Double-click the 'SDM Tools →2. MaxEnt Tools → Background Selection via Bias Files→ Background Selection: Sample by Buffered Local Adaptive Convex-Hull' tool
4. Continue to tool interface instructions (following page)

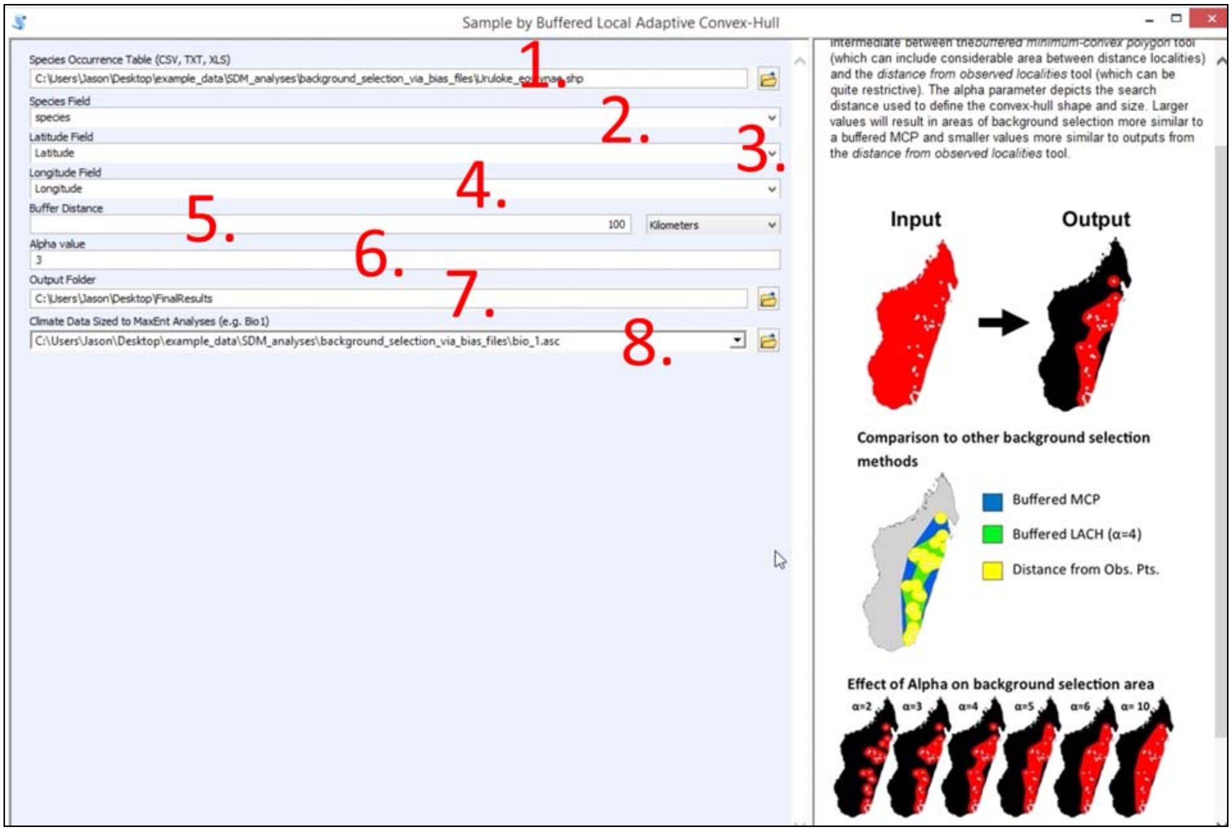

Background Selection: Sample by Buffered Local Adaptive Convex-Hull **tool interface**

SDMtoolbox Step-by-Step Guide:

1. Shapefile of species occurrence.

> **Tip.** If you need to convert a table to a shapefile for use here. You need two extra columns with latitude and longitude. Then use the tool 'Basic Tools →CSV & Shapefile Tools → 1. CSV to Shapefile'
> -Avoid non-alphanumeric characters in both names (e.g., avoid: * : \ / < > | " ? [ ] ; = + & £ $, etc.).

2. Field corresponding to species ID
3. Field corresponding to latitude
4. Field corresponding to longitude
5. The distance outside of polygon(s) included in background selection.
6. The alpha parameter depicts is the search distance used to define the convex-hull shape and size. Larger values will result in areas of background selection more similar to a buffered MCP and smaller values more similar to outputs from the distance from observed localities tool.

   Note. This value is directly linked to the buffer distance (being multiplied by that value). Thus, if you want the same local adaptive convex-hull shape for different buffer distances, you need

adjust the value accordingly. For example, a buffer distance of 50km and α=4 would result in the same adaptive convex-hull shape (prior to buffering) as a buffer distance of 100km and α=2 (50 x 4 = 100 x 2).

7. Select output folder location. This should be a new empty folder. If not empty this can cause the analysis to fail, particularly if temporary files from a previous analysis were not properly removed (e.g. this can happen if another SDMtoolbox analysis is terminated early).
8. Climate data sized to extent of MaxEnt Modeling. Here use the imported 'Bio_1.asc' layer. Select one of your climate files sized to your modeling extent (e.g. Bio1.asc). This file will be used to match the bias file to proper extent and resolution (no change will be made to this file).

UNIVERSAL SDM TOOLS

TOOL OVERVIEW

Create Friction Layer: Invert SDM

The use of least-cost paths and along-path distances often dramatically improve the calculation of geographic distance for testing hypotheses (such as, isolation by distance). However, few studies have access to meaningful friction landscapes. Some researchers (i.e. Broquet et al. 2006) generate friction landscapes from classified satellite images where each major habitat type represents a different value. A primary downfall to using habitat heterogeneity as a friction landscape is the weighing of each habitat class to represent relevant friction values. Doing this properly relies heavily on expert life history knowledge and when done analysis loses some objectivity. For example, Broquet et al. (2006) adjusted the friction values until they satisfied prior expectations. More recently authors used species distribution models (SDMs) as friction landscapes (Wang et al. 2008, Chan et al. 2011). This method is a more objective alternative to expert knowledge and the generation of high quality SDMs can be done with relative ease for many species.

Broquet, T., Ray, N., Petit, E., Fryxell, J.M. & Burel, F. (2006) Genetic isolation by distance and landscape connectivity in the American Marten (*martes americana*). *Landscape Ecology*, **21**, 877-889

Chan LM, Brown JL, Yoder AD (2011). Integrating statistical genetic and geospatial methods brings new power to phylogeography. Mol Phylogenet Evol 59(2):523-37.

Wang, Y.H., Yang, K.C., Bridgman, C.A., Lin, L.K. (2008) Habitat suitability modelling to correlate gene flow with landscape connectivity. *Landscape Ecology*, 23, 989–1000

Create Friction Layer: Invert SDM

Tool: Invert SDM

ARCGIS STEP-BY-STEP GUIDE:

1. Open a fresh ArcMap document
2. Import ‘species_distribution_model.tif’
   (Location: …\example_data\sdm_analyses\create_friction_layer\)
3. Double-click the ‘SDM Tools → 1. Universal Tools → Create Friction Layer → Invert SDM’
4. Continue to tool interface instructions (below)

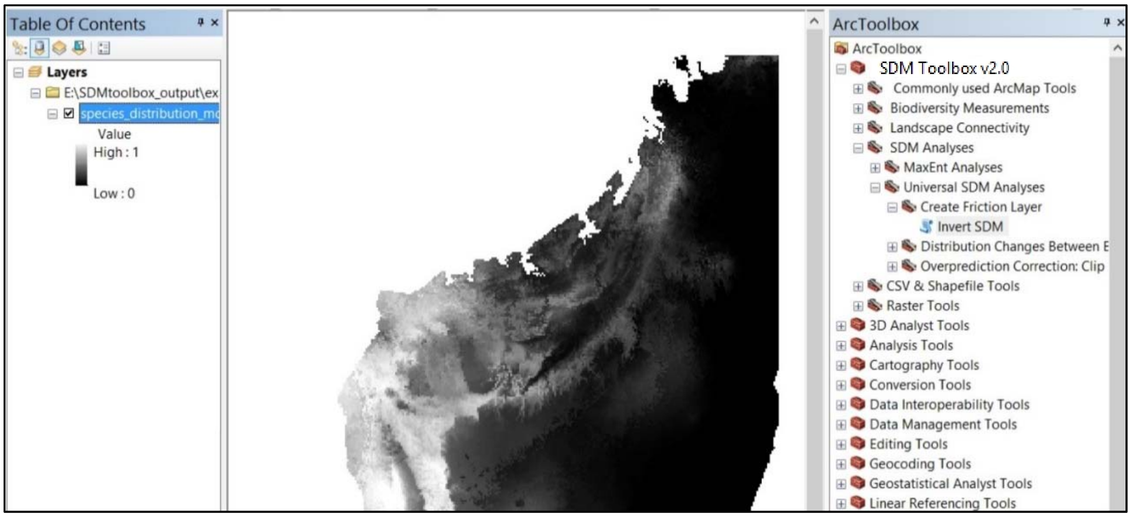

Input data

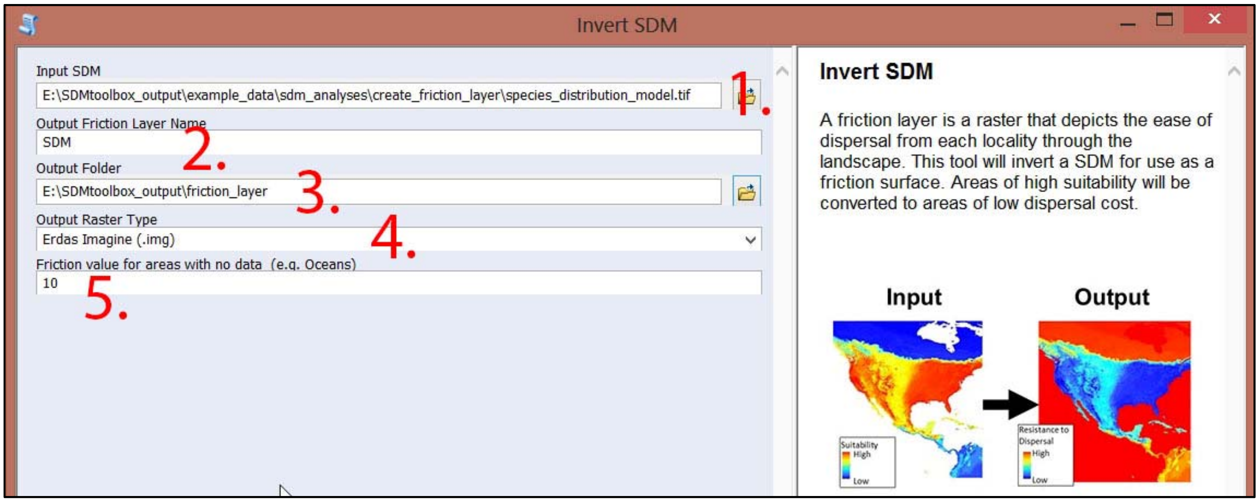

***Invert SDM* tool interface**

SDMtoolbox Step-by-Step Guide:

1. Input species distribution model (SDM)
2. Name of output file. Note: '_FrictionLayer' will be appended to the end of output name.
3. Select output folder location. This should be a new empty folder. If not empty this can cause the analysis to fail, particularly if temporary files from a previous analysis were not properly removed (e.g. this can happen if another SDMtoolbox analysis is terminated early).
4. Output raster format. Here chose 'ERDAS Imagine (.img)'
5. Areas of 'NoData' (cells that lack SDM values, i.e. oceans) can be converted to friction values. This is particularly important if using frictions layers to connect populations isolated on islands and mainland populations.

Results

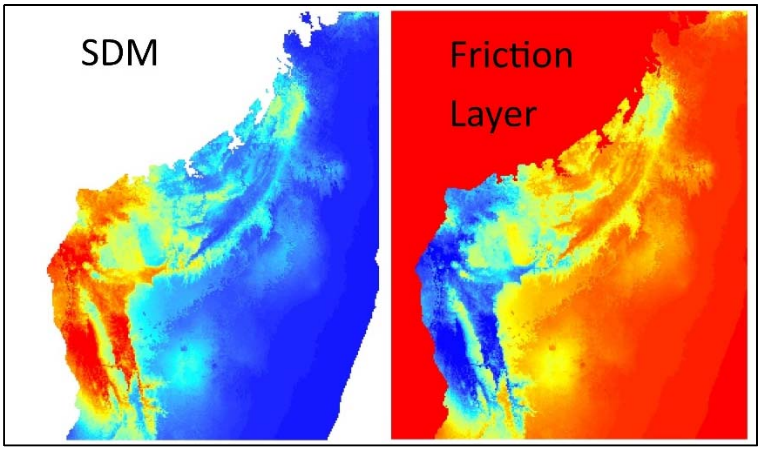

***Invert SDM* tool.** Right: Input SDM. Left: Output friction layer

UNIVERSAL SDM TOOLS

TOOL OVERVIEW

Spatially Rarefy Occurrence Data

Most SDM methods require input occurrence data to be spatially independent to perform well. However, it is common for researchers to introduce environmental biases into their SDMs from spatially autocorrelated occurrence points. The elimination of spatial clusters of localities is important for model calibrating and evaluation. When spatial clusters of localities exist, often models are over-fit towards environmental biases (reducing the model's ability to predict spatially independent data) and model performance values are inflated (Veloz 2009; Hijimans *et al.* 2012; Boria *et al.* 2014). The *spatially rarefy occurrence data* tool addresses this issue by spatially filtering locality data by a user input distance, reducing occurrence localities to a single point within the specified Euclidian distance. This tool also allows users to spatially rarefy their data at several distances according to habitat, topographic or climate heterogeneity (Table 1d). For example, occurrence localities could be spatially filtered at 5 km^2, 10 km^2 and 30 km^2 in areas of high, medium and low environmental heterogeneity, respectively. This graduated filtering method is particular useful for studies with limited occurrence points and can maximize the number of spatially independent localities.

Veloz, S. D. (2009) Spatially autocorrelated sampling falsely inflates measures of accuracy for presence-only niche models. *Journal of Biogeography,* 36, 2290–2299.
Hijmans, R.J. (2012) Cross-validation of species distribution models: removing spatial sorting bias and calibration with a null model. *Ecology,* 93, 679–688.
Boria R. A., Olson L.E., Goodman S.M. & Anderson R.A. (2014) Spatial filtering to reduce sampling bias can improve the performance of ecological niche models. *Ecological Modeling*, 275, 73-77.

Spatially Rarefy Occurrence Data

Tool: Spatially Rarefy Occurrence Data

ARCGIS STEP-BY-STEP GUIDE:

1. Open a fresh ArcMap document
2. Import 'occurrences.shp' and 'climate_hetero.tif'
   (Location: …\example_data\sdm_analyses\rarefy\)
3. Double-click the 'SDM Tools → 1. Universal Tools → Spatially Rarefy Occurrence Data for SDMs (reduce spatial autocorrelation)' tool
4. Continue to tool interface instructions (below)

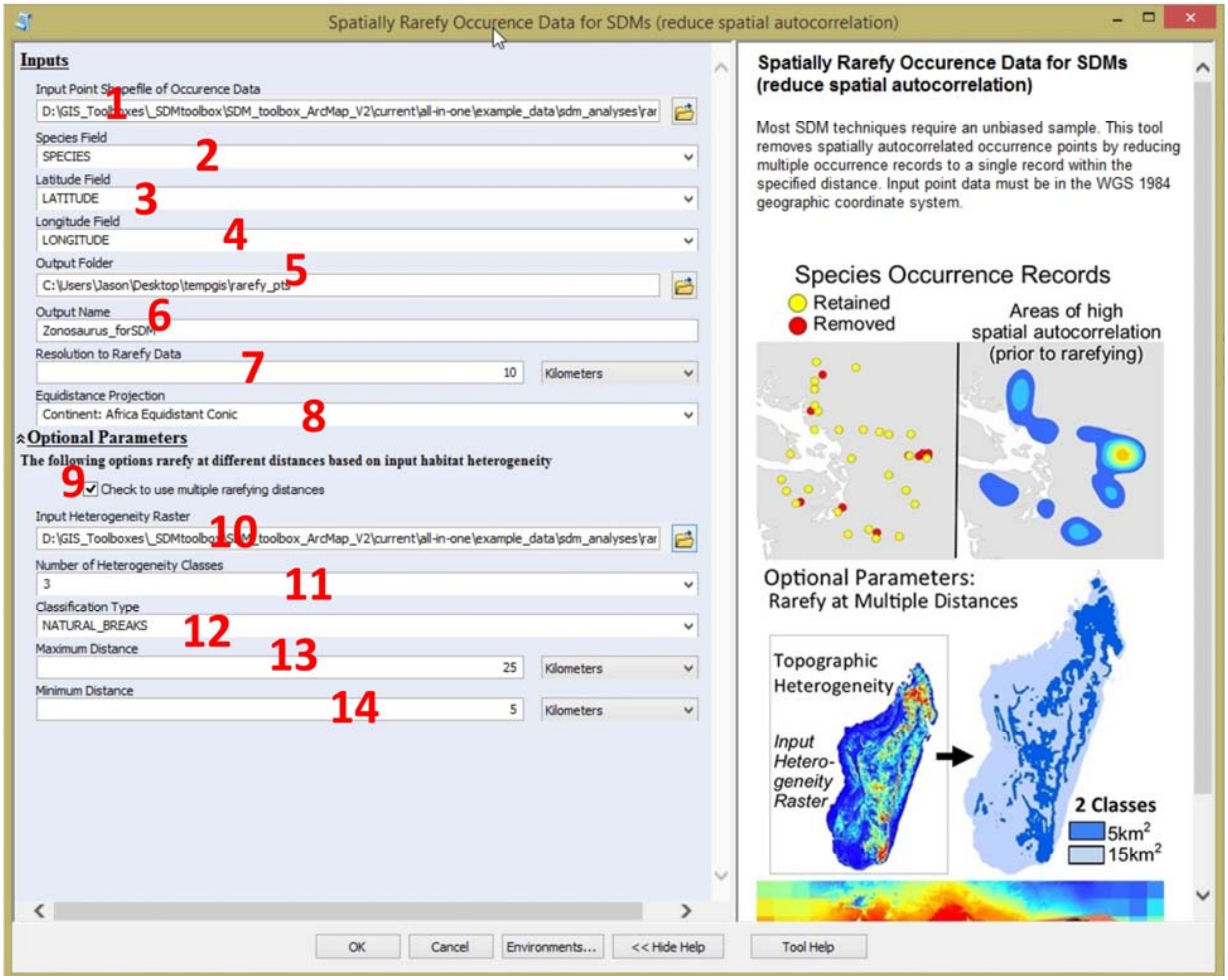

Spatially Rarefy Occurrence Data **tool interface**

SDMtoolbox Step-by-Step Guide:

1. Input shapefile of species occurrence data, here select ‘occurences.shp’
2. Shapefile field corresponding to species identity, here select ‘SPECIES’
3. Shapefile field corresponding to latitude, here select ‘LATITUDE’
4. Shapefile field corresponding to longitude, here select ‘LONGITUDE’
5. Select output folder location. This should be a new empty folder. If not empty this can cause the analysis to fail, particularly if temporary files from a previous analysis were not properly removed (e.g. this can happen if another SDMtoolbox analysis is terminated early).
6. Name of output file. Note that "_rarefied_points" will be appended to file name.
7. The spatial resolution to rarefy the data. Here use the default settings. Note that this value will NOT actually be used here because you will be executing the multi-distance occurrence data rarefying.
8. Please select ‘Continent: Africa Equidistant Conic’
9. Placing a check mark in the box to execute the multi-distance data rarefying

10. Input heterogeneity raster, here the “climate_hetero.tif’ This is require if using the multi-distance data rarefying
11. The number of classes, here 5
12. Classification type, here ‘NATURAL_BREAKS’
13. The maximum distance, here 25 Kilometers
14. The minimum distance, here 2 Kilometers

UNIVERSAL SDM TOOLS

TOOL OVERVIEW

Split binary SDM by input clade relationship

This script will split a binary SDM by user input clade relationships. The current script only supports clade numbers of 2 to 10 groups. If only a single clade is identified, there will be no change splitting of the binary SDM. If more than 10 clade IDs exist for a species this will be skipped.

Split binary SDM by input clade relationship

ARCGIS STEP-BY-STEP GUIDE:

1. Open a fresh ArcMap document
2. Import ‘occurence_points.csv’
   (Location: …\example_data\sdm_analyses\split_by_clade\)
3. Double-click the ‘SDM Tools → 1. Universal Tools → Split binary SDM by input clade relationship’
4. Continue to tool interface instructions (below)

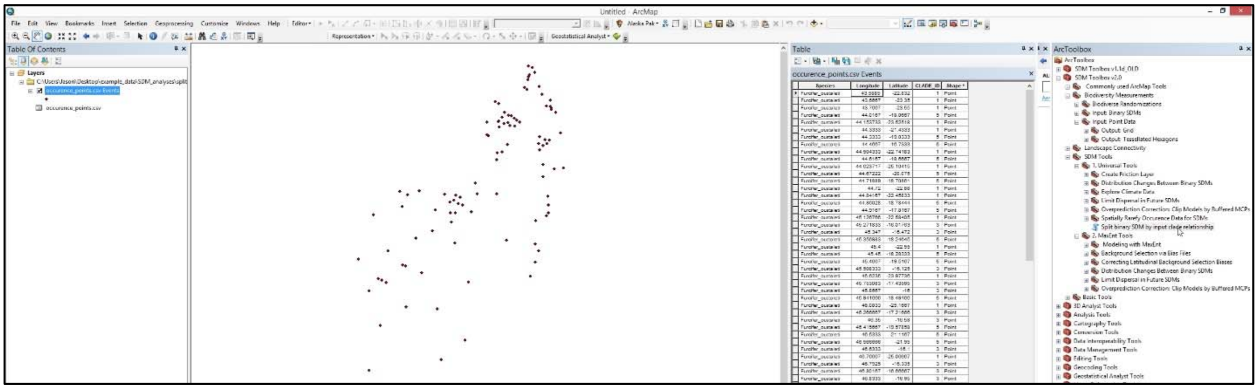

	Species	Longitude	Latitude	CLADE_ID	Shape *
▶	Furcifer_oustaleti	43.5885	-22.832	1	Point
	Furcifer_oustaleti	43.6667	-23.35	1	Point
	Furcifer_oustaleti	43.7667	-23.55	1	Point
	Furcifer_oustaleti	44.0167	-18.0667	5	Point
	Furcifer_oustaleti	44.153733	-23.52518	1	Point
	Furcifer_oustaleti	44.3333	-21.4333	1	Point
	Furcifer_oustaleti	44.3333	-19.0333	5	Point
	Furcifer_oustaleti	44.4667	-16.7333	5	Point
	Furcifer_oustaleti	44.504333	-22.74183	1	Point
	Furcifer_oustaleti	44.6167	-18.6667	5	Point
	Furcifer_oustaleti	44.623717	-25.10415	1	Point
	Furcifer_oustaleti	44.67222	-20.075	5	Point
	Furcifer_oustaleti	44.71889	-18.70861	5	Point
	Furcifer_oustaleti	44.72	-22.88	1	Point
	Furcifer_oustaleti	44.84167	-22.45833	1	Point
	Furcifer_oustaleti	44.86028	-18.78444	5	Point
	Furcifer_oustaleti	44.9167	-17.8167	5	Point

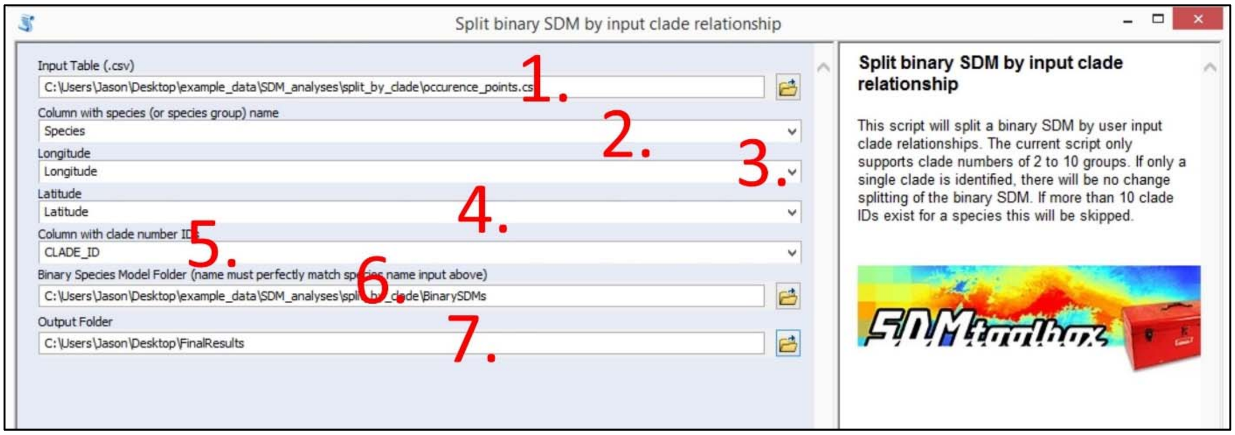

Split binary SDM by input clade relationship **tool interface**

SDMtoolbox Step-by-Step Guide:

1. Input Table with occurrence data, species ID, and clade ID (as number: 2-10). The current script only supports clade numbers of 2 to 10 groups. If only a single clade is identified, there will be no change splitting of the binary SDM. If more than 10 clade IDs exist for a species this will be skipped.
2. Column with species (or species group) name
3. Column with longitude values
4. Column with latitude values
5. Column with clade ID
6. Select folder with binary species model (name must perfectly match species name input in table)
7. Output folder location. This should be a new empty folder. If not empty this can cause the analysis to fail, particularly if temporary files from a previous analysis were not properly removed (e.g. this can happen if another SDMtoolbox analysis is terminated early).

Results

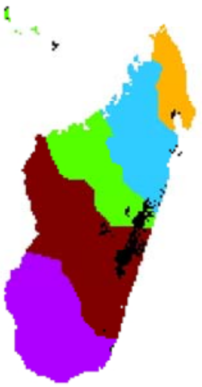

Split binary SDM by input clade relationship **results**

Left: Final distribution split by clade relationship. **Below:** Over view of method, which split the landscape by thiessen for each locality. Then associated with clade membership is applied to groups which are use to divide input binary SDM into associated clade groups. Output are individual rasters for each clade and a single file with all clades in one raster.

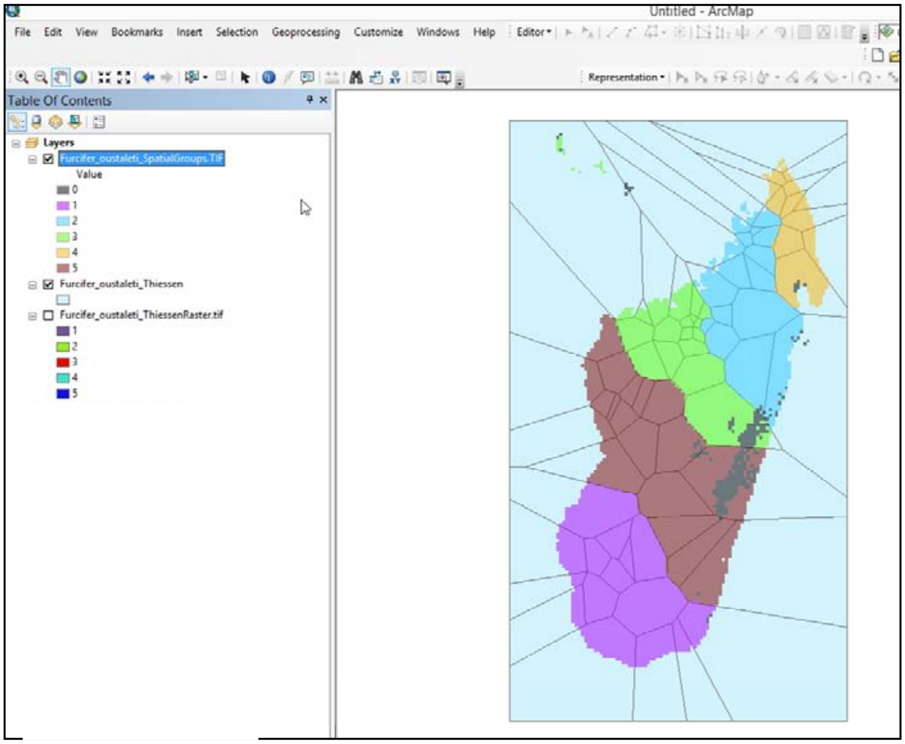

Chapter 4. Raster, CSV & Shapefile tools

Tool: *CSV, TXT, XLS to shapefile*

ArcGIS Step-by-Step Guide:

1. Open a fresh ArcMap document
2. Import 'Uruloke_spp.csv'
   (Location: …\example_data\SDM_analyses\correcting_lat_BPs\soultion2\)
3. Double-click the 'Basic Tools→ CSV & Shapefile Tools → 1. CSV to Shapefile'
4. Continue to tool interface instructions (below)

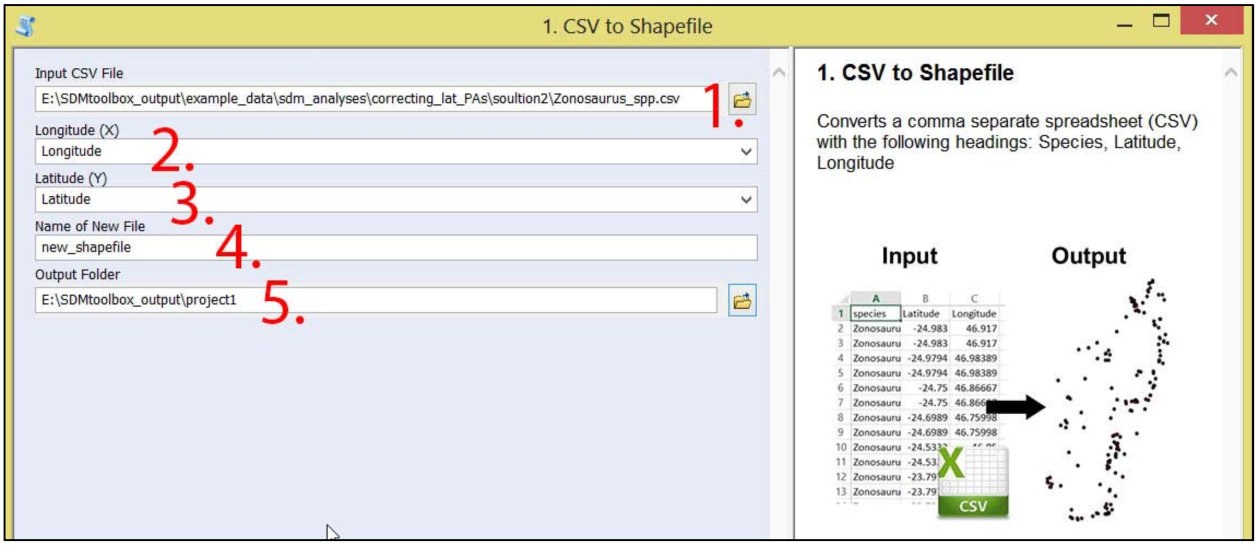

CSV to Shapefile tool interface

SDMtoolbox Step-by-Step Guide:

1. Input CSV file with columns with latitude and longitude
2. Field with longitude
3. Field with latitude
4. Name of new shapefile
5. Select output folder location

Chapter 5. Running a SDM in MaxEnt: from Start to Finish

Below is a brief overview of *my view* of the **best practices** of correlative species distribution modeling and how SDMtoolbox will facilitate achieving them. This overview focuses on modeling in MaxEnt, but many steps are applicable to all types of distribution modeling. For overview of major assumptions and other considerations, see table at the end of this document.

Species distribution modelling (SDM) occurs in two phases: 1) **Data compilation** and 2) **Model creation, calibration, and validation**

Data compilation

This step includes collecting occurrence records of the focal species and environment data for its habitats.

Occurrence Data

The single most important component of any SDM is the input occurrence records. Extra care should go into selecting, and then processing, these points. The quality, distribution and number of points are directly related to the accuracy of the model. Use as many high-quality locality points as possible (e.g. GPS data collected with confident taxonomic identification) and try to collect occurrence records that are evenly sampled throughout the species' range and avoid biases in the sampling method (e.g., sampling only from road transects). It is better to have only a limited number of points that satisfy the above conditions than many points of vague credence (e.g. be skeptical of points downloaded from internet databases, particularly those that are georeferenced from locality info) (Chan et al. 2011).

Environment Data

The environment data provide the landscape-level data to quantify the focal species' ecological tolerances. Include variables that are likely to be directly relevant to the species being modeled. However do not add all available climate data without regard to the redundancy of the data. Many environmental variables are tightly correlated making some redundant, this makes interpreting the influences of each variable in the model difficult. If not included in your model, consider the effects of the following items on the present distribution of your species: fire history, glaciations, contagious diseases, anthropogenic factors, and recent geological changes, the species' movement potential through the landscape or biotic interactions.

1. Preparing Worldclim Climate Data for use in MaxEnt Analyses

1A. Preparing Worldclim Climate Data: Clip the raster to area of species' extent

Tools: Extract by Mask (Folder) and Raster to ASCII

ARCGIS STEP-BY-STEP GUIDE:

1. Open a fresh ArcMap document
2. Download ESRI grid climate data (e.g. the 30 arc-second bioclim) from worldclim.org
3. Open one of the newly downloaded layers in ArcMap

4. There are several ways to define the area to clip the climate data into. One of the easiest ways is to simply zoom the display window the desired extent (Image below, where I wanted to reduce climate data to Madagascar). Other ways include defining the max-min XY coordinates (a bounding box) or using another GIS layer as a templates (such as, a country's boundary). Select one of these methods and continue. Note the area should encompass an area about 50-100 km (ca. 0.5-1 degree) greater than total distribution of **all** your focal species. We will then use bias files to limit background selection of each species to meaningful areas within this area.

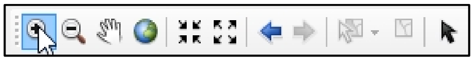

Zoom tool

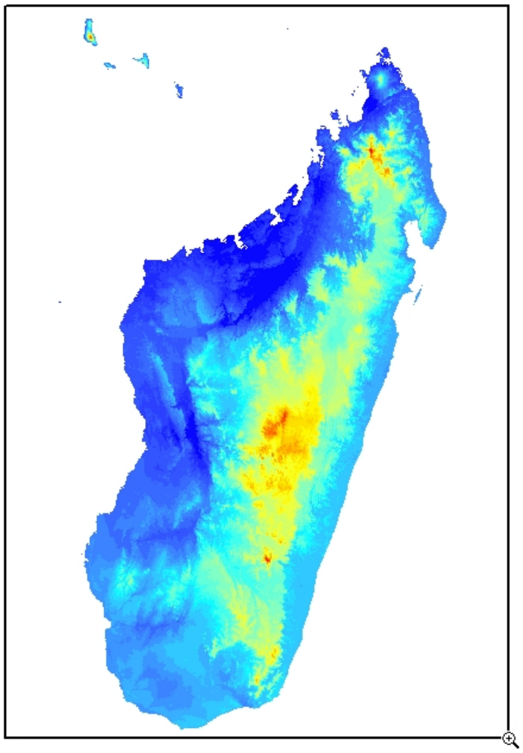

Zoom to desired area

5. Double-click the 'Basic Tools →Raster Tools → 1. Extract by Mask (Folder)
6. Continue to tool interface instructions (below)

SDMTOOLBOX STEP-BY-STEP GUIDE:

1. Input folder containing the full extent Worldclim data, input raster should be ESRI grid format
2. Check the box depicting that the rasters are ESRI grid format.
3. Select output folder location
4. Select either a 'mask' or select 'extent' and choose the appropriate method for defining the extent (here I used 'Same as Display').
5. Execute tool

1B. Preparing Worldclim Climate Data: Convert rasters to ASCII

Tool: Raster to ASCII

ARCGIS STEP-BY-STEP GUIDE:

1. Double-click the 'Basic Tools →Raster Tools → 2a. Raster to ASCII (Folder)
2. Continue to tool interface instructions (below)

SDMTOOLBOX STEP-BY-STEP GUIDE:

1. Input folder containing the clipped Worldclim data output from previous tool
2. Select raster type "Tiff(.tif)"
3. Select output folder location
4. The climate data are ready for use in MaxEnt

1C. Preparing Worldclim Climate Data: Define projection of ASCII climate data

Tool: 3d. Define Projection as WGS84 or ArcMaps's Define Projection

***IF* input data are coordinates and WGS84 then:**

ARCGIS STEP-BY-STEP GUIDE:

1. Double-click the 'Raster Tools → 3d. Define Projection as WGS84 (folder)'
2. Continue to tool interface instructions (below)

SDMTOOLBOX STEP-BY-STEP GUIDE:

1. Input folder with clipped ASCII worldclim data.
2. Execute tool

***IF* input data are another projection, use ArcMap's tool: "Define Projection".**

2. Optional Step. Which variables should I use? Testing Autocorrelations of Environmental Data

If you are interested interpreting how each input environmental variable contributes to your species distribution model, then you need to reduce autocorrelation of your input environmental data by removing highly correlated variables. It is widely known that many climate variables are highly correlated with each other. While including all these will not affect the predictive quality of your MaxEnt model, it does seriously limit any inference of the contribution of any correlated variables (*i.e.* the MaxEnt outputs from 'Analysis of variable contributions' and to some degree 'Jackknifing environmental variables'). This is mainly because when a model is built in MaxEnt, if a highly correlated variable is included in the model, this often excludes all other highly correlated variables from being incorporated. This is because these variables likely would contribute similarly to the models. Since they are not included, they will not be properly represented in the output 'Analysis of variable contributions'.

Tool: Explore Climate Data: Remove Highly Correlated Variables

ArcGIS Step-by-Step Guide:

1. Double-click the 'SDM Tools → Universal SDM Tools → Explore Climate Data → Remove Highly Correlated Variables '
2. Continue to tool interface instructions (below)

NOTE: the tool below will fail if raster files are too large. This bug is associated with SciPy and is nothing I can fix. If you are really interested in performing this analysis- I suggest you reduce spatial scale of environmental rasters to 10-20km^2 and run this tool. Given very high spatial-autocorrelation in these layers, values below this typically wont greatly affect the correlations associated with this analysis

SDMtoolbox Step-by-Step Guide:

1. Select all the clipped Worldclim data ('control+shift' will allow you to select all items in a folder). Layers that you wish to retain (vs. the other correlated layers) should be first in the list. All correlated layers that occur after will be excluded. For interpreting influence of environmental layers in the SDM, I prefer to place layers that depict metrics frequently used in non-SDM ecology and evolution studies [such as: BIO1 = Annual Mean Temperature, BIO2 = Mean Diurnal Range (Mean of monthly (max temp - min temp)), BIO12 = Annual Precipitation]. Further for simplicity, these layers often best represent the original input climate data (as they directly reflect the actual measurements) and are not derived from several layers or a subset of the data.
2. Maximum correlation allowed. Multiple values can be input separated by semicolon (';'). Input a value between 0-1. The absolute value of the correlation coefficients range from 0 to 1. A value of 1 implies that a linear equation describes the relationship between X and Y perfectly. A value of 0 implies that there is no linear correlation between the variables.
3. Input NoData Value. Note this must be the same for all values or else correlations will not be accurate. Since we used only Worldclim here, this should not be an issues (as all values are the

same). To check NoData values, import layers into ArcGIS and right click the layer and select 'Properties' and then go to the 'Source' tab. Alternatively, you can simply open your ".asc" files in a text editor and at the top of header will be the NoData value.

4. Select output folder location. Output will be two tables with the correlation coefficients among all comparisons and a table with the final list of rasters to include in your model.

3. Preparing Occurrence Data for use in MaxEnt Analyses

3A. Preparing Occurrence Data: Import Species Occurrence Records

Tool: *CSV, TXT, XLS to shapefile*

ARCGIS STEP-BY-STEP GUIDE:

1. Open a fresh ArcMap document
2. Import CSV, TXT or XLS file with occurrence records. Table of species occurrences containing ONLY: Species ID, Longitude and Latitude. The fields must be in that order. For species ID, do not use non-alphanumeric characters in names (e.g., * : \ / < > | " ? [ ] ; = + & £ $), replace spaces with "_", and remove periods.
3. Double-click the 'CSV & Shapefile Tools → 1. CSV to Shapefile'
4. Continue to tool interface instructions (below)

species	Longitude	Latitude
Zonosaurus_aeneus	46.917	-24.983
Zonosaurus_aeneus	46.917	-24.983
Zonosaurus_aeneus	46.98389	-24.97944
Zonosaurus_aeneus	46.98389	-24.97944
Zonosaurus_aeneus	46.86667	-24.75

MaxEnt Input Species Data Format: three columns with species, longitude and latitude--- as ordered here.

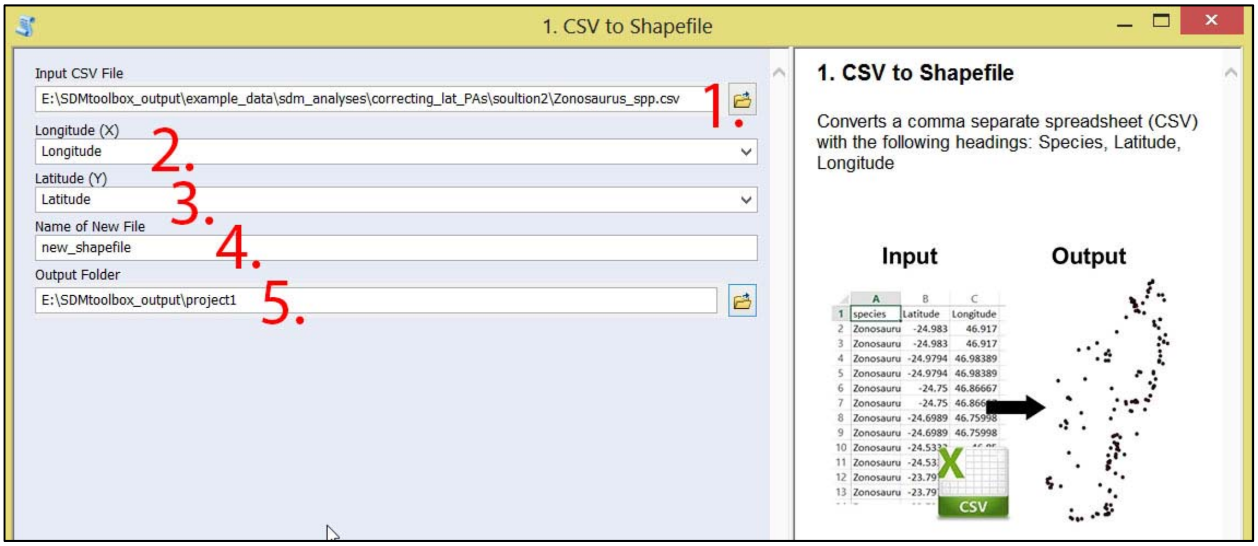

CSV to Shapefile tool interface

SDMTOOLBOX STEP-BY-STEP GUIDE:

1. Input CSV file with columns with latitude and longitude
2. Field with longitude
3. Field with latitude
4. Name of new shapefile
5. Select output folder location

3B. Preparing Occurrence Data: Define projection of occurrence points shapefile

Tool: 6b. Define Projection as WGS84 or ArcMaps's Define Projection

ARCGIS STEP-BY-STEP GUIDE:

IF input data are coordinates and WGS84 (if not see below):

1. Double-click the 'CSV & Shapefile Tools → 6b. Define Projection as WGS84'
2. Continue to tool interface instructions (below)

SDMTOOLBOX STEP-BY-STEP GUIDE:

1. Input newly imported shapefile points
2. Execute tool

IF input data are another projection, use ArcMap's tool: "Define Projection".

3C. Preparing Occurrence Data: PCA of Climate Variables to Estimate Heterogeneity

Tool: 2a. Calculate Climate Heterogeneity: Step 1- Principal Component Analysis

ARCGIS STEP-BY-STEP GUIDE:

1. Double-click the 'SDM Tools → 1. Universal Tools → Spatially rarify occurrence data for SDMs → 2a. Calculate Climate Heterogeneity: Step 1- Principal Component Analysis' tool
2. Continue to tool interface instructions (below)

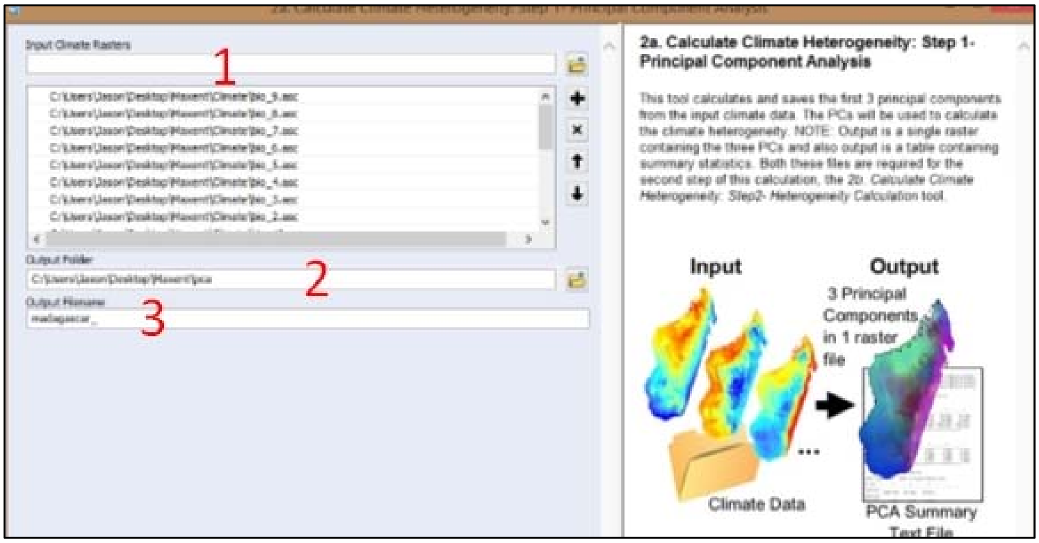

2a. Calculate Climate Heterogeneity: Step 1- Principal Component Analysis tool interface

SDMtoolbox Step-by-Step Guide:

1. Input all your climate rasters that depict continuous data. Note: hold 'shift' to select many rasters at once.
2. Select output folder location. This should be a new empty folder. If not empty this can cause the analysis to fail, particularly if temporary files from a previous analysis were not properly removed (e.g. this can happen if another SDMtoolbox analysis is terminated early).
3. Name of output file. Note that "_Climate_PCs" will be appended to file name.

To visualize all three bands output from the PCA tool, right click layer and select ‘Properties’. Then select the ‘Symbology’ tab and select ‘RGB Composite’. The output raster depicts climate space: the more similar the colors the more similar values.

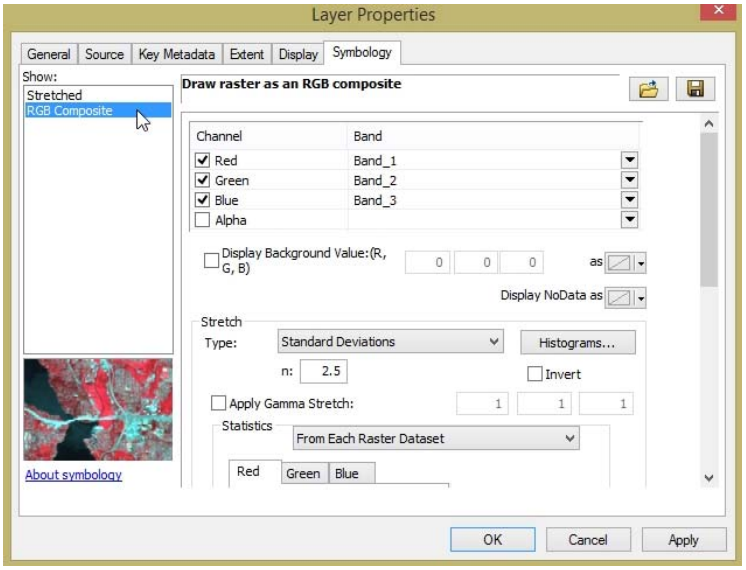

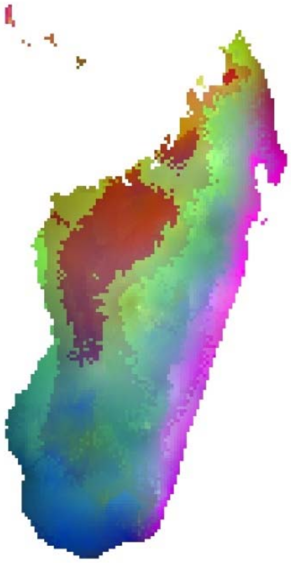

3D. Preparing Occurrence Data: Measure Spatial Heterogeneity of Climate PCs

Tool: 2b. Calculate Climate Heterogeneity: Step 2- Heterogeneity Calculation

ARCGIS STEP-BY-STEP GUIDE:

1. Double-click the ‘SDM Tools → 1. Universal Tools →Spatially rarify occurrence data for SDMs → 2b. Calculate Climate Heterogeneity: Step 2- Heterogeneity Calculation’ tool
2. Continue to tool interface instructions (below)

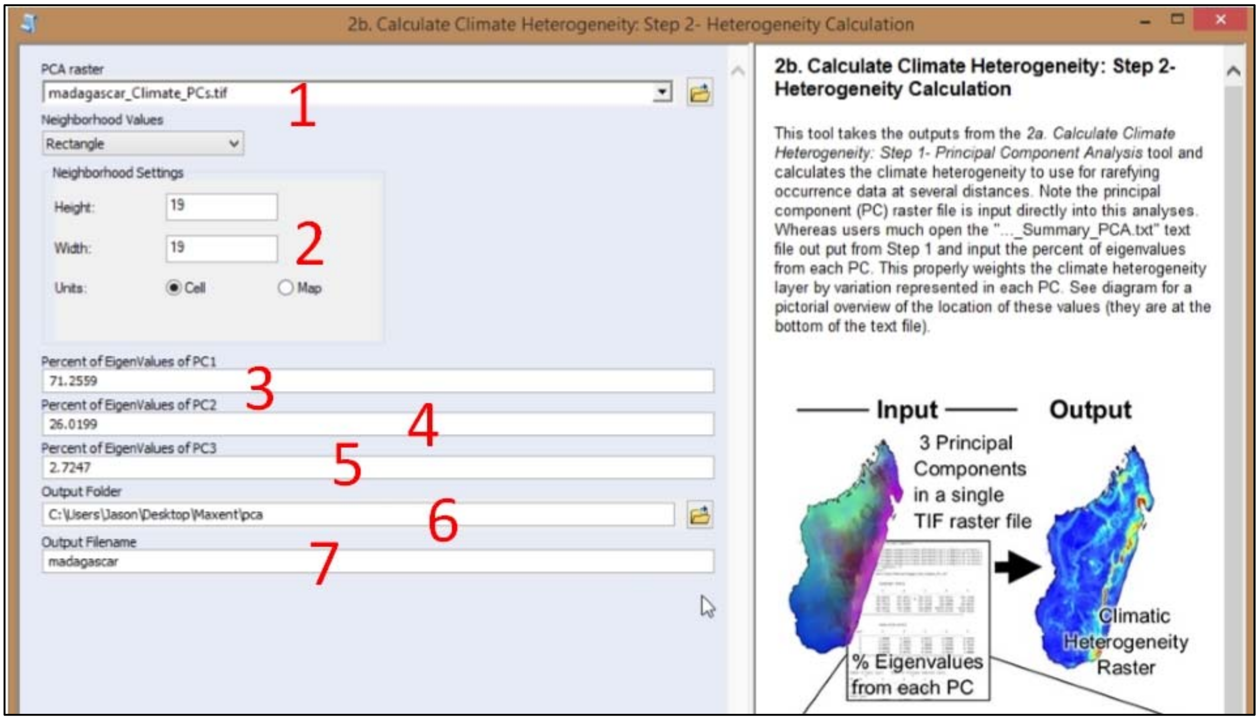

2b. Calculate Climate Heterogeneity: Step 2- Heterogeneity Calculation tool interface

SDMTOOLBOX STEP-BY-STEP GUIDE:

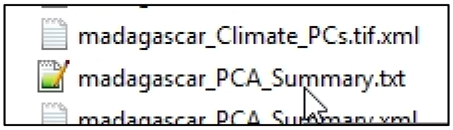

Output PCA Summary table

1. Input climates PCA raster output from previous step
2. This is the spatial scale used to calculate the heterogeneity of the landscape. E.g., if 3 x 3 rectangle and cell units are selected: heterogeneity values will be calculated from each raster pixel and the 8 cells neighboring the focal cell.
3. Percent of EigenValues of PC1. This value is input from the "..._PCA_summary.txt" file. Go to the bottom of the text file to the heading 'PRECENT AND ACCUMULATIVE

PERCENT AND ACCUMULATIVE EIGENVALUES

PC Layer	EigenValue	Percent of EigenValues	Accumulative of EigenValues
1	167483.51078	71.2559	71.2559
2	61158.52418	26.0199	97.2758
3	6403.13672	2.7242	100.0000

Section of the table that is necessary for input into this tool

EIGENVALUES' and go to the row corresponding to PC1 and input the value from the 'Percent of EigenValues'.

4. Percent of EigenValues of PC2.
5. Percent of EigenValues of PC3.
6. Output Folder
7. Output filename. Note: "_clim_hetero.tif" will automatically be appended.

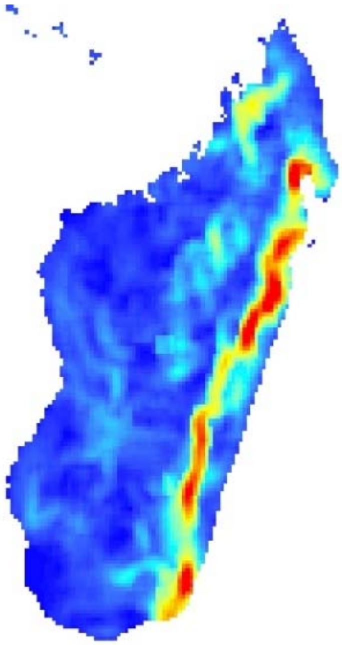

Above. **Output climate heterogeneity raster. Here warm colors depict high areas of climatic heterogeneity.**

3E. Preparing Occurrence Data: Graduated Spatial Rarefying

An Unbiased Sample: a Need for Spatial Rarefying

Most SDM methods require input occurrence data to be spatially independent to perform well. However, it is common for researchers to introduce environmental biases into their SDMs from spatially auto-correlated occurrence points. The elimination of spatial clusters of localities is important for model calibrating and evaluation. When spatial clusters of localities exist, often models are over-fit towards environmental biases (reducing the model's ability to predict spatially independent data) and model performance values are inflated (Veloz 2009; Hijimans *et al.* 2012; Boria *et al.* 2014). The *spatially rarefy occurrence data* tool addresses this issue by spatially filtering locality data by a user input distance, reducing occurrence localities to a single point within the specified Euclidian distance. This tool also allows users to spatially rarefy their data at several distances according to habitat, topographic or climate heterogeneity (Table 1d). For example, occurrence localities could be spatially filtered at 5 km^2, 10 km^2 and 30 km^2 in areas of high, medium and low environmental heterogeneity, respectively. This graduated filtering method is particular useful for studies with limited occurrence points and can maximize the number of spatially independent localities.

-Veloz, S. D. (2009) Spatially autocorrelated sampling falsely inflates measures of accuracy for presence-only niche models. *Journal of Biogeography,* 36, 2290–2299.
-Hijmans, R.J. (2012) Cross-validation of species distribution models: removing spatial sorting bias and calibration with a null model. *Ecology,* 93, 679–688.
-Boria R. A., Olson L.E., Goodman S.M. & Anderson R.A. (2014) Spatial filtering to reduce sampling bias can improve the performance of ecological niche models. *Ecological Modeling,* 275, 73-77.

Tool: Spatially Rarefy Occurrence Data

ARCGIS STEP-BY-STEP GUIDE:

1. Open a fresh ArcMap document
2. Import 'occurrences.shp'
   (Location: …\example_data\SDM_analyses\rarefy\)
3. Double-click the 'SDM Tools → 1. Universal Tools → Spatially Rarefy Occurrence Data for SDMs (reduce spatial autocorrelation)' tool
4. Continue to tool interface instructions (below)

SDMTOOLBOX STEP-BY-STEP GUIDE:

1. Input shapefile of species occurrence data, here select 'occurences.shp'
2. Shapefile field corresponding to species identity, here select 'SPECIES'
3. Shapefile field corresponding to latitude, here select 'LATITUDE'
4. Shapefile field corresponding to longitude, here select 'LONGITUDE'
5. Select output folder location. This should be a new empty folder. If not empty this can cause the analysis to fail, particularly if temporary files from a previous analysis were not properly removed (e.g. this can happen if another SDMtoolbox analysis is terminated early).
6. Name of output file. Note that "_rarefied_points" will be appended to file name.
7. The spatial resolution to rarefy the data. Here use the default settings. Note that this value will NOT actually be used here because you will be executing the multi-distance occurrence data rarefying.
8. Please select proper equidistance projection.
9. Placing a check mark in the box to execute the multi-distance data rarefying

10. Input heterogeneity raster.
11. The number of classes, here 5

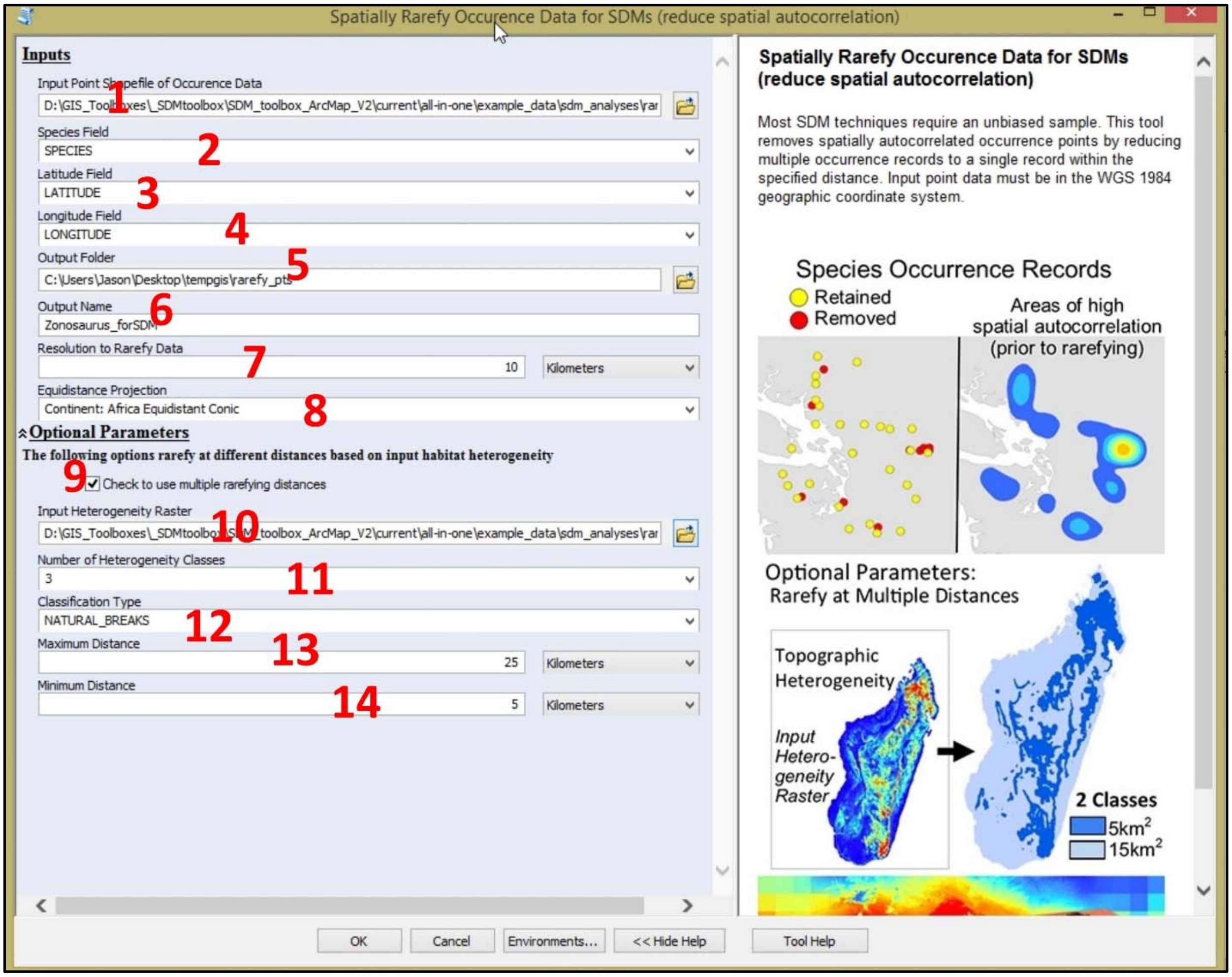

12. Classification type, here 'NATURAL_BREAKS'
13. The maximum distance, here 25 Kilometers
14. The minimum distance, here 2 Kilometers

Spatially Rarefy Occurrence Data **tool interface**

4. Creation of Bias Files

Background Selection via Bias Files

A subset of python scripts create bias files used to fine-tune background and occurrence point selection in Maxent. Bias files control where background points are selected and the density of background sampling. Proper use of bias files can avoid sampling habitat greatly outside of a species' known occurrence or can account for collection sampling biases with coordinate data.

Background points (and similar pseudo-absence points) are meant to be compared with the presence data and help differentiate the environmental conditions under which a species can potentially occur. Typically background points are selected within a large rectilinear area, within this area there often exist habitat that is environmentally suitable, but was never colonized. When background points are selected within these habitats, this increases commission errors (false-positives). As a result, the 'best' performing model tends to be over-fit because selection criterion favor a model that fail to predict the species in the un-colonized climatically suitable habitat (Anderson & Raza 2010, Barbet-Massin et al. 2012). The likelihood that suitable unoccupied habitats are included in background sampling increases with Euclidian distance from the species' realized range. Thus, a larger study spatial extent can lead to the selection of a higher proportion of less informative background points (Barbet-Massin et al. 2012). Researchers should *not* avoid studying species with broad distributions or those existing in regions that do not conform well to rectilinear map layouts, rather they simply need to be more selective in the choice of background points in Maxent (and pseudo-absences in other SDM methods)(Barve et al. 2011; Merow et al. 2013).

To circumvent this problem, many researchers have begun using background point and pseudo-absence selection methods that are more regional. SDMtoolbox contains three tools to facilitate more sophisticated background selection for use in Maxent. The *Sample by Distance from Obs. Pts.* tool (see: SDM Tools →2. MaxEnt Tools → Background Selection via Bias Files) uses a common method that samples backgrounds within a maximum radial distance of known occurrences (see Thuiller et al. 2009). The Sample by buffered MCP tool restricts background selection with a buffered minimum-convex polygons based on known occurrences (see following guide).

-Anderson, R. P. & Raza, A. (2010) The effect of the extent of the study region on GIS models of species geographic distributions and estimates of niche evolution: preliminary tests with montane rodents (genus Nephelomys) in Venezuela. *Journal of Biogeography*, 37, 1378-1393.
-Barbet-Massin, M., Jiguet, F., Albert, C. H. & Thuiller, W. (2012) Selecting pseudoabsences for species distribution models: how, where and how many? *Methods in Ecology and Evolution,* 3, 327–338.
-Phillips SJ, Dudík M, Elith J, Graham CH, Lehmann A, Leathwick J, Ferrier S (2009) Sample selection bias and presence-only distribution models: implications for background and pseudoabsence data. *Ecological Applications*, 19, 181-197.
-Thuiller, W., Lafourcade, B., Engler, R. & Araujo, M. B. (2009) BIOMOD – a platform for ensemble forecasting of species distributions. *Ecography*, 32, 369–373.

Tool: Background Selection: Sample by Buffered MCP, Sample by Distance from Obs. Pts. or Sample by Buffered Local Adaptive Convex-Hull

ARCGIS STEP-BY-STEP GUIDE:

1. Decide if you want to restrict background sampling to: i) a buffered minimum convex polygon based on observation localities, ii) a radial distance from all occurrence points or an intermediate between those two iii) using the tool: *Sample by Buffered Local Adaptive Convex-Hull*.
2. Double-click the corresponding tool 'SDM Tools →2. MaxEnt Tools → Background Selection via Bias Files→ Background Selection: Sample by Buffered MCP' or 'Background Selection: Sample by Distance from Obs. Pts.' or 'Background Selection: Sample by Buffered Local Adaptive Convex-Hull'
3. Continue to tool interface instructions (Buffered MCP below)

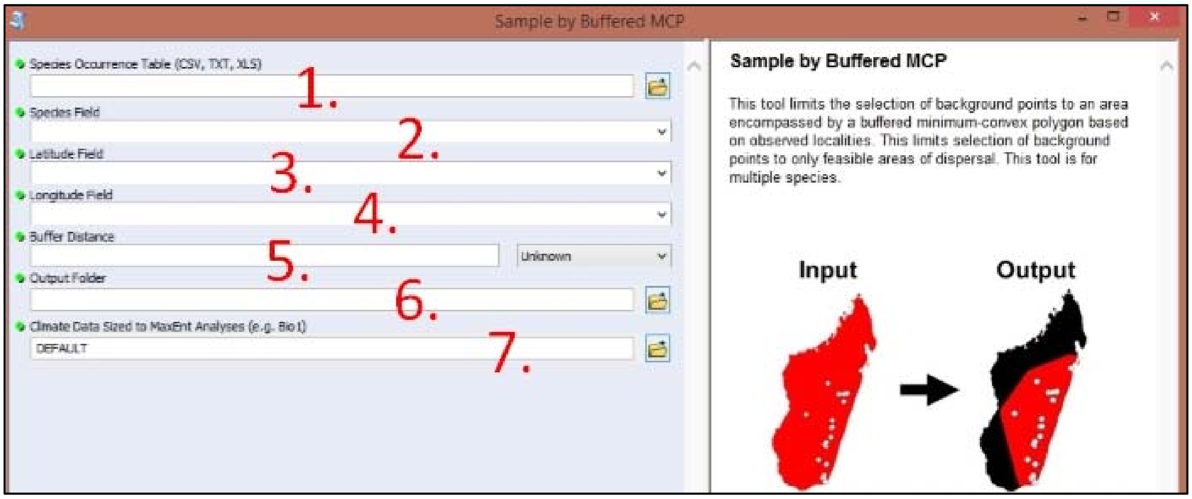

***Background Selection: Sample by Buffered MCP* tool interface**

SDMTOOLBOX STEP-BY-STEP GUIDE:

1. CSV file of species occurrences (from previous steps)
2. Field corresponding to species identity
3. File field corresponding to longitude
4. File field corresponding to latitude
5. The distance outside of minimum-convex-polygon included in background selection.
6. Select output folder location. This should be a new empty folder. If not empty this can cause the analysis to fail, particularly if temporary files from a previous analysis were not properly removed (e.g. this can happen if another SDMtoolbox analysis is terminated early).
7. Climate data sized to extent of MaxEnt Modeling. Here use the 'Bio_1.asc' layer. Select one of your climate files sized to your modeling extent (e.g. Bio1.asc). This file will be used to match the bias file to proper extent and resolution (no change will be made to this file).

Model calibration and validation

5. Spatial Jackknifing and independent tests of parameters

Why use SDMtoolbox for MaxEnt modeling:

I. Spatial Jackknifing

Spatial jackknifing (or geographically structured k-fold cross-validation) tests evaluation performance of spatially segregated spatially independent localities. SDMtoolbox automatically generates all the GIS files necessary to spatially jackknife your MaxEnt Models. The script splits the landscape into 3-5 regions based on spatial clustering of occurrence points (e.g. if 3: A,B,C). Models are calibrated with k-1 spatial groups and then evaluated with the withheld group. For example if k=3, models would be run with following three subgroups:

1. Model is calibrated with localities and background points from region AB and then evaluated with points from region C
2. Model is calibrated with localities and background points from region AC and then evaluated with points from region B
3. Model is calibrated with localities and background points from region BC and then evaluated with points from region A

II. Independent Tests of Model Feature Classes and Regularization Parameters

Equally important, this tool allows for testing different combinations of five model feature class types (FC) and regularization multiplier(s) (RM) to optimize your MaxEnt model performance. For example, if a RM was input (here 5), this tool kit would run MaxEnt models on the following parameters for each species:

1. RM: 5 & FC: Linear, 2. RM: 5 & FC: Linear and Quadratic, 3. RM: 5 & FC: Hinge, 4. RM: 5 & FC: Linear, Quadratic and Hinge, 5. RM: 5 & FC: Linear, Quadratic, Hinge, Product and Threshold

III. Automatic Model Selection

Finally, the script chooses the best model by evaluating each model's: 1. omission rates (OR)*,2. AUC**, and 3. model feature class complexity. It does this in order, choosing the model with the lowest omission rates on the test data. If many models have the identical low OR, then it selects the model with the highest AUC. Lastly if several models have the same low OR and high AUC, it will choose the model with simplest feature class parameters in the following order: 1. linear; 2. linear and quadratic; 3. hinge; 4. linear, quadratic, and hinge; and 5. linear, quadratic, hinge, product, and threshold.

Once the best model is selected, SDMtoolbox will run the final model using all the occurrence points. If desired, at this stage models will be projected into other climates, environmental variables will be jackknifed to measure importance, and response curves will be created.

*For each iteration, OR is weighted by the number of points in the evaluation subgroup. This is necessary because spatial groups may not have identical number of points. The weighing gives equal contribution to all points included in model evaluation.

**AUC is calculated from the total study area in the input bias file (if k=3, then all groups: ABC)

For info and justification of these methods see FAQ and then end of this document.

Citations: Boria, R. A., L. E. Olson, S. M. Goodman, and R. P. Anderson. 2014. Spatial filtering to reduce sampling bias can improve the performance of ecological niche models. Ecological Modelling, 275:73-77.
-Radosavljevic, A. and R. P. Anderson. 2014. Making better Maxent models of species distributions: complexity, overfitting, and evaluation. Journal of Biogeography. 41:629-643
-Shcheglovitova, M. and R. P. Anderson. 2013. Estimating optimal complexity for ecological niche models: a jackknife approach for species with small sample sizes. Ecological Modelling, 269:9-17.

Tool: Run MaxEnt: Spatially Jackknife

ArcGIS Step-by-Step Guide:

1. Double-click the 'SDM Tools →2. MaxEnt Tools → Modeling in MaxEnt → Run MaxEnt: Spatially Jackknife' tool
2. Continue to tool interface instructions (below)

SDMtoolbox Step-by-Step Guide:

IMPORTANT NOTE 1: none of the input and output file names/file paths can have spaces in them. If there are any spaces, the output batch scripts will likely fail to work properly.

IMPORTANT NOTE 2: Upon first use of this tool, due to its unique syntax, you need to specify the location of the menu file. If you do not due this, the menu presented by ArcGIS will not make complete sense. For a detailed overview of how to do this (it will take 30 seconds to do), go to: http://www.sdmtoolbox.org/menu-fix-spatial-jackknife

1. Folder with the MaxEnt program. Downloadable from: https://biodiversityinformatics.amnh.org/open_source/maxent/
2. CSV file output from spatial rarefied occurrence data (output from step 3D)
3. CSV field corresponding to species identity
4. CSV field corresponding to longitude
5. CSV field corresponding to latitude
6. Folder with clipped ASCII climate data from step 3C.
7. Select environmental layers from this folder that depict categorical data.
8. Select environmental layers to exclude from analyses (not list in output from step 2).
9. Folder containing bias files from step 4.
10. Output folder for GIS, python scripts and MaxEnt batch files. Output will include all GIS files necessary to run models.
11. For spatial jackknifing this format output will be Logistic, regardless of selection
12. For spatial jackknifing this format output will be ASCII, regardless of selection

13. Create graphs showing how predicted relative probability of occurrence depends on the value of each environmental layer
14. Create an image of each output model
15. Measure variable importance by jackknifing the variables. Each variable is excluded in turn and a model created with the remaining variables. Then a model is created using each variable in isolation.
16. This will skip the model if an output exists.
17. This will suppress any warnings encountered during modeling. All warnings are always written to the log files.
18. This is the breadth of the model. A higher number gives a more spread-out distribution. Input many values separated by semi-colons.

    For example:

    0.5; 1; 1.5; 2; 2.5; 3; 4; 5

    The default value is 1.

    Remember the more values input will produce more SDMs created and require more computation time. For each regularization multiplier (RM), 15 models will be run (5 feature class groups and 3-5 spatial jackknife groups, 5x3=15 to 5x5=25). This number is multiplied for each replicate and each species modeled. Thus, if you have 2 species, 5 RMs and 2 replicates; this would result in 300 or 500 models run for 3 and 5 spatial jackknife groups, respectively (2 species *2 replicates* 5 RM * 15-25 models per run)
19. Apply a threshold to make binary model. This will generated a binary

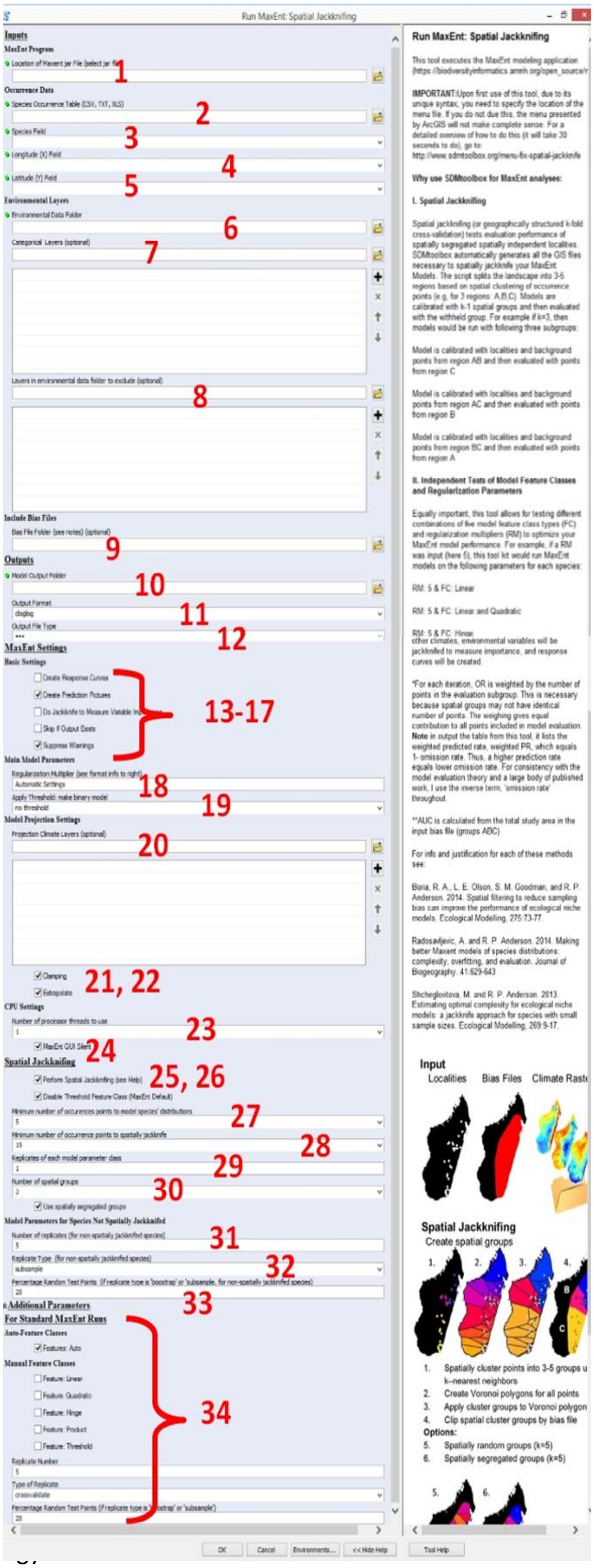

model in addition to the continuous model. In none is supplied, SDMtoolbox will use '10 percent minimum training presence' to calculated omission rates. If you prefer another threshold, please select it here.

20. Projection Climate Layers. Folders containing environmental data for projecting the MaxEnt models (often are future are past climates). To select multiple folders at once hold to shift. The layers MUST match the input environment layers, e.g. if Bio27 is used to build the model then the projection folder must contain an analog variable and have the identical name. Here resolution and spatial extent do not need to match input environmental layers.
21. Apply clamping when projecting
22. If checked, will predict areas of climate space outside of limits encountered during training
23. Number of CPUs to use for modeling
24. This will *not* display the MaxEnt GUI when running models- preferred
25. Check this box to perform all the analyses described into the information window. If not checked this will run the modeling as if executed from the MaxEnt GUI (no spatial jackknifing or independent evaluation of RV or feature classes).
26. This disables the use of the threshold feature class in the fifth group of feature class comparisons. This is the default setting for the latest version of MaxEnt.
27. This is the minimum number of points to execute spatial jackknifing. If *below* this value, the models will be trained and evaluated using either cross-validation, bootstrapping or sub-sampling (as specified below in steps: 30-32). Each non-spatially jackknifed group is optimized with independent tests of different combinations of the five model feature class types (FC) and input regularization multiplier (RM) values.
28. Replicates of each model parameter class in spatial jackknife runs
29. Number of groups to subdivide the landscape into. Higher the number the more models run, but also the more training points included in each model run.
30. If selected:

    Groups will be spatially segregated and numbers of occurrences within groups may not be equal. This analysis is more focused on natural spatial groups. This method is best if projecting models into other climates (i.e. current or past) and is particularly useful for training and evaluating model performance in potentially non-analogous climates.

    If not selected:

    Spatial jackknife groups will be spatially randomly and numbers of occurrences within groups will be equal (+/- 1, due to unequal group sizes for some combinations of occurrences records and group number). This method is best if not projecting models into other climates.
31. Replicates of each model parameter class for species with too few points to spatially jackknife
32. Replicate Type. If replicates are >1, then multiple runs are performed by this type:

    Crossvalidate: MaxEnt makes k number of folds of your occurrence data to train and test the data. Here you are not be able to tell MaxEnt how many replicates you would like to run or the percentage of occurrence data you would like withheld for model validation (test occurrences). Optimal if you have a large number of species occurrences.

Bootstrap: Replicates samples sets are chosen by sampling with replacement

Subsample: Replicate sample sets are chosen by removing the random test percentage (input in the following window) without replacement, the variables not included are then used for model evaluation

33. If replicate type is 'boostrap' or 'subsample, input the percentage of points used for subsampling.
34. Additional parameters not used in spatial jackknifing.
35. Run the tool.
36. After files are created, to execute models go to output folder. Click the batch file "Step1_Optimize_MaxEnt_Model_Parameters.bat" this will run all models and summary stats. Know this may take several hours, or even days (if you have many species and RV), to finish.
37. Once all the models from Step 1 are run, the "Step2_Run_Optimized_MaxEnt_Models.bat" will be populated with the best model parameters. Run this file to get final models.
38. To see model ranks for each species, open the corresponding folder and open the "species_name_SUMSTATS_RANKED_MODELS.csv." Here the best model is the first row. Feature number corresponds to feature class group with: 1=linear; 2=linear & quadratic; 3= hinge; 4=linear, quadratic, and hinge; and 5=linear, quadratic, hinge, product, and threshold.

Frequently asked questions and misconceptions regarding SDMtoolbox & Maxent:

Should I remove co-correlated variables?

As stated by Elith in her seminar paper, *A statistical explanation of Maxen*t:
"MaxEnt has an inbuilt method for regularization… that is reliable and known to perform well (Hastie et al., 2009). It implicitly deals with feature selection (relegating some coefficients to zero) and is unlikely to be improved - and more likely, degraded - by procedures that use other modelling methods to pre-select variables (e.g., Wollanet al., 2008). In particular, it is more stable in the face of correlated variables than stepwise regression, so there is less need to remove correlated variables (unless some of them are known to be ecologically irrelevant), or preprocess covariates by using PCA and selecting a few dominant axes"

If variables are highly co-correlated, the mathematical relationship of training values might be quite similar and the Maxent algorithm can potentially view them as equally good at characterizing that spatial patterns observed in training values. This does not result in overfitting. The two instances were co-correlated variables are of concern in Maxent models are: (1) the primary goal of the study aims to understand the explicit role of each environmental variable included in the model, and (2) projecting to other climates/landscapes with non-analogous climates. In the first case, given that several variables may equally explain the spatial patterns in data-training, the final 'important' variable will be selected randomly from that sub-group of highly correlated variables. This, then, gives the appearance that the others are not important. However, they are merely redundant and not needed. In the case of projecting to non-analogous climates, the addition of variables increases the likelihood of non-analogous climates (NAC). Further, if highly correlated variables are included that have NAC, this can cause the MESS plots (how NACs are assessed in Maxent) to display a NAC for each co-correlated layer, suggesting that inferences based on these areas should be regarded with extreme care (more so than would be with a single uncorrelated layer).

Why use SDMtoolbox?
How exactly does SDMtoolbox address model parameterization, discriminatory ability & overfitting?
Why is SDMtoolbox awesome?

Low overfitting and high discriminatory ability are two prime desired qualities of niche models (Lobo et al. 2008, Peterson et al. 2011, Warren and Seifert 2011). Discriminatory ability refers to the ability of a model to distinguish suitable from unsuitable areas, and is typically measured with the area under the curve of the receiver operating characteristic plot (AUC/ROC) (Peterson et al. 2011) as used by SDMtoolbox/Maxent. Overfitting is the tendency of a model to fit the random error (or any bias in the sample) rather than the true relationship between the calibration records and predictor variables. Often, overfit models predict the calibration data very well, but perform poorly on other data sets. Overfitting is typically assessed with the false negative rate, also called omission error rate (OR henceforth). With an appropriately selected threshold converting a continuous prediction into a binary one, ORs indicate the proportion of presences incorrectly classified as falling into unsuitable areas (basically because the prediction is too tightly fit to the conditions at calibration localities; Anderson et al. 2003). The top model output from SDMtoolbox is the one with the lowest OR. AUC values are then used to assess performance post-hoc, as an independent of model tuning- you should report both these values in your results section.

Model parameterization and corresponding overfitting can minimized by SDMtoolbox in the following ways:

1. Evaluating many regularization multipliers input into Maxent
2. Using spatial jackknifing for test and training datasets
3. Using independent tests of model feature classes
4. Carefully control of background selection using a bias files
5. Spatially rarefy/filter input data

As follows are explanations how these factors minimize model overfitting.

Evaluating many regularization multipliers input into Maxent

Text from (Radosavljevic & Anderson 2014).

"Maxent limits model complexity – and, hence, protects against overfitting – by regularization: a penalty for each term included in the model and for higher weights given to a term (Phillips et al., 2006; Anderson & Gonzalez, 2011). This penalty occurs in the form of a beta regularization parameter specific to each feature class (see the 'lasso' for generalized linear and generalized additive models; Phillips et al., 2006). Current releases of Maxent implement a regularization multiplier, a user-specified coefficient that is applied to the value of the respective beta parameter of each feature class, altering the overall level of regularization rather than changing the beta parameters individually. "

These authors reported that regularization multipliers as high as 2.0 to 4.0 were necessary to reduce overfitting resulting from lower levels. Qualitative assessments of the geographical predictions reiterate this conclusion. I typically run 10 regularization multipliers (from 0.5 to 5, in increments of 0.5; Maxent's default is 1).

Using spatial jackknifing for test and training datasets

Spatial jackknifing (or geographically structured k-fold cross-validation) tests and evaluating performance of spatially segregated localities. Here, we used SDMtoobox to split the landscape into 3 regions based on Voronoi polygons and the spatial clustering of occurrence points. Models were calibrated with all permutations of the groups using occurrence points and background data from two spatial groups and then evaluated with the withheld group. Spatial jackknifing has demonstrated and clear advantages over random sampling of test/training occurrence data (as is common practice and the default setting in Maxent). In experiments on the effects of these two treatments, randomly partitioned occurrence datasets produced inflated estimates of performance and led to over-fit models (Radosavljevic & Anderson 2014). Under the spatial jackknifing approach, increasing the regularization multiplier did not sufficiently counteract the effects of the strong spatial bias in the localities used for model calibration (artificially inserted into their experimental approach; Radosavljevic & Anderson 2014). In contrast, the spatial jackknifing approach was shown to sidestep problems of the artificial spatial bias (and any corresponding environmental biases) and allow for detection of overfitting to environmental biases that differed among the spatial partitions (Phillips, 2008).

When done in conjunction with tuning experiments (adjusting regulation multipliers and feature classes, discussed in other itemized points), this approach can allow the selection of model settings likely to avoid overfitting to noise as well as to the spatial biases in occurrence data and corresponding environmental biases (Radosavljevic & Anderson 2014).

Using independent tests of model feature classes
From Shcheglovitova & Anderson 2013:
"Feature class determines the kinds of constraints allowed in a model. A feature is a function of an environmental variable and in Maxent can be any single one or various combination of six classes: linear (L), quadratic (Q), prod- uct (P), threshold (T), hinge (H) or category indicator (C) (Phillips et al., 2006; Phillips and Dudík, 2008). The constraints placed on the model by features result in models of varying complexities. For instance, a model built with L features is less complex than one built with L and Q features. Hinge features model a piece-wise linear response to the environmental variable. This allows for parts of the response curve to be defined by a linear relationship while other parts can be defined by a more complex, non-linear relationship (Phillips and Dudík, 2008). Thus, L features represent a special (restrictive) case of H features and result in less complex models (Phillips and Dudík, 2008). Note that even if multiple feature classes are allowed for model-building, not all classes will necessarily be incorporated in the final model. The default Maxent setting for feature class, called "auto features," applies the class or classes estimated to be appropriate for the particular sample size of occurrence records, according to a previous extensive tuning experiment (Phillips and Dudík, 2008). Phillips and Dudík (2008) selected the following feature classes for continuous variables as default for the corresponding sample sizes: all feature classes for at least 80 occurrence records; L, Q and H for sample sizes 15 to 79; L and Q for 10 to 14 records; only L for below 10 records (Phillips and Dudík, 2008)."

While using a complex feature settings allows Maxent to produce a model that is more sensitive to details of a species' environmental tolerance, complex feature classes can lead to overfit models. Using SDMtoolbox, we repeat the methods of Shcheglovitova and Anderson (2013) to minimize model overfitting.

Carefully control background selection using a bias files
Bias files can control where background points are selected, and thereby avoid habitats greatly outside of a species' known occurrence. Background points are meant to be compared with presence data to help identify the environmental conditions under which a species can potentially occur. Typically, background points are selected within a large rectilinear area. Within such areas, environmentally suitable but uncolonized or biogeographicaly isolated habitat often exists. The selection of background points within these habitats increases commission errors (false positives). As a result, the 'best' performing model tends to be over-fitted because the selection criterion favors a model that fails to predict the species in the un-colonized climatically suitable habitat (Anderson & Raza 2010, Barbet-Massin et al. 2012). The likelihood that suitable unoccupied habitats are included in background sampling increases with distance from the realized range of the species. Thus, a larger study of spatial extent can lead to the selection of a higher proportion of less informative background points (Barbet-Massin et al. 2012). Such issues are ameliorated by being more selective in the choice of background points in Maxent (Barve et al. 2011; Merow et al. 2013). This can be done by using bias files in SDMtoolbox.

Spatially rarefy/filter input data
To perform well, most SDM methods require input-occurrence data to be spatially independent. However, researchers often introduce environmental biases into their SDMs from spatially autocorrelated occurrence points. It is important to eliminate spatial clusters of localities for model calibration and evaluation. When spatial clusters of localities exist, often models are over-fit towards

environmental biases (reducing the model's ability to predict spatially independent data) and model performance values are inflated (Veloz 2009; Hijimans et al. 2012; Boria et al. 2014). This can be done in SDMtoolbox using the spatial rarify toolbox.

Summary of some basic considerations when generating SDMs

Table modified from: Alvarado-Serrano, D. F. and Knowles, L. L. (2014), Ecological niche models in phylogeographic studies: applications, advances and precautions. Molecular Ecology Resources, 14: 233–248.   Please see paper for more details.

Assumptions that may affect SDMs	Specific considerations
**Data compilation occurrence records:**<br><br>Are species presences (and absence) records representative of the actual distribution?	▪effects of species' natural history<br>▪geographic/environmental bias<br>▪intraspecific variability<br>▪positional uncertainty<br>▪sample size<br>▪sampling bias (e.g. towards more accessible areas)<br>▪taxonomic accuracy (e.g. subspecies or races)<br>▪temporal coverage in relation to environmental data
**Data compilation Environmental variables:**<br><br>Do environmental variables accurately capture the association between species subsistence and the environment at the relevant scale?	▪data quality and biases<br>▪effect on species distribution (direct vs. indirect)<br>▪resolution in space and time<br>▪spatial autocorrelation<br>▪spatial extent<br>▪temporal coverage and stability<br>▪type (categorical vs. continuous)
**Model generation and calibration**<br><br>Is the modelling algorithm appropriate given the data available and research question?	▪algorithm assumptions<br>▪algorithm performance<br>▪under different scenarios<br>▪input data type (e.g. presences only vs. presence/absences)<br>▪output generated (e.g. presence/absence vs. continuous prediction)<br>▪sensitivity to model parameters
**Model generation and calibration**<br><br>Is the model appropriately calibrated for the data available and research question?	▪model complexity<br>▪model selection procedure<br>▪setting of model parameters<br>▪variable selection strategy
**Model validation**<br><br>Is validation performed on truly independent data and under appropriate settings?	▪assumptions/limitations of accuracy measurement<br>▪importance of use of multiple metrics<br>▪sensitivity to model parameters<br>▪threshold transformation of continuous predictions
**Model projection**	▪availability of validation data in projected regions<br>▪likelihood of niche shifts

Is the species environment relationship likely to be maintained in space and/or time?	▪model uncertainty<br>▪model transferability<br>▪risks of interpolation and extrapolation

References

Anderson R., Lew D., & Peterson A. (2003) Evaluating predictive models of species' distributions: criteria for selecting optimal models. Ecological modelling, 162, 211–232.

Anderson, R. P. & Raza, A. (2010) The effect of the extent of the study region on GIS models of species geographic distributions and estimates of niche evolution: preliminary tests with montane rodents (genus Nephelomys) in Venezuela. Journal of Biogeography, 37, 1378-1393.

Barbet-Massin, M., Jiguet, F., Albert, C. H. & Thuiller, W. (2012) Selecting pseudo-absences for species distribution models: how, where and how many? Methods in Ecology and Evolution, 3, 327–338.

Barve, N., Barve, V., Jiménez-Valverde, A., Lira-Noriega, A., Maher, S.P., Peterson, A.T., Soberón, J. & Villalobos, F. (2011) The crucial role of the accessible area in ecological niche modeling and species distribution modeling. Ecological Modelling, 222 (11), 1810-1819.

Merow, C., Smith, M.J., & Silander, J.A. (2013) A practical guide to MaxEnt for modeling species' distributions: what it does, and why inputs and settings matter. Ecography, 36, 1058–1069.

Phillips, S.J., Dudík, M., 2008. Modeling of species distributions with Maxent: new extensions and a comprehensive evaluation. Ecography 31, 161–175

Veloz S.D. (2009) Spatially autocorrelated sampling falsely inflates measures of accuracy for presence-only niche models. Journal of Biogeography, 36, 2290–2299.

Peterson A.T., Soberón J., Pearson R.G., Anderson R.P., Martínez-Meyer E., Nakamura M., & Araújo M.B. (2011) Ecological Niches and Geographic Distributions. Princeton Universtiy Press

Phillips S.J., Anderson R.P., & Schapire R. (2006) Maximum entropy modeling of species geographic distributions. Ecological Modelling, 190, 231–259.

Hijmans R.J. (2012) Cross-validation of species distribution models: removing spatial sorting bias and calibration with a null model. Ecology, 93, 679–688.

Shcheglovitova, M. and R. P. Anderson. 2013. Estimating optimal complexity for ecological niche models: a jackknife approach for species with small sample sizes. Ecological Modelling, 269:9-17.

Lobo J.M., Jiménez-Valverde A., & Real R. (2008) AUC: a misleading measure of the performance of predictive distribution models. Global Ecology and Biogeography, 17, 145–151.

Boria, R. A., L. E. Olson, S. M. Goodman, and R. P. Anderson. 2014. Spatial filtering to reduce sampling bias can improve the performance of ecological niche models. Ecological Modelling, 275:73-77.

Radosavljevic, A. and R. P. Anderson. 2014. Making better Maxent models of species distributions: complexity, overfitting, and evaluation. Journal of Biogeography. 41:629-6

Warren D.L. & Seifert S.N. (2011) Ecological niche modeling in Maxent: the importance of model complexity and the performance of model selection criteria. Ecological Applications, 21, 335–342

Final remarks

Great job—only a small proportion of all published papers using SDMs/ENMs address the following best practices of modeling:

1. Used species-specific regional background sampling
2. Spatially rarefied occurrence data
3. Spatially jackknifed SDMs to calibrate model parameters
4. Independently evaluated feature class parameters and regularization multiplier(s)
5. Reduced correlation of input climate variables for interpreting influence on model (optional here)

Now consider the following SDMtoolbox tools for further analyses:

If projecting models into future or past climates:

1. Limit Dispersal in Future SDMs
2. Overprediction Correction: Clip Models by Buffered MCPs
3. Distribution Changes Between Binary SDMs:
    a. Centroid Changes (Lines)
    b. Distribution Changes Between Binary SDMs

To create betters models:

4. Correcting Latitudinal Background Selection Biases
5. Gaussian kernel density of sampling localities

To assess landscape connectivity:

6. Among all sites or between shared haplotypes
7. Create friction layers

To measure spatial biodiversity patterns of many SDMs:

8. Calculate species richness and endemism (weighted endemism & corrected weighted endemism)

Now go publish your results!
