# Supplementary material for: SDMtoolbox 2.0: the next generation Python-based GIS toolkit for landscape genetic, biogeographic and species distribution model analyses
Source: PeerJ. 2017 Dec 5;5:e4095. doi: 10.7717/peerj.4095 (PMC5721907; doi:10.7717/peerj.4095)
Supplement: Supplemental Information 3 [file peerj-05-4095-s003.pdf]

Getting Started

Downloading

The latest version of the toolbox is available for download at: www.sdmtoolbox.org. This software requires ArcMap 10.1-10.5 with an active Spatial Analyst license (www.ESRI.com). This toolbox is programed specifically for ArcMap 10.1 (and above) and due to a series of improvements in this version, it is not backwards compatible with older releases of ArcMap (*i.e.* ArcMap 9.2). This software consists of an ArcGIS toolbox and associated python scripts.

Installation

1. Download, unzip 'SDMtoolbox.zip' and place all files in a folder on your harddrive where you want the toolbox to be stored (e.g …\Documents\ArcGISToolboxes)

2. Close all ArcGIS programs

3. Open **ArcCatalog 10.X** (a program in the ESRI ArcGIS programs, see icon below)

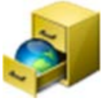

Above: ArcCatalog Icon

4. Once open, activate the ArcToolbox window (if not already visible) by clicking the red box below

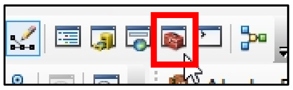

5. Then right-click ArcToolbox Window (see image below)→ select 'Add Toolbox.' Then go to location of downloaded toolbox and select 'SDMToolbox v2.0.tbx'

6. The toolbox should appear inside the ArcToolbox (see highlighted toolbox to right). If there, now the toolbox is almost installed.

7. To finish installation simply close the ArcCatalog program.

8. Now start ArcMap and begin using the SDMtoobox!

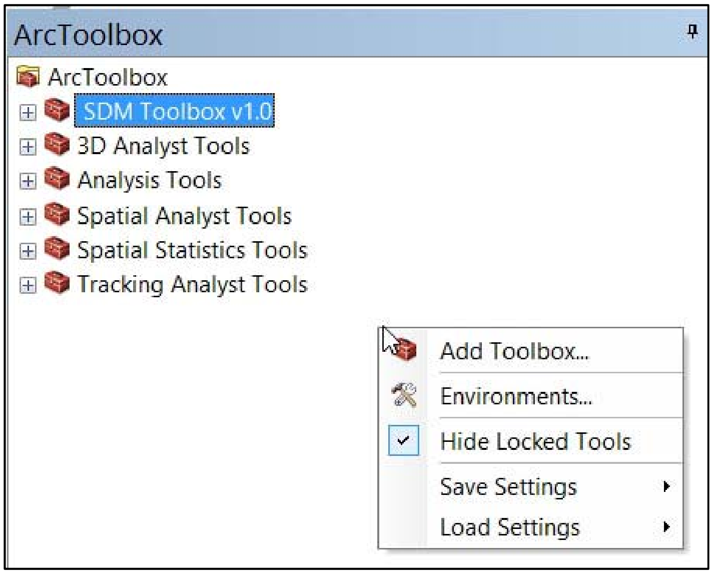

How to update or uninstall SDMtoolbox

1. Close all ArcGIS programs

2. Open **ArcCatalog 10** (a program in the ESRI ArcGIS programs, see icon below)

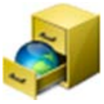

Above: ArcCatalog Icon

3. Once open, activate the ArcToolbox window (if not already visible) by clicking the red box below

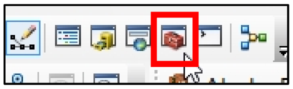

4. Then right-click SDMtoollbox in ArcToolbox Window (see Image to right) and select ‘Remove’.

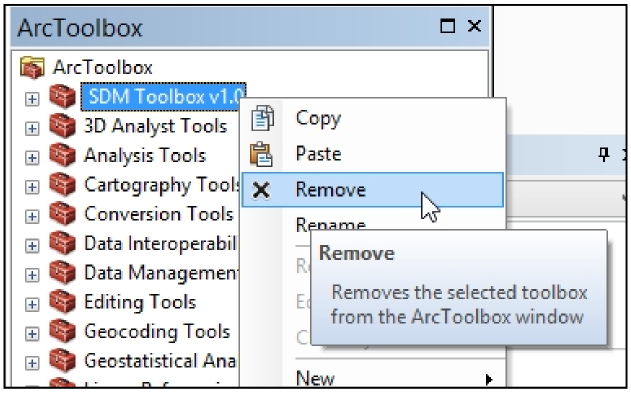

5. If only uninstalling, then you are done. If installing a new version of SDMtoolbox then right-click ArcToolbox Window (see image below)→ select ‘Add Toolbox.’ Then go to location of downloaded toolbox and select ‘SDMToolbox v1.X.tbx’

6. The toolbox should appear inside the ArcToolbox (see highlighted toolbox to right). If there, now the toolbox is almost installed.

7. To finish installation simply close the ArcCatalog program.

8. Now start ArcMap and begin using the SDMtoobox!

A First Run

Many things will cause the SDMtoolbox to *not* run that have *nothing* to do with the toolbox itself. Upon first use, the following steps should be performed:

1. **Open ArcMap10.X and activate the ArcToolbox window (if not already visible). Bottom image on this page is the Arctoolbox window. If not visible, click toolbox icon (see image below).**

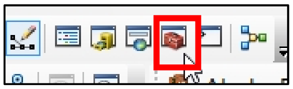

2. **Ensure Spatial Analyst is Enabled in ArcMap**

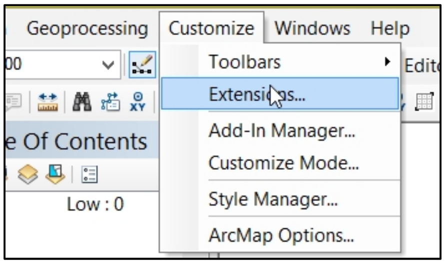

A. Go to: Customize→ Extensions

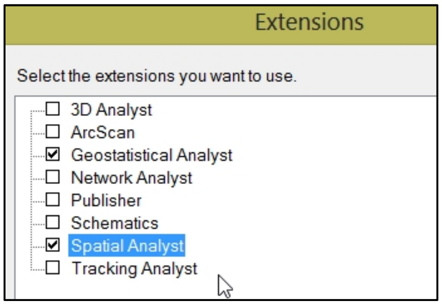

B. Check the box next to: *Spatial Analyst* (and *Geostatistical Analyst* if available)

3. **Install all relevant ArcMap 10.X Patches and Service Packs.**

   The base release of ArcMap 10.1/2 has some serious bugs that cause many analyses to fail. The only way to fix these bugs is the install all relevant patches and service packs.

   A. To search for these go to:

   http://support.esri.com/en/downloads/patches-servicepacks/list?productid=160&productVersions=10.1&categoryTypes=5&categoryTypes=8

   Or: http://support.esri.com/en/downloads/patches-servicepacks

   B. Or use the ESRI's 'Patch Finder for Windows' to identify patches, then search for suggested patches/service packs (http://downloads2.esri.com/Support/downloads/other_/PatchFinder.exe)

4. **Enable 'Tool Help' and resize windows.**

   Open the 'Basic Tools →Raster Tools → 2a. Raster to ASCII (Folder)' tool. Double-Click the tool (see image to right)

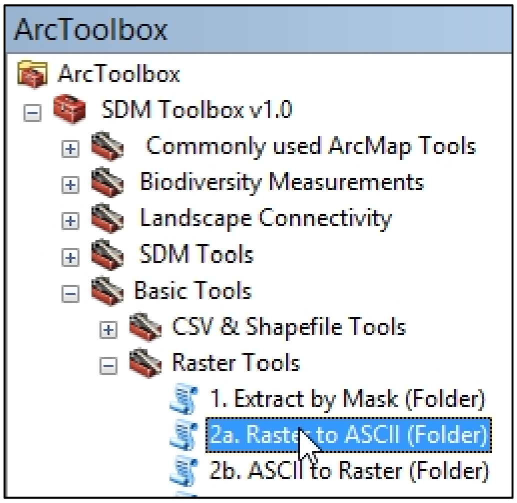

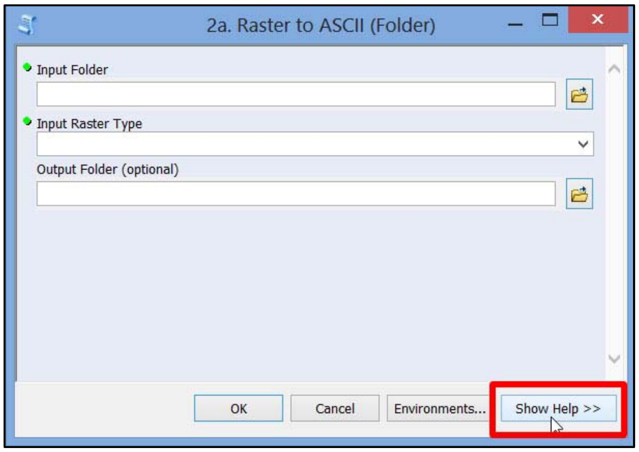

A. In the newly opened tool click 'Show Help' (red box above)

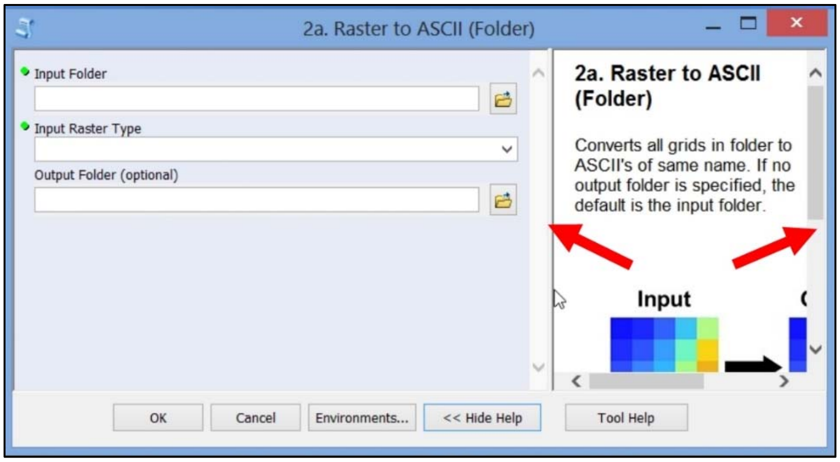

B. Resize the window by dragging the two windows (depicted by red arrows) so that the final tool window is as below. Note the SDMtoolbox Icon should be fully visible. Resizing the help window to this size will ensure that all diagrams are completely visible.

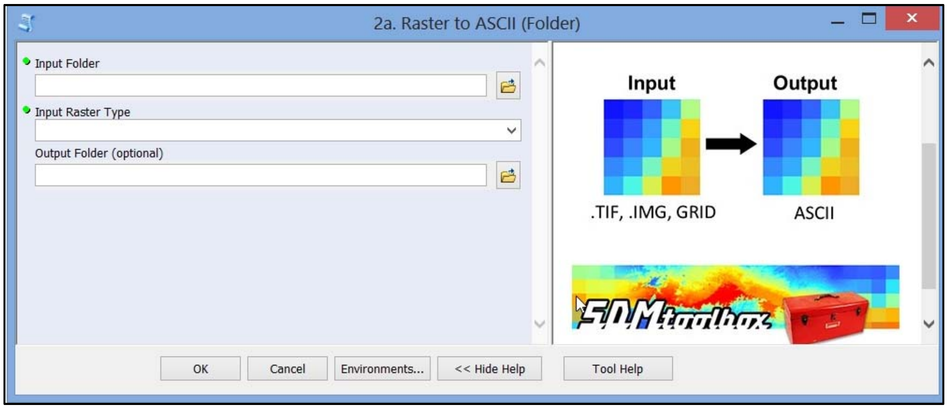

A note on map projections

Most user reported errors have to do with map projections. As follows is a checklist of things you need to check if you have a script fail. In most cases your data should be projected, with the projection specified in the GIS file.

1. **If you change or define the map projection** after opening the map document. You NEED to save all projected files, then close the map document and **open a new map session**. The first file open in your new session needs to be one of the newly projected files—this defines the map datum. This is particularly important if you change the spatial units as the result of projection, e.g. decimal degrees to meters. If you do not do this, often SDMtoolbox scripts will fail or result in inaccurate results.

2. **If the tool requires a CSV of coordinates** AND your analyses are going to be **in another projection**, remember to convert input coordinates (or XYs) to the new, and same, datum prior to running tools or for use in species distribution modeling.

   For most, the quickest way to do this for CSV files (and other tables) is to:

   1) Import the CSV to a shape file (*Table & Shapefile Tools* → *CSV to shapefile* tool)
   2) Define input projection of shapefile and then project to the new projection (a. ArcGIS *define projection* and b. *project* tools)
   3) Add the new coordinates/XYs to Shapefile using the ArcGIS *Add XYs* tool
   4) Export shapefile table as CSV file (SDMtoolbox the *Table & Shapefile Tools* →*Shapefile to CSV* tool)

   If using an equal-areas projection and input data are WGS-1984 coordinates, try the SDMtoolbox tool that will do all the above steps:

   SDM tools → 2. MaxEnt Tools→Correcting Latitudinal Background Selection Biases→ Solution 2: Project Input Data to Equal-Area Projection (EAP)→ 1. CSV to EAP. MaxEnt format output

3. Check that ***all* your input data are projected and in the *same* projection**. This is the most common error. Often reference ASCII files are not projected at all.
4. Last, **visualize all GIS files** to make sure they are in fact properly projected (all maps should overlap etc.). Sometimes files say they are projected to the same projection, however in reality something was incorrect.

To check the projection of each GIS file

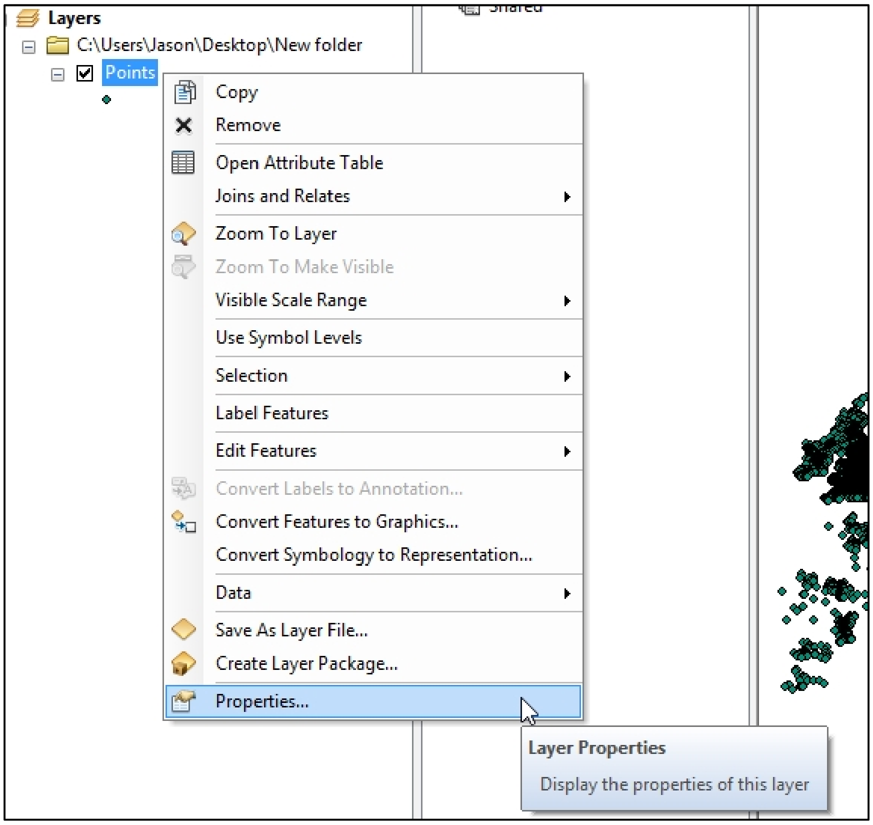

Import GIS files, then right click each file and select ‘Properties’

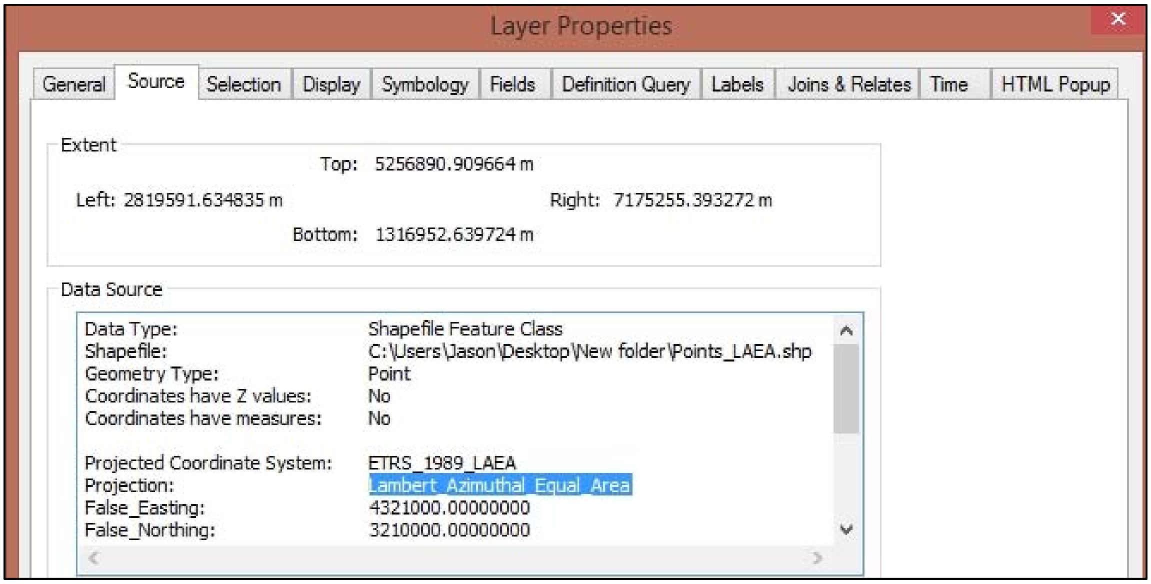

Select the ‘Source’ tab and page down until you see the ‘Projection’. Here the projection is Lamberts Azimuthal Equal Area

To accompany this guide I have provided example data available at www.sdmtoolbox.org. Download this, the latest version of the SDMtoolbox, and this guide before beginning.

This guide does not cover all the tools in the SDMtoolbox. However, as an overview, the guide covers tools from all the major groups. For many tools lacking a guide I have included example data to execute each analysis. The example data should be contained in a folder respective to the hierarchy of the toolbox. For example, for the tool ‘SDM toolbox: Biodiversity Measurements → Input: Point Data → Calculate Richness and Endemicity (WE and CWE)’, the example data are in the folder ‘…example_data\biodiversity_measurements\biodiversity_points’

Lastly, each tool is annotated and instructions should be contained within each tool’s help file from within ArcGIS. Follows are the major groups of the toolbox. The guide treats each one as a separate chapter.

The 10 commandments of SDMtoolbox 2.0

1. Thou shall not include spaces in path or file names
2. Thou shall avoid non-alphanumeric characters in file names, headings and table values
    - avoid: * : \ / < > | ” ? [ ] ; = + & £ $, etc
3. Thou shall have map projection defined
    - make sure all input data are properly projected and in the *same* projection (also see ‘A First Run‘ for details on this)
4. Thou shall limit table headings to 12 or less characters
    - Sometimes scripts use table headings, often ArcGIS reduces these name to 12 (or less) characters in analyses and confuses its self or input code that calls the full name
5. Thou shall not install an alternative version of Python
6. Thou shall have an appropriate ArcGIS license
    - For full functionality, ArcMap version **MUST** be Standard or Advance license level with Spatial Analyst Extension
7. Thou shall try provided example data corresponding to tool
    - If the example data work, but yours do not, there is a problem with your input parameters or files- compare your inputs to the provided inputs
8. Thou shall read SDMtoolbox error messages

- The red text is ArcMap's error text, SDMtoolbox often has its own more, specific error codes preceding (highlighted below)

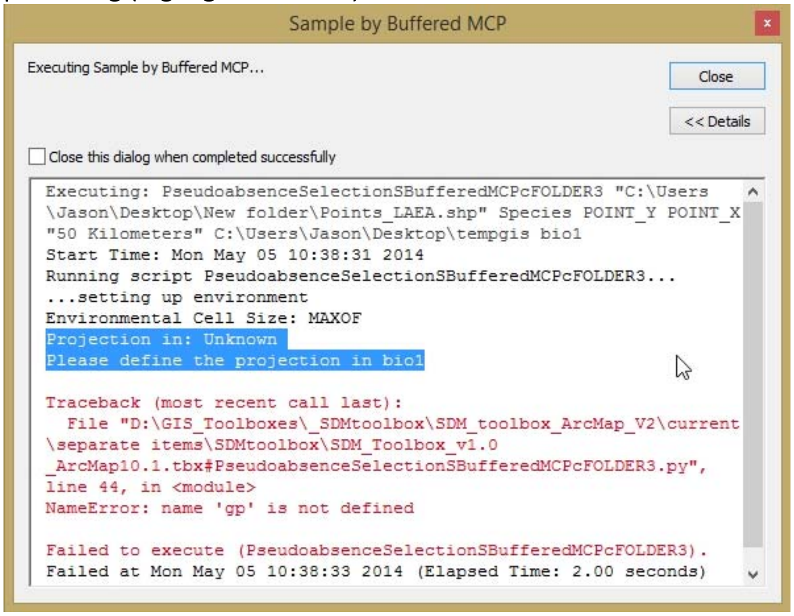

9. Thou shall remedy all these items before emailing SDMtoobox
10. When emailing SDMtoolbox, thou shall include detailed information regarding the problem(s), include screenshots of errors and information regarding input data
    - I usually cannot help you with out this information
