# Supplementary figures and images for: SDMtoolbox 2.0: the next generation Python-based GIS toolkit for landscape genetic, biogeographic and species distribution model analyses
Source: PeerJ. 2017 Dec 5;5:e4095. doi: 10.7717/peerj.4095 (PMC5721907; doi:10.7717/peerj.4095)

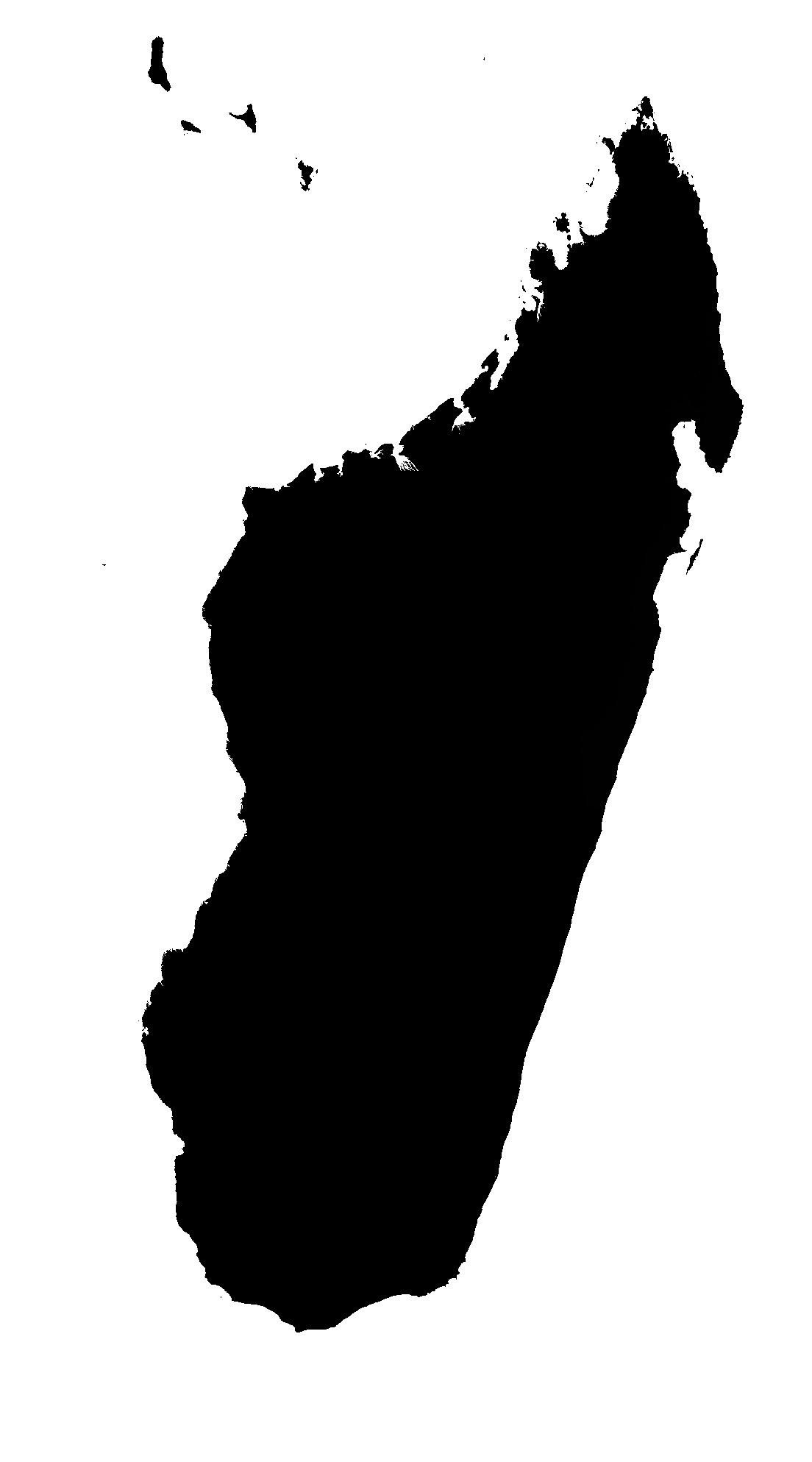

Supplement: Supplemental Information 4 [file peerj-05-4095-s004.zip › example_data/Biodiversity_measurements/biodiversity_binary_SDMs/Binary_SDMs/Uruloke_aragornii_prunned.tif]

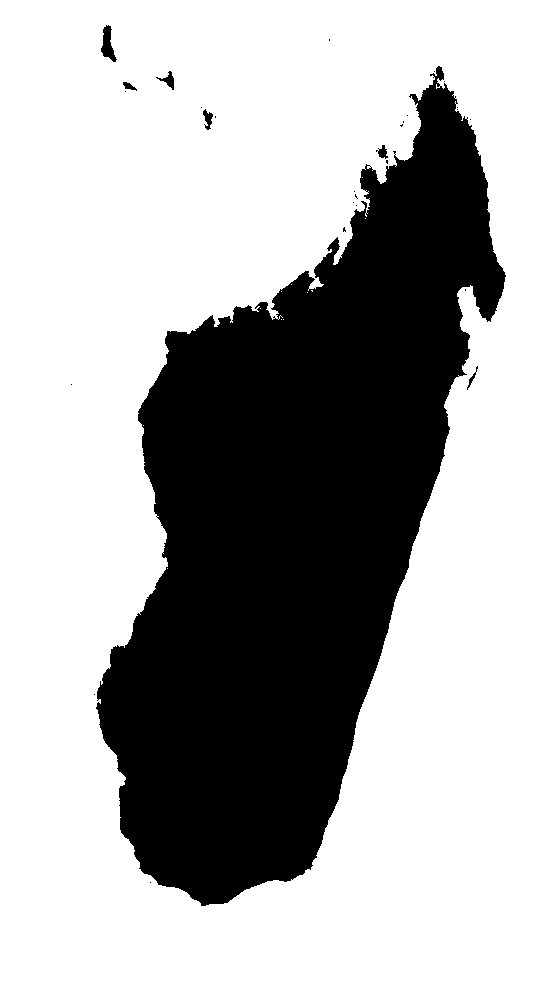

Supplement: Supplemental Information 4 [file peerj-05-4095-s004.zip › example_data/Biodiversity_measurements/biodiversity_binary_SDMs/Binary_SDMs/Uruloke_aragornii_prunned.tif.ovr]

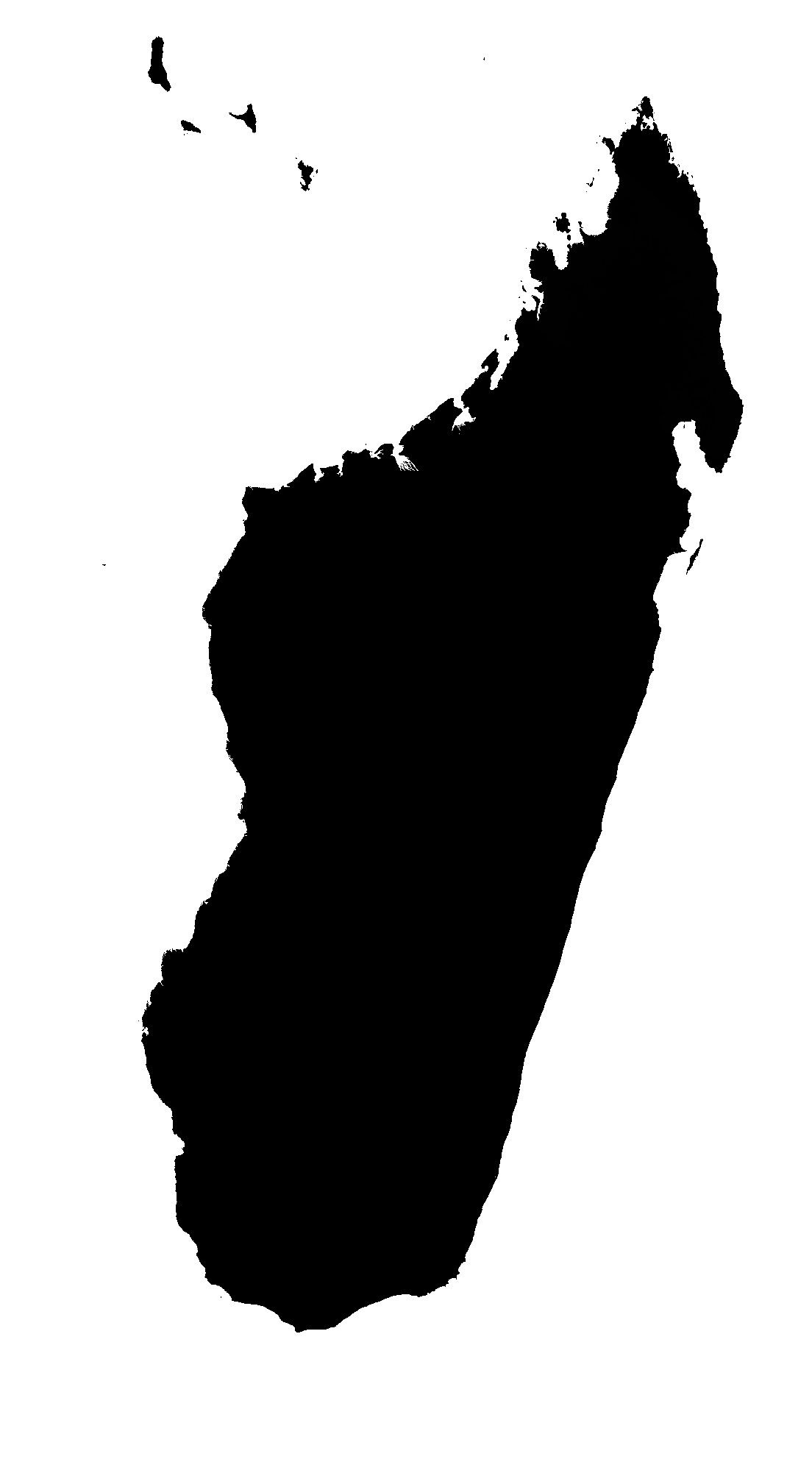

Supplement: Supplemental Information 4 [file peerj-05-4095-s004.zip › example_data/Biodiversity_measurements/biodiversity_binary_SDMs/Binary_SDMs/Uruloke_arwenae_prunned.tif]

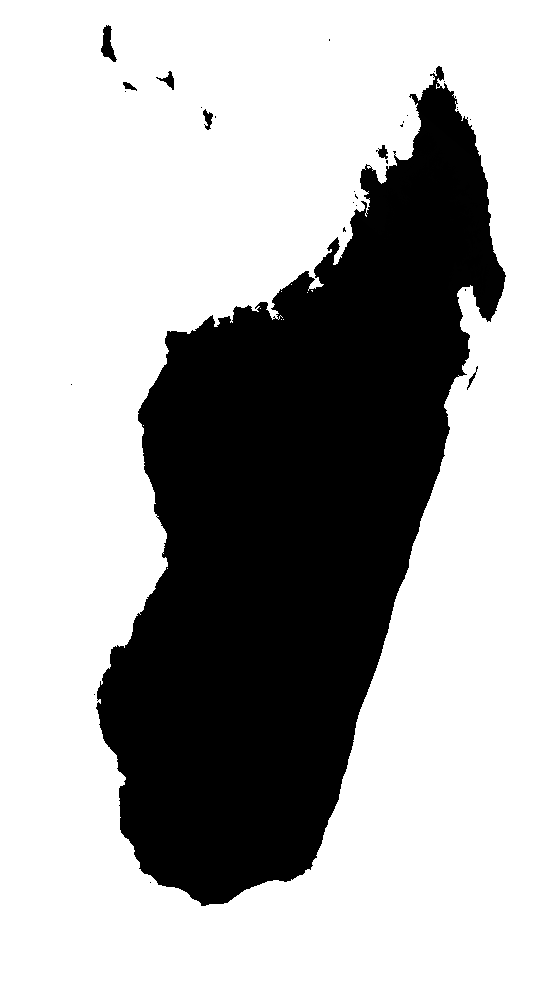

Supplement: Supplemental Information 4 [file peerj-05-4095-s004.zip › example_data/Biodiversity_measurements/biodiversity_binary_SDMs/Binary_SDMs/Uruloke_arwenae_prunned.tif.ovr]

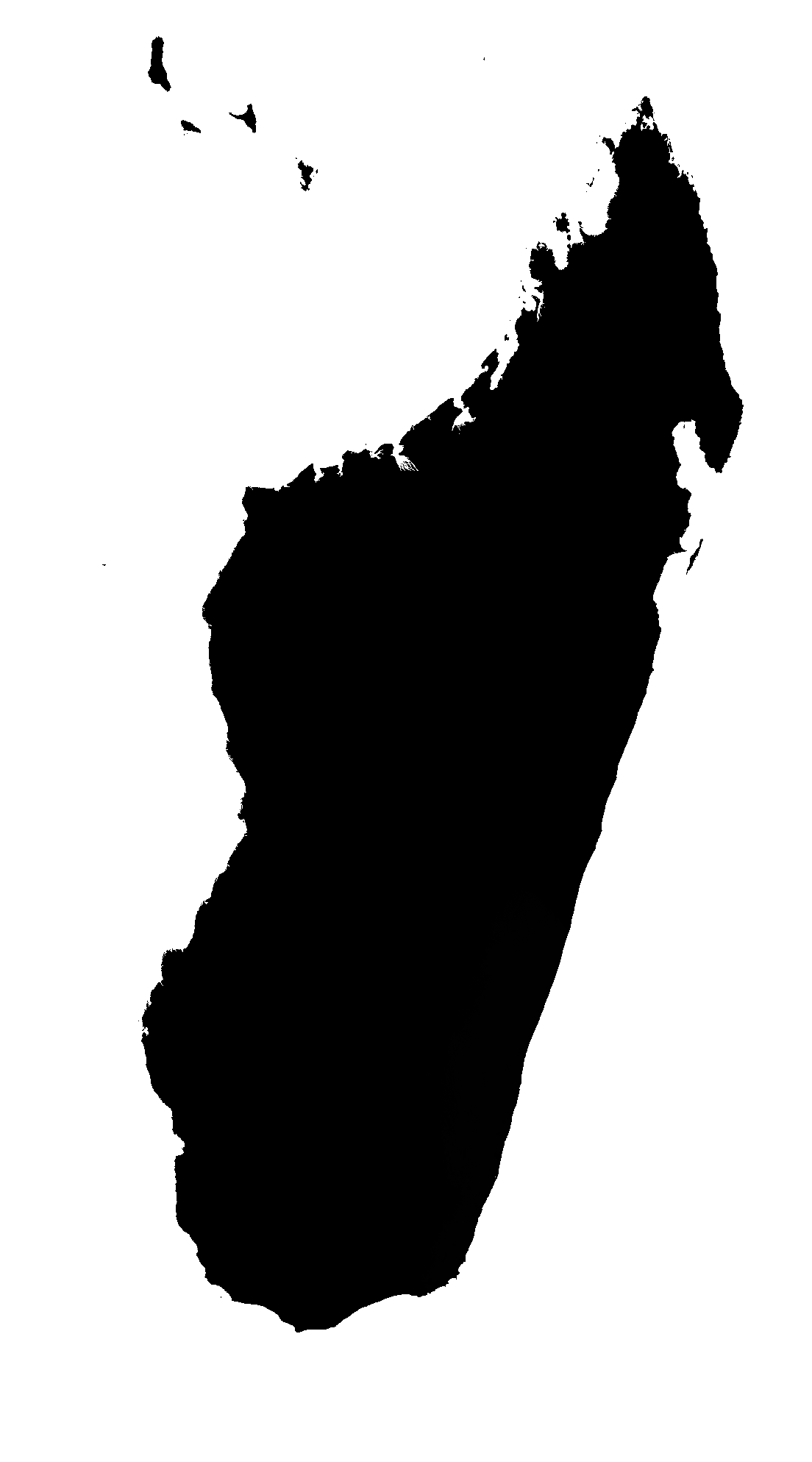

Supplement: Supplemental Information 4 [file peerj-05-4095-s004.zip › example_data/Biodiversity_measurements/biodiversity_binary_SDMs/Binary_SDMs/Uruloke_contortaentae_prunned.tif]

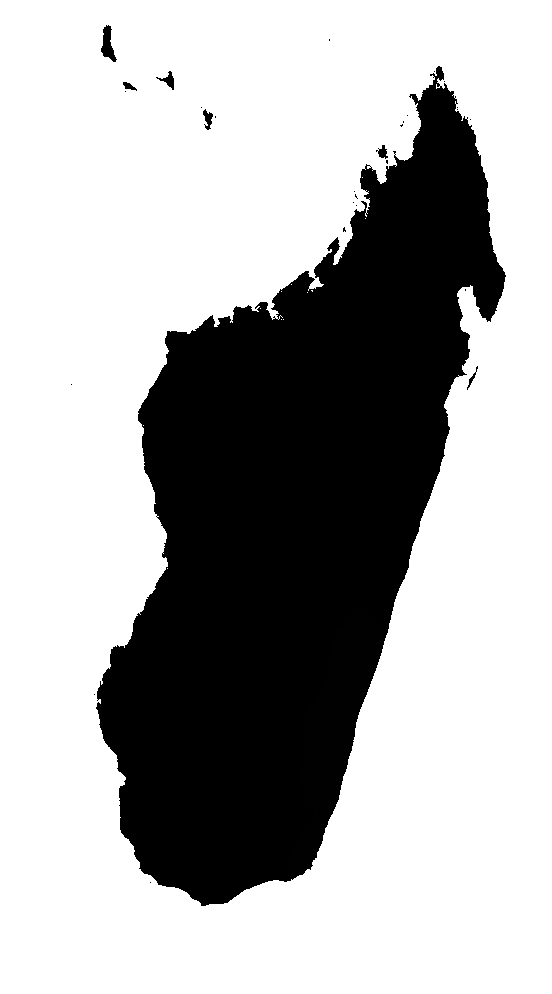

Supplement: Supplemental Information 4 [file peerj-05-4095-s004.zip › example_data/Biodiversity_measurements/biodiversity_binary_SDMs/Binary_SDMs/Uruloke_contortaentae_prunned.tif.ovr]

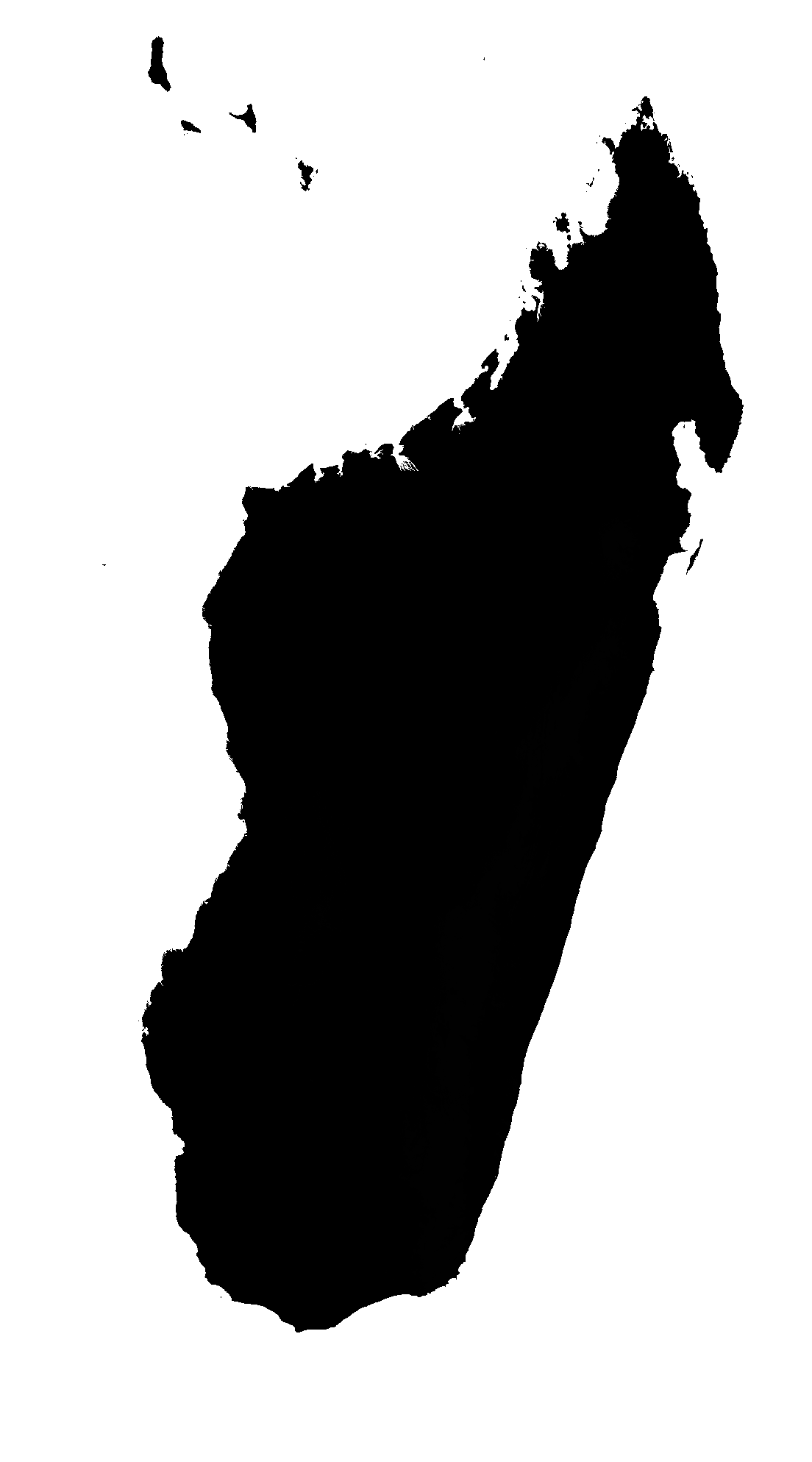

Supplement: Supplemental Information 4 [file peerj-05-4095-s004.zip › example_data/Biodiversity_measurements/biodiversity_binary_SDMs/Binary_SDMs/Uruloke_eowynae_prunned.tif]

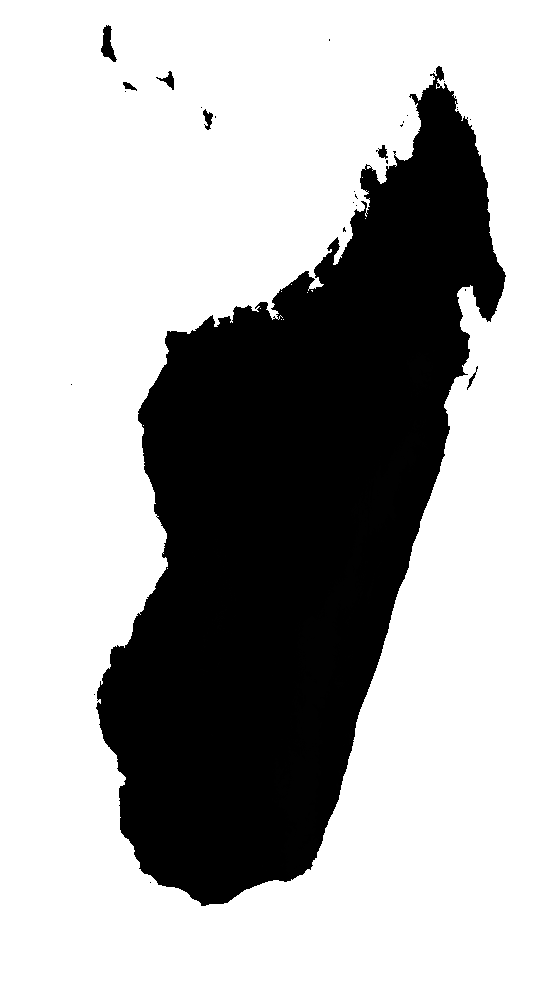

Supplement: Supplemental Information 4 [file peerj-05-4095-s004.zip › example_data/Biodiversity_measurements/biodiversity_binary_SDMs/Binary_SDMs/Uruloke_eowynae_prunned.tif.ovr]

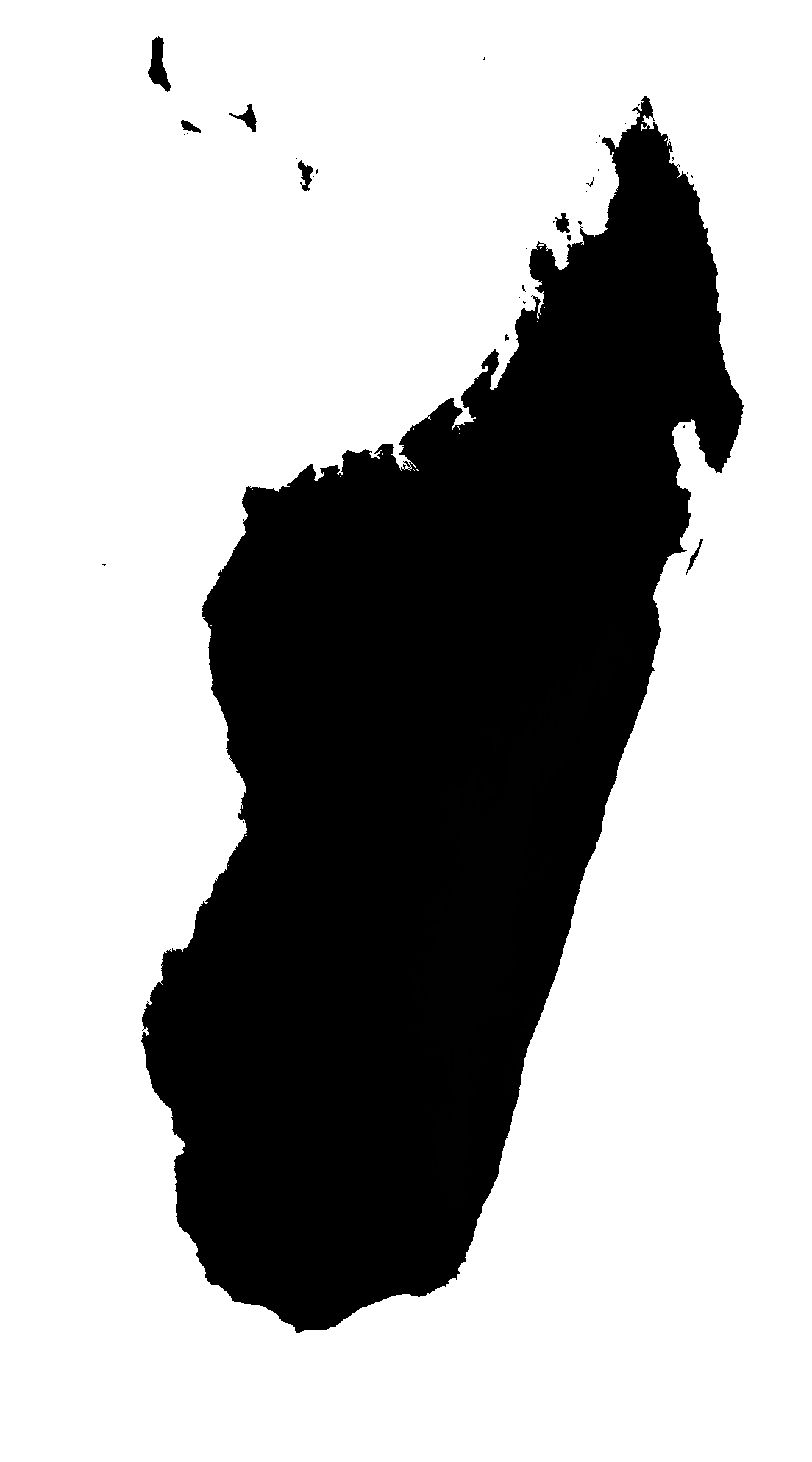

Supplement: Supplemental Information 4 [file peerj-05-4095-s004.zip › example_data/Biodiversity_measurements/biodiversity_binary_SDMs/Binary_SDMs/Uruloke_fangorcola_prunned.tif]

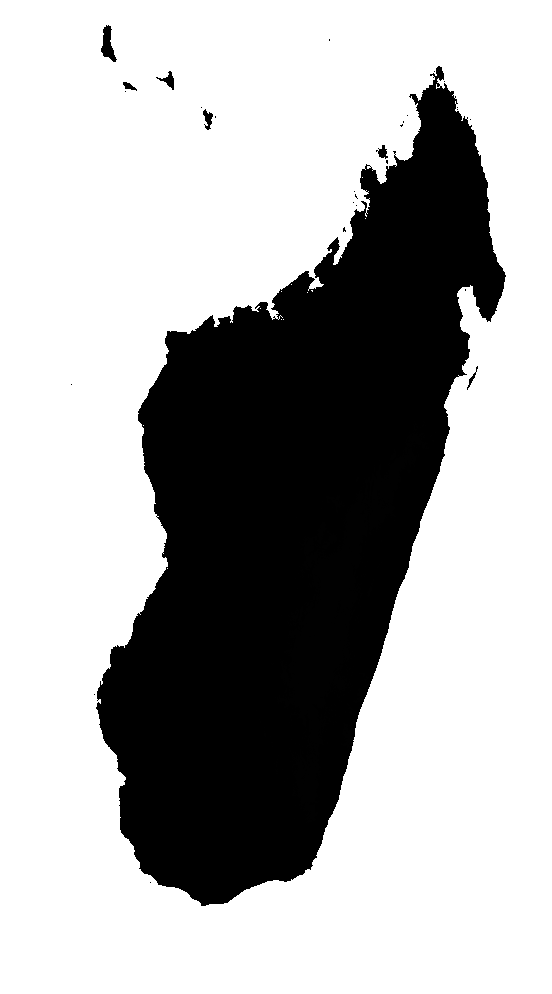

Supplement: Supplemental Information 4 [file peerj-05-4095-s004.zip › example_data/Biodiversity_measurements/biodiversity_binary_SDMs/Binary_SDMs/Uruloke_fangorcola_prunned.tif.ovr]

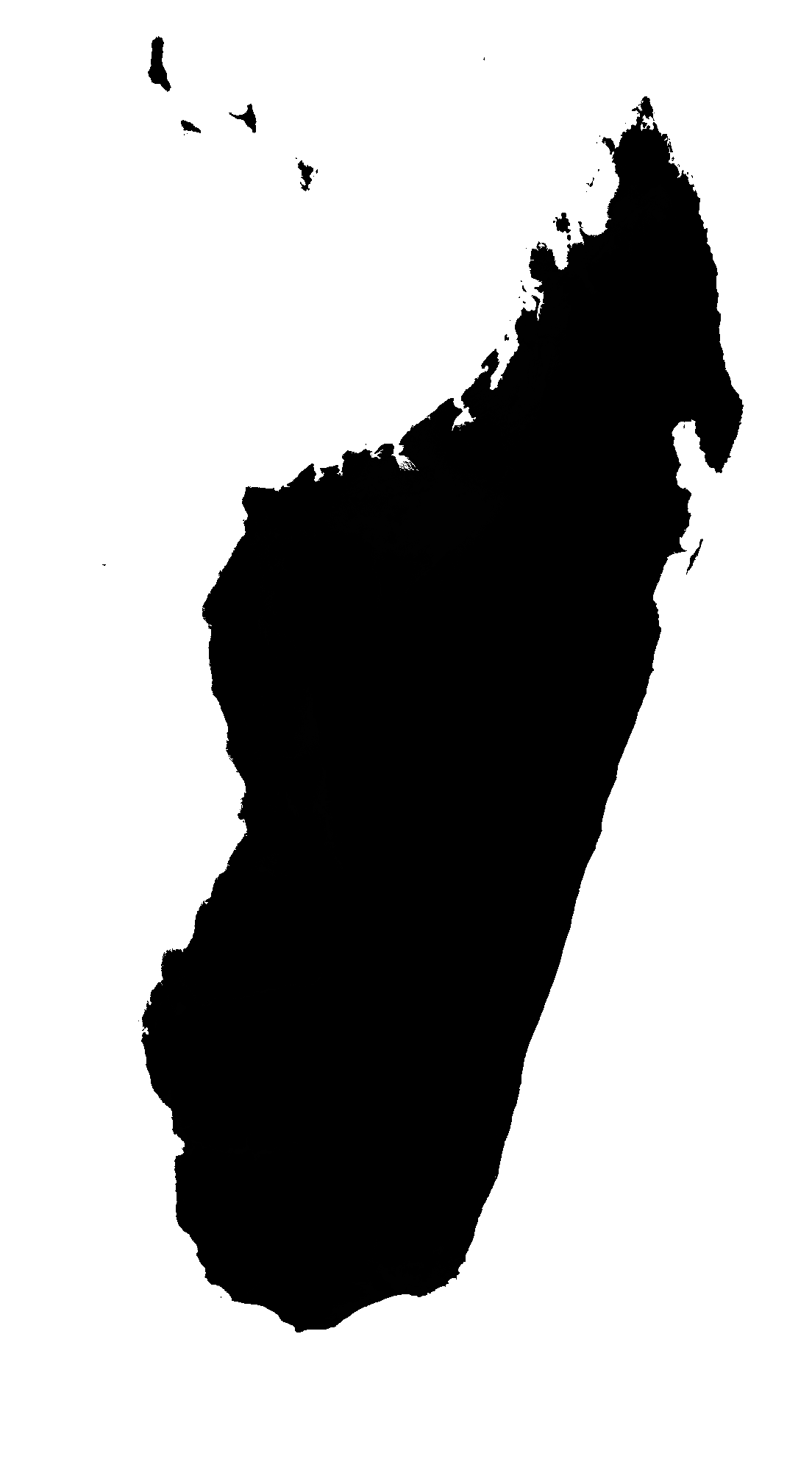

Supplement: Supplemental Information 4 [file peerj-05-4095-s004.zip › example_data/Biodiversity_measurements/biodiversity_binary_SDMs/Binary_SDMs/Uruloke_gimlia_prunned.tif]

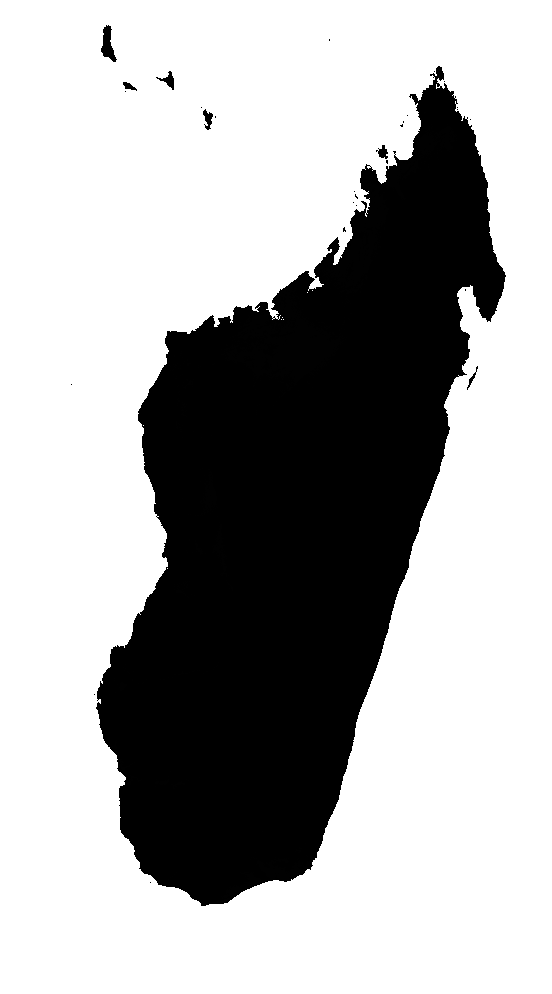

Supplement: Supplemental Information 4 [file peerj-05-4095-s004.zip › example_data/Biodiversity_measurements/biodiversity_binary_SDMs/Binary_SDMs/Uruloke_gimlia_prunned.tif.ovr]

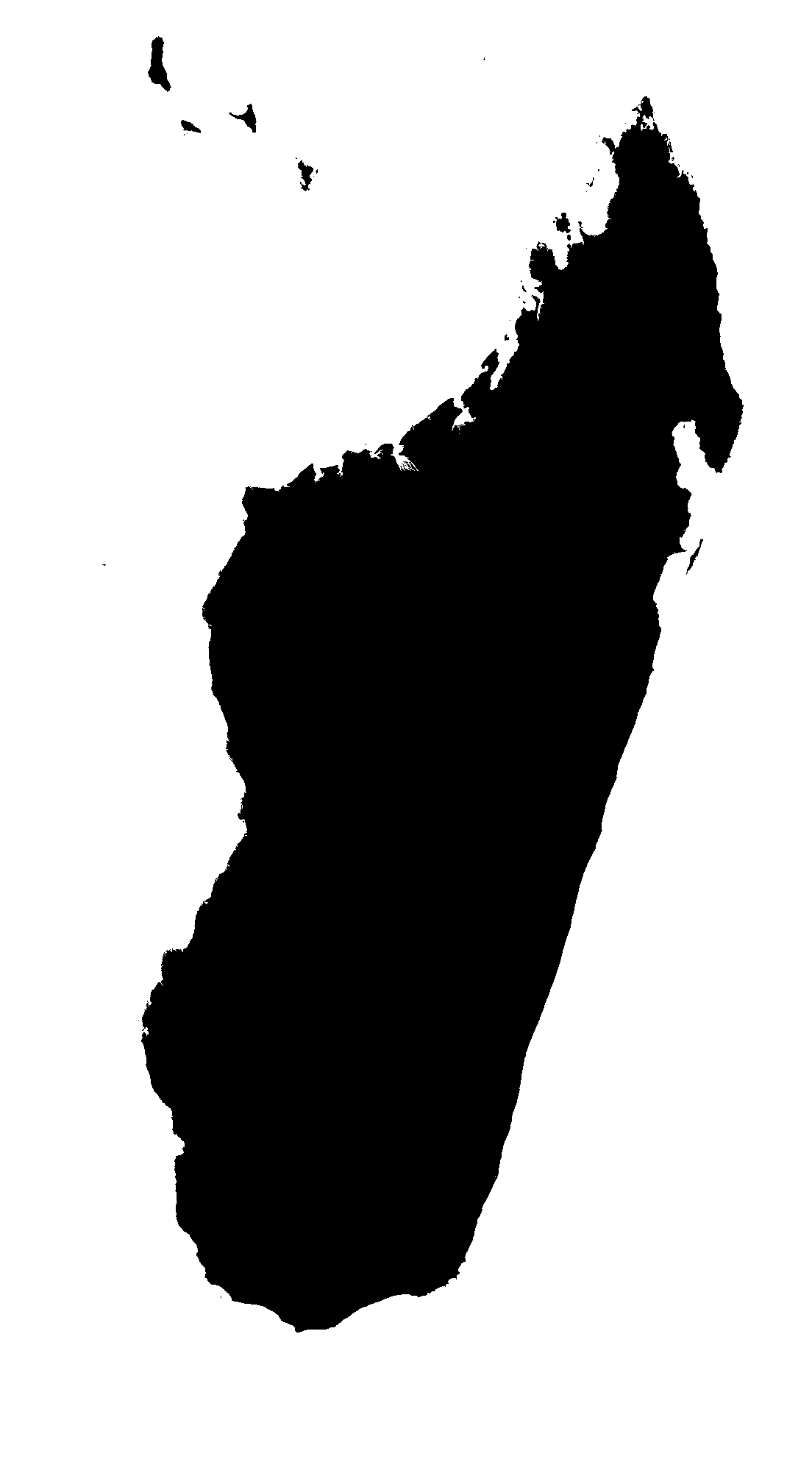

Supplement: Supplemental Information 4 [file peerj-05-4095-s004.zip › example_data/Biodiversity_measurements/biodiversity_binary_SDMs/Binary_SDMs/Uruloke_legolasvirenphyllos_prunned.tif]

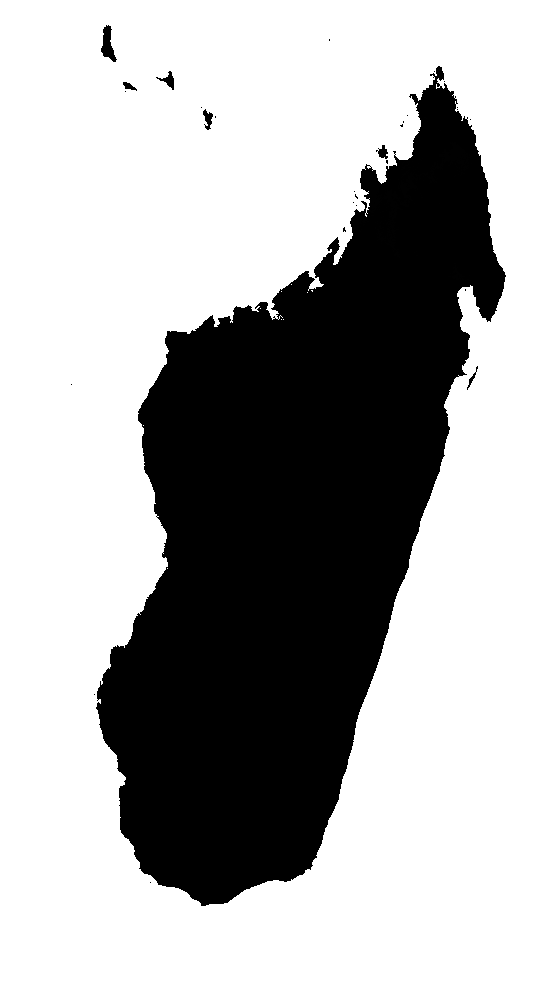

Supplement: Supplemental Information 4 [file peerj-05-4095-s004.zip › example_data/Biodiversity_measurements/biodiversity_binary_SDMs/Binary_SDMs/Uruloke_legolasvirenphyllos_prunned.tif.ovr]

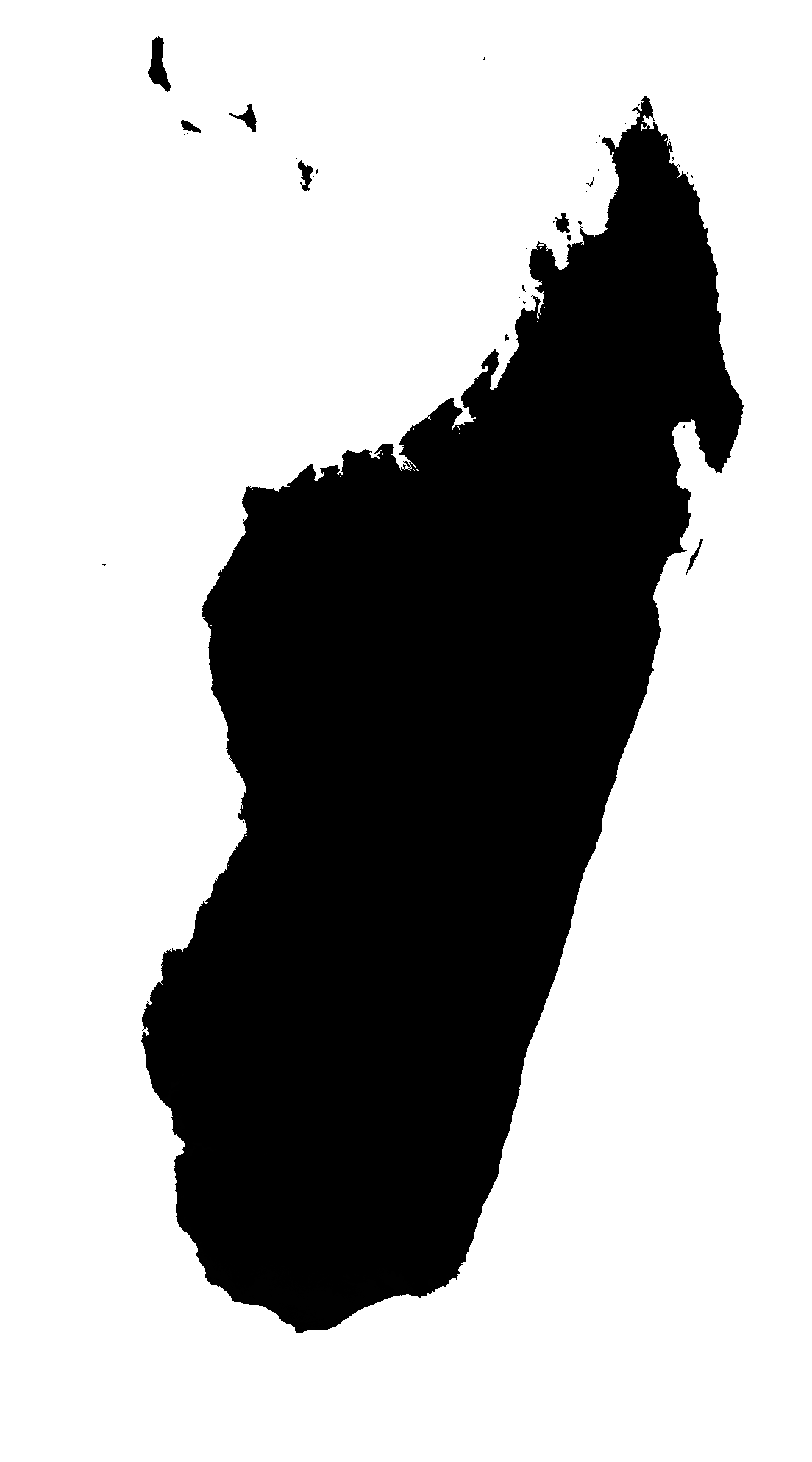

Supplement: Supplemental Information 4 [file peerj-05-4095-s004.zip › example_data/Biodiversity_measurements/biodiversity_binary_SDMs/Binary_SDMs/Uruloke_lorienensis_prunned.tif]

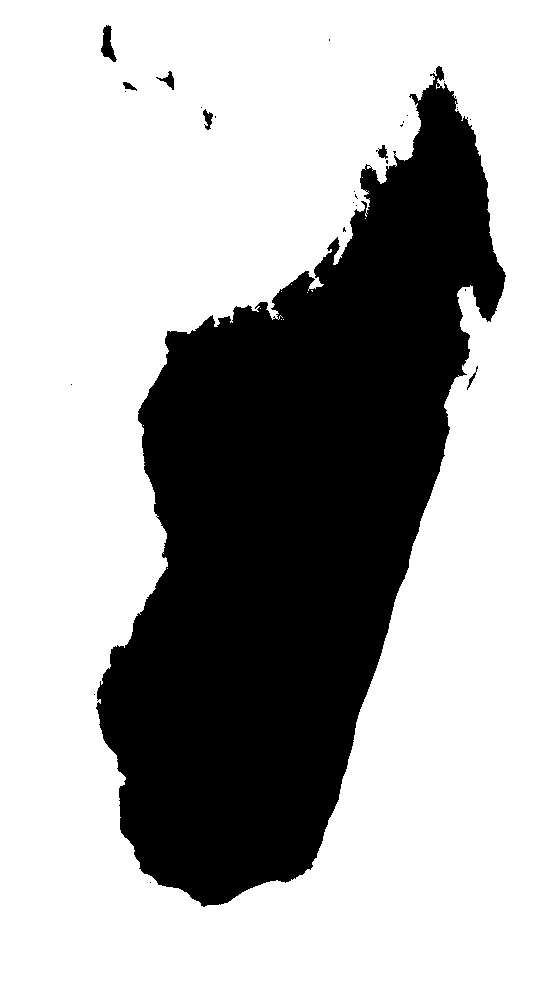

Supplement: Supplemental Information 4 [file peerj-05-4095-s004.zip › example_data/Biodiversity_measurements/biodiversity_binary_SDMs/Binary_SDMs/Uruloke_lorienensis_prunned.tif.ovr]

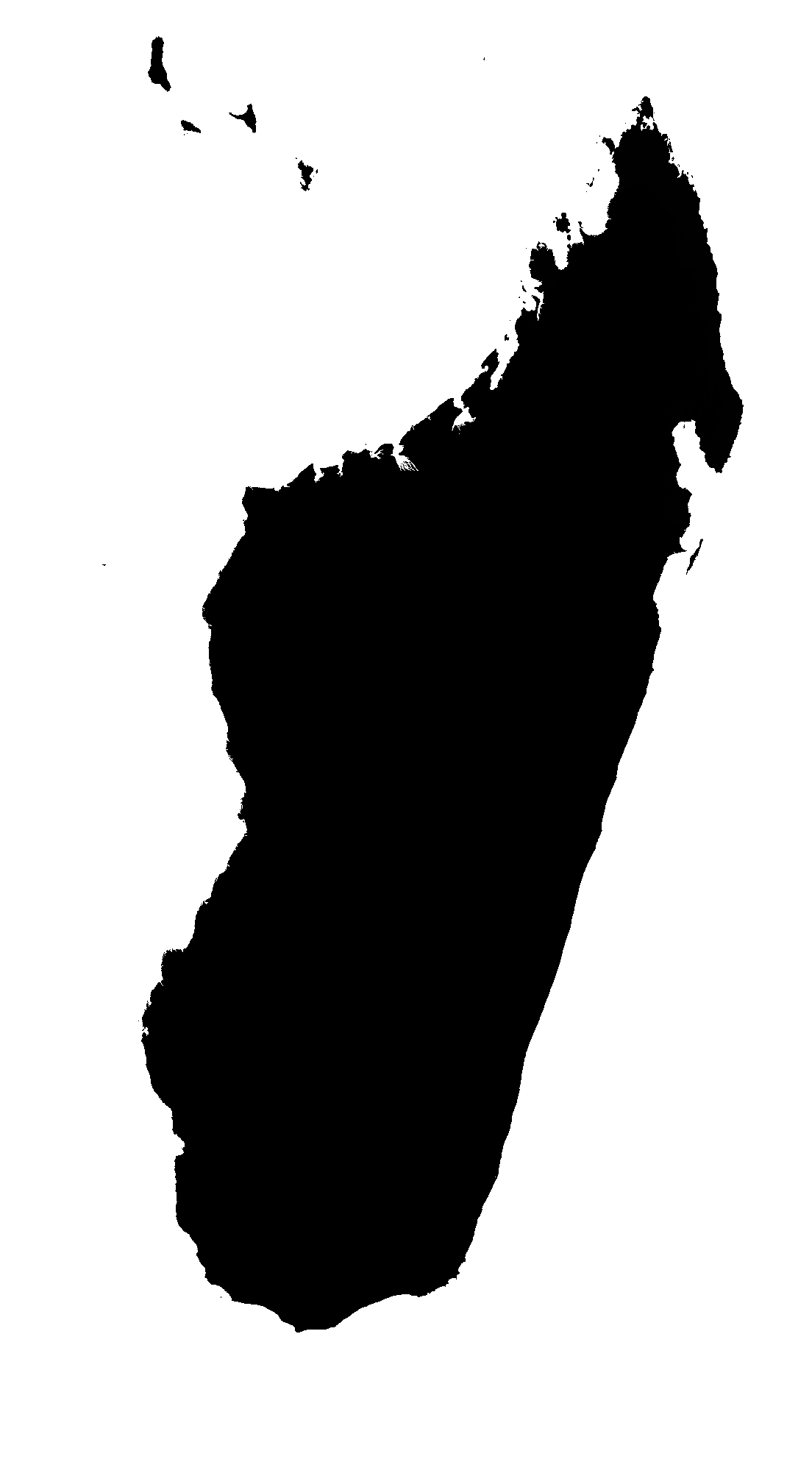

Supplement: Supplemental Information 4 [file peerj-05-4095-s004.zip › example_data/Biodiversity_measurements/biodiversity_binary_SDMs/Binary_SDMs/Uruloke_mordorensis_prunned.tif]

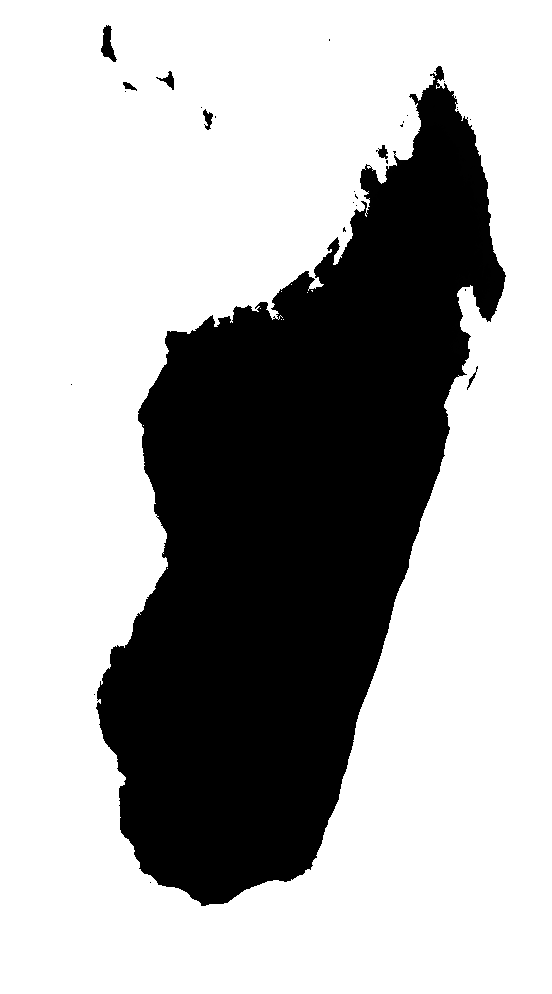

Supplement: Supplemental Information 4 [file peerj-05-4095-s004.zip › example_data/Biodiversity_measurements/biodiversity_binary_SDMs/Binary_SDMs/Uruloke_mordorensis_prunned.tif.ovr]

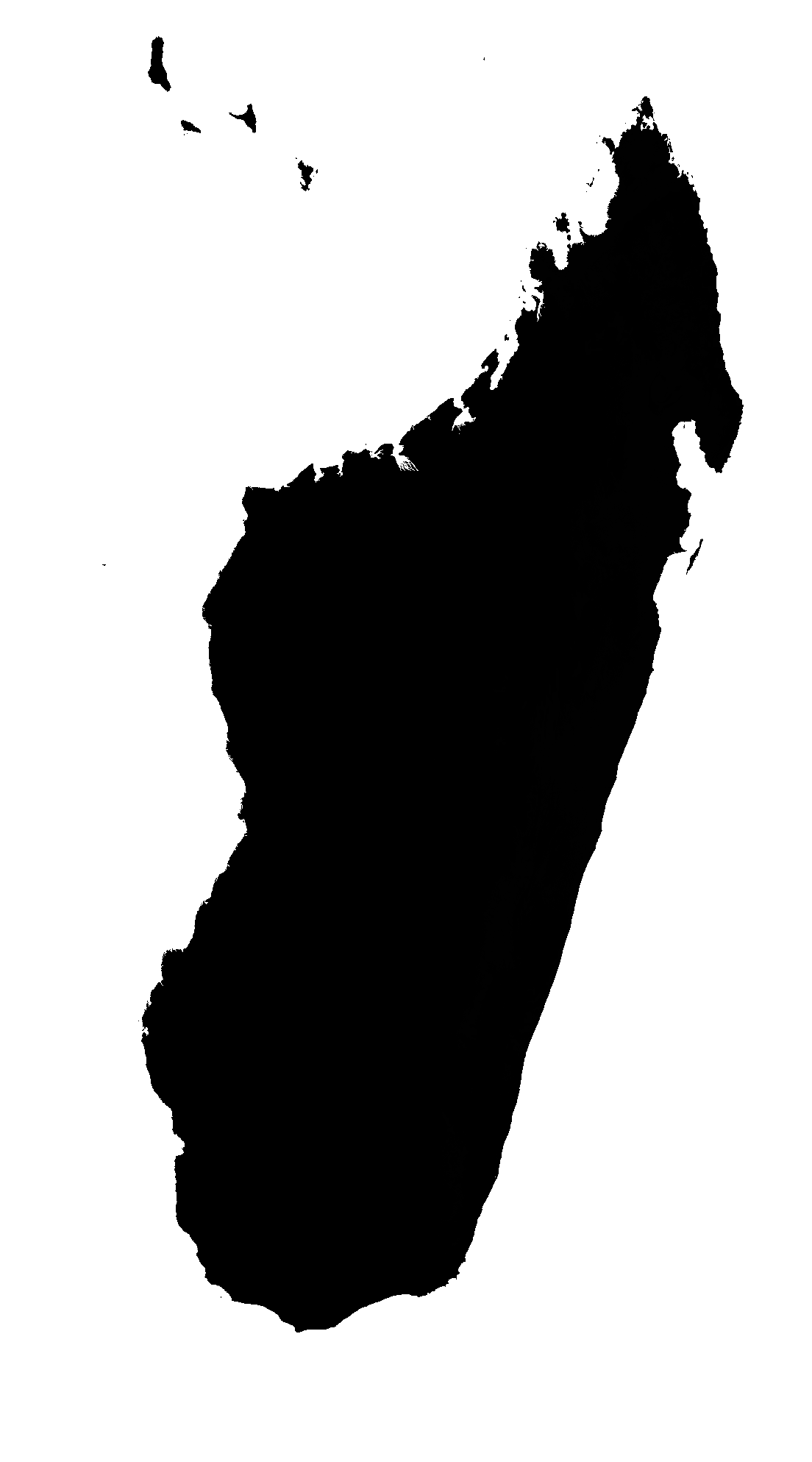

Supplement: Supplemental Information 4 [file peerj-05-4095-s004.zip › example_data/Biodiversity_measurements/biodiversity_binary_SDMs/Binary_SDMs/Uruloke_rohanensis_prunned.tif]

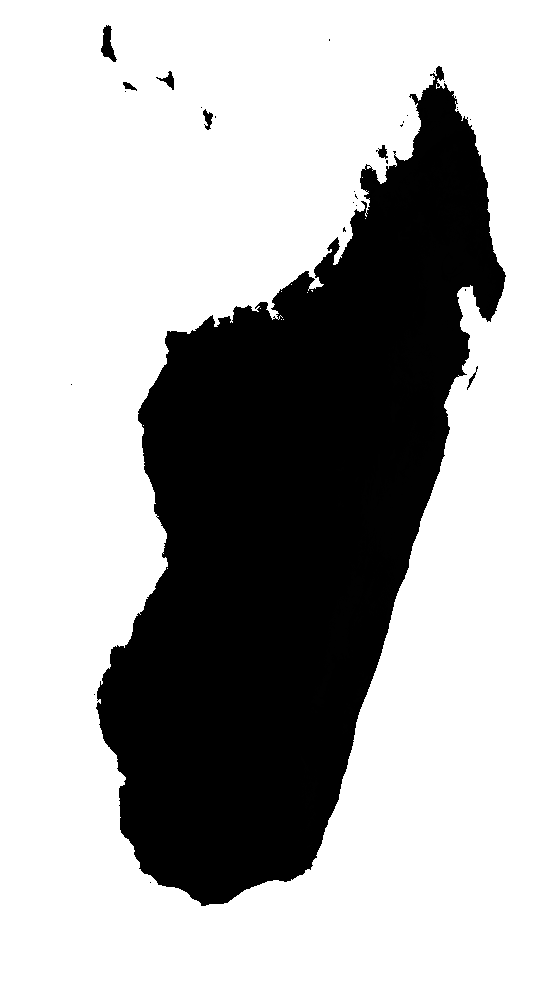

Supplement: Supplemental Information 4 [file peerj-05-4095-s004.zip › example_data/Biodiversity_measurements/biodiversity_binary_SDMs/Binary_SDMs/Uruloke_rohanensis_prunned.tif.ovr]

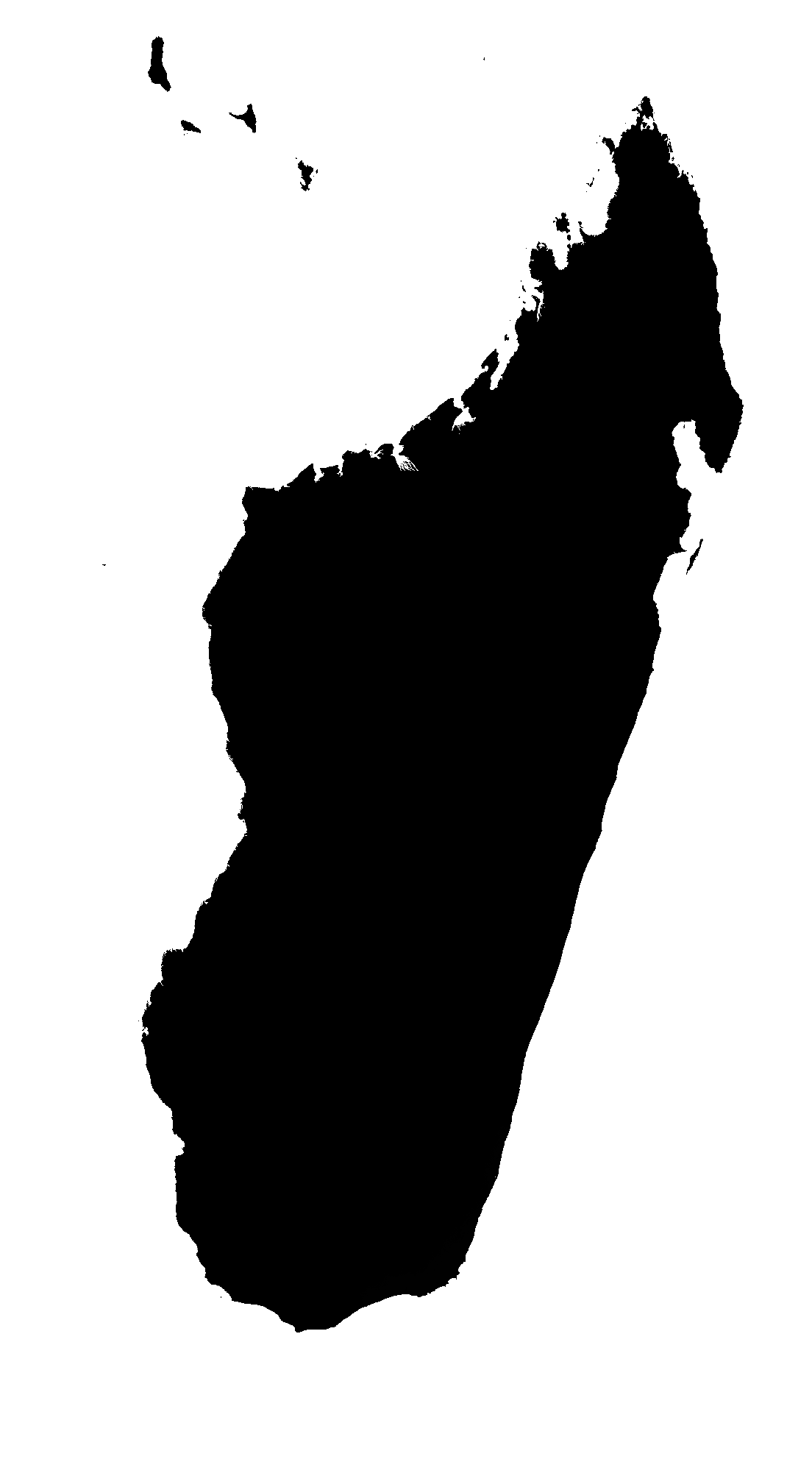

Supplement: Supplemental Information 4 [file peerj-05-4095-s004.zip › example_data/Biodiversity_measurements/biodiversity_binary_SDMs/Binary_SDMs/Uruloke_shirecola_prunned.tif]

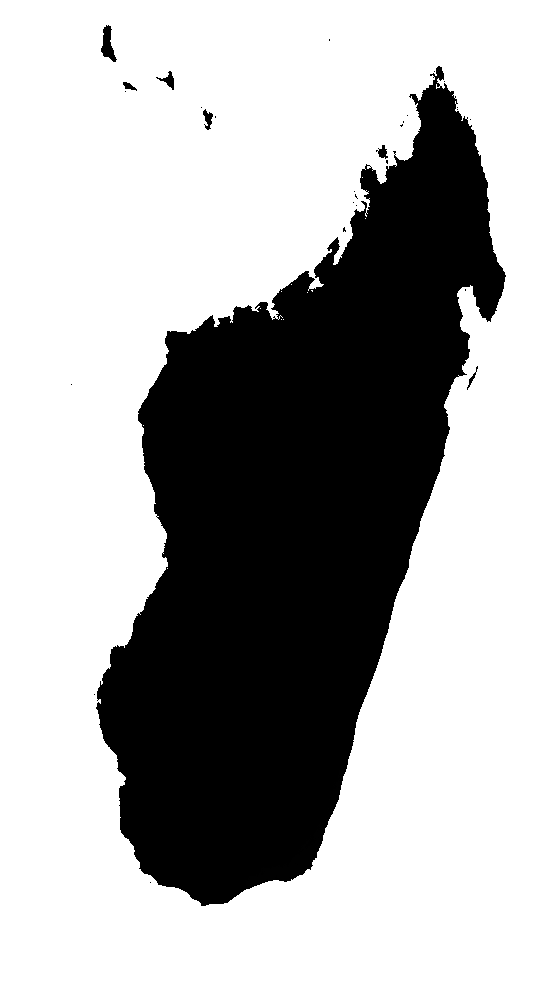

Supplement: Supplemental Information 4 [file peerj-05-4095-s004.zip › example_data/Biodiversity_measurements/biodiversity_binary_SDMs/Binary_SDMs/Uruloke_shirecola_prunned.tif.ovr]
